# Tubulin autoregulation tunes microtubule dynamics to support multicellular architecture and viability

**DOI:** 10.1038/s41467-026-75341-w

**Published:** 2026-07-22

**Authors:** Ana C. Almeida, Chahrazed Lacheheub, Ivana Gasic

**Affiliations:** Department of Molecular and Cellular Biology, University of Geneva, Geneva, Switzerland

**Keywords:** Microtubules, Cell adhesion, Cellular imaging

## Abstract

Alpha- and beta-tubulin heterodimers dynamically assemble into microtubules, key cytoskeletal elements involved in intracellular trafficking, cell adhesion, and division. The availability of free tubulins regulates the synthesis of new subunits. In response to excessive soluble αβ-tubulins, tetratricopeptide protein 5 (TTC5) selectively recognizes nascent tubulins at the ribosome, recruiting downstream effectors that degrade their encoding messenger RNAs, in a process known as tubulin autoregulation. Despite its well-characterized molecular framework, the biological relevance of this regulatory pathway remains unknown. Here, using human 3D cellular models, advanced optics, and genetic perturbation of tubulin biosynthesis, we reveal that loss of TTC5-dependent tubulin autoregulation elevates soluble tubulin levels, increasing microtubule stability and disrupting cytoskeletal organization. These defects impair the localization of adhesion molecules at cell-cell junctions and extracellular matrix interfaces, compromising tissue architecture and reducing overall cell viability. Our findings establish tubulin autoregulation as a critical mechanism that tunes microtubule dynamics to sustain cellular integrity and tissue homeostasis.

## Abstract (web summary)

Almeida AC et al. uncover the physiological role of a long-elusive feedback pathway that regulates tubulin biosynthesis. By tuning microtubule dynamics, this mechanism preserves cell adhesion, survival, and multicellular architecture.

## Introduction

Microtubules, composed of α- and β-tubulin heterodimers (henceforth αβ-tubulins), are key cytoskeletal elements that provide structural support, facilitate intracellular transport, and ensure accurate cell division^1–3^. These functions critically depend on the precise yet evolving control of αβ-tubulin levels and their spatial distribution. The best-characterized microtubule regulatory mechanism is their intrinsic dynamic instability, manifested by stochastic exchanges of polymerized and soluble αβ-tubulin subunits^4^. This property, regulated by a plethora of microtubule-associated proteins (MAPs), allows continuous remodeling of the microtubule cytoskeleton^5–7^. Much less understood is how cells define and maintain the correct supply of αβ-tubulin proteins needed to build a microtubule network tailored to cellular demands.

Evidence from in vitro work suggests that microtubule nucleation, polymerization, and dynamics scale with the concentration of αβ-tubulins^8–12^. Whether this also holds in cells remains unknown. The existence of a conserved and sophisticated feedback mechanism that controls tubulin biosynthesis suggests that maintaining precise αβ-tubulin abundance is biologically relevant.

When in surplus, soluble αβ-tubulins trigger a pathway named tubulin autoregulation, which selectively destabilizes tubulin-encoding mRNAs^13,14^. This process is mediated by TTC5 that specifically recognizes nascent α- and β-tubulins at the ribosomal exit tunnel via their conserved amino-terminal tetrapeptides^15–18^. Upon recognition, TTC5 recruits the adaptor protein SCAPER (S-Phase Cyclin A Associated Protein in the ER) and effector CCR4-NOT complex (Carbon Catabolite Repression-Negative On TATA-less) to tubulin-translating ribosomes, initiating mRNA deadenylation and decay^19,20^. Soluble αβ-tubulins directly input into pathway activity via reversible sequestration of TTC5: under steady-state conditions, αβ-tubulins bind and maintain TTC5 in an inactive state. When αβ-tubulin levels rise, TTC5 is released and activates tubulin autoregulation machinery^21^.

Although tubulin autoregulation was discovered over four decades ago, its core molecular mediators have only recently been identified^19–21^. The biological function of tubulin autoregulation, however, remains largely unexplored. Experimental activation of this pathway has historically relied on artificially elevating soluble αβ-tubulins using microtubule-depolymerizing agents such as colchicine^13,14^ or direct tubulin injection^22^, thereby obscuring its physiological role.

Altered expression and mutations in core mediators of tubulin autoregulation, TTC5 and SCAPER, have been associated with diverse cancers (*COSMICv101*^23^, *GRCh38*), ciliopathies, and neurodevelopmental disorders, such as tubulinopathies^24–27^, hinting that tubulin autoregulation may play a role in maintaining cellular homeostasis.

Tubulin mRNA levels fluctuate in response to various physiological and pathological stimuli, including nutrient deprivation or oncogenic transformation, suggesting that tubulin gene expression dynamically adapts to cellular demands^28,29^. Surprisingly, studies in human cultured cells lacking tubulin autoregulation reported no detectable changes in tubulin levels^19^, despite clear chromosome segregation defects during cell division^19–21^. These findings were conducted in 2D bulk cell cultures, where subtle, cell-cycle-specific, or context-dependent effects may be masked. Indeed, tubulin expression is influenced by culture conditions, including spatial organization (2D versus 3D)^28^ and interactions with extracellular matrix (ECM)^30^, emphasizing the need to assess tubulin biosynthesis control in more complex tissue-like models.

While conventional 2D cell cultures have been instrumental in advancing our understanding of microtubule biology, they offer limited spatial and mechanical cues, which can influence cell adhesion, shape, and protein dynamics^31,32^. To better recapitulate cell-cell interactions and microenvironmental gradients present in vivo, we employ 3D spheroid models. Spheroids provide a reproducible and tractable system to study fundamental biological problems in a multicellular context^33–35^. Their simplicity, scalability, and amenability to genetic manipulation make them well-suited for functional dissection of tubulin autoregulation and its role in maintaining tissue-like architecture and integrity.

## Results

### Tubulin autoregulation maintains steady-state tubulin levels and microtubule dynamics

**Fig. 1:**
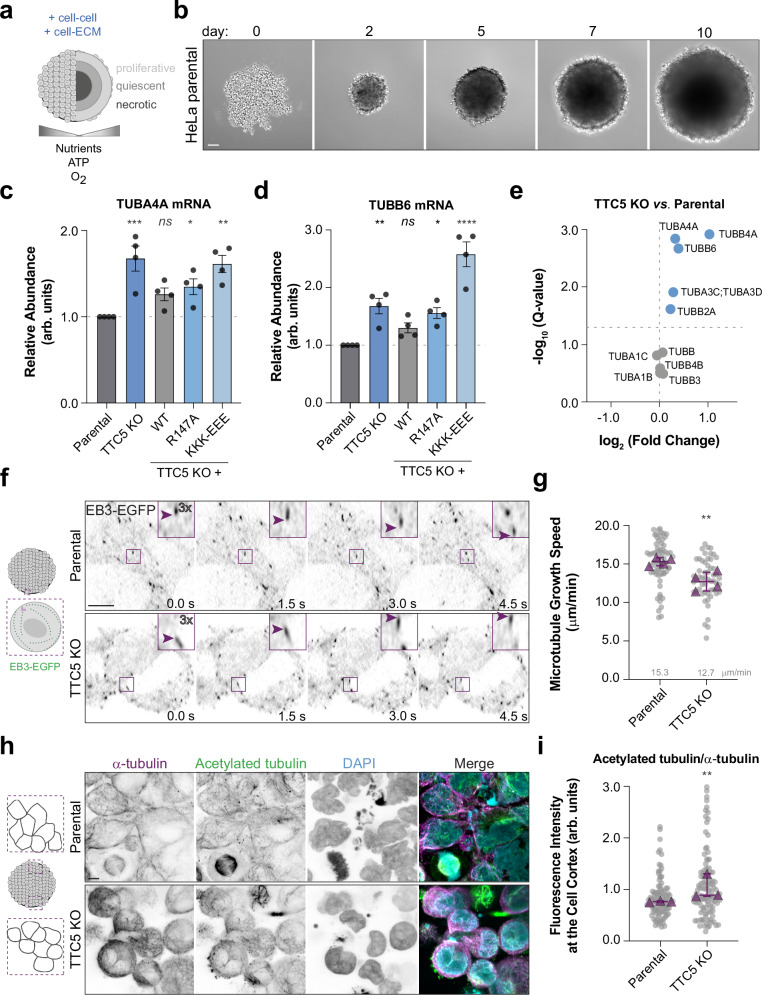
Tubulin autoregulation maintains steady-state tubulin levels and microtubule dynamics. **a** Schematic representation of the spheroid model. **b** Representative brightfield images of HeLa parental spheroids cultured for 10 days (minimum of three biological replicates). Scale bar, 100 μm. **c** Relative abundance of TUBA4A and **d** TUBB6 mRNA in HeLa parental, TTC5 knockout, and the indicated Flag-TTC5 cell lines in 10-day-old spheroids, normalized to housekeeping transcripts and parental levels. Data show mean ± SEM from four independent experiments. *p* values were determined by one-way ANOVA followed by Holm-Šidák’s multiple comparisons test, comparing each indicated cell line to the parental line. **e** Volcano plot showing the relative abundance of α- and β-tubulin isotypes in TTC5 KO versus parental spheroids, measured by quantitative mass spectrometry. Horizontal dashed line indicates *Q*-value of 0.05 for three biological replicates. **f** Time-lapse imaging of EB3-EGFP in HeLa parental and TTC5 KO spheroids, showing microtubule plus-end tracking (1.5-seconds interval). Magenta arrowhead follows a representative microtubule growth event over time. Scale bar, 5 μm. **g** Microtubule growth speed in interphase parental and TTC5 KO cells, measured over four to six consecutive frames. Data show mean ± SD from four independent experiments (Parental, *n* = 65; TTC5 KO, *n* = 54 cells). *p* value indicates an unpaired two-tailed Student’s *t-*test comparing means between TTC5 KO and the parental spheroids. **h** Immunofluorescence of 10-day-old HeLa parental and TTC5 KO spheroids stained for total α-tubulin (magenta), acetylated α-tubulin (green), and DNA (DAPI, cyan). Scale bar, 5 μm. **i** Quantification of acetylated α-tubulin/total α-tubulin intensity at the cell cortex (defined by actin localization, see Fig. S3d). Data are shown as median ± IQR from three independent experiments (Parental, *n* = 96; TTC5 KO, *n* = 96 cells). *p* value was determined by the Kolmogorov-Smirnov test comparing the distribution of TTC5 KO with parental spheroids. ^****^p < 0.0001, ^***^p < 0.001, ^**^p < 0.01, ^*^p < 0.05, ns - not significant. Exact *p* values can be found in the Source Data file.

To explore tubulin autoregulation in a multicellular model system, we leveraged HeLa cells that self-assemble into spheroids during proliferation^36^. Reliant on extensive cell-cell and cell-ECM interactions, spheroids typically organize into a stratified structure: a proliferative outer rim, a quiescent middle layer, and a necrotic core caused by oxygen and nutrient deprivation^37,38^ (Fig. 1a). Using brightfield imaging, we captured distinct spheroid development phases: spheroid aggregation and compaction (days 0–2), and growth (days 5–10)^39,40^ (Fig. 1b).

We first confirmed that tubulin autoregulation responds to its known activator in spheroids by quantifying mRNA levels upon 6 hours of colchicine treatment. TUBA and TUBB transcripts decreased in parental and TTC5 knockout (KO) spheroids that re-expressed wild-type TTC5 (TTC5^WT^), but not in TTC5 KO or spheroids expressing TTC5 mutants that abolish its function in tubulin mRNA decay: the nascent tubulin peptide- and ribosome-binding mutants (TTC5^R147A^ and TTC5^KKK-EEE^, henceforth autoregulation-incompetent mutants) (Fig. S1a, b). These results mirror previous reports in 2D cell cultures^19^.

Strikingly, even untreated 10-day-old TTC5 KO and autoregulation-incompetent mutant spheroids showed a reproducible increase in tubulin mRNA for several (TUBA4A, TUBA3C, TUBB6, TUBB4A; Figs. 1c, d and S1c, d), albeit not all tested tubulin isotypes (TUBA1B and TUBB; Fig. S1e, f). We followed two representative transcripts (TUBA4A and TUBB6) across spheroid development from day 2 to day 10 and observed that this increase was maintained throughout spheroid growth (Fig. S1g, h).

To determine whether protein levels followed a similar trend, we analyzed samples by mass spectrometry. This revealed a significant enrichment of five out of ten tubulin isotypes in TTC5 KO spheroids (TUBA4A, TUBA3C/D, TUBB4A, TUBB2A, and TUBB6; Fig. 1e), while others were not significantly altered. Notably, the magnitude of increase for the affected isotypes was substantial (23–103% more), and changes at the mRNA levels showed strong concordance with changes at the protein levels (Fig. S1i). These data indicate that loss of tubulin autoregulation leads to selective rather than uniform changes in tubulin isotype abundance.

Immunoblotting for α- and β-tubulin corroborated a modest overall trend towards increased total tubulin levels in autoregulation-incompetent mutants (Fig. S2a, b), consistent with the averaging of isotype-specific effects across the entire tubulin pool. Notably, a similar overall increase in total tubulin levels was observed in HEK293 spheroids, ruling out cell line-specific effects (Fig. S2c, d). Together, these data support the hypothesis that tubulin autoregulation contributes to the tuning of total tubulin protein levels through substantial changes in a subset of tubulin isotypes.

To test the functional consequences of increased tubulin quantity, we analyzed microtubule dynamics in 5-day-old spheroids transiently expressing comparable levels of EGFP-tagged End-binding protein 3 (EB3-EGFP, Fig. S3a). EB3 comet dynamics were quantified over four to six consecutive frames (4.5–7.5 s), revealing a 17% reduction in microtubule growth speed (15.3 μm/min versus 12.7 μm/min) (Fig. 1f, g) and 18% shorter growth length (Fig. S3b) (1.6 μm versus 1.3 μm) in TTC5-deficient cells, indicating altered microtubule dynamics.

Since modified dynamics are often linked to network rearrangement, we examined microtubule distribution in spheroids. Confocal imaging showed a redistribution of the microtubule density towards the cortex of TTC5 KO cells (Fig. 1h). Consistently, immunostaining for acetylated α-tubulin, a tubulin post-translational modification enriched on long-lived microtubules^41^, showed increased cortical acetylation in a subset of autoregulation-deficient cells (Figs. 1h, i and S3c, d).

To further probe microtubule stability, we performed a nocodazole shock (1 or 2 μM) in spheroids stably expressing EB3-EGFP and quantified EB3 comet density per unit area, as a proxy for microtubule numbers (Fig. S3e). Within 1 hour of treatment, comet density decreased with increasing nocodazole concentration in both conditions. This reduction was, however, less pronounced in TTC5 KO cells (approximately 15% versus 30% in parental cells at 2 μM nocodazole; Fig. S3f), consistent with increased resistance to microtubule depolymerization.

These findings establish that tubulin autoregulation is critical to maintain αβ-tubulin concentration and microtubule dynamics. The selective increase in tubulin mRNA and protein levels observed in both TTC5-deficient and -incompetent mutant spheroids confirms that these changes arise specifically from impaired tubulin autoregulation.

### Loss of tubulin autoregulation disrupts spheroid architecture and compromises cell viability

**Fig. 2:**
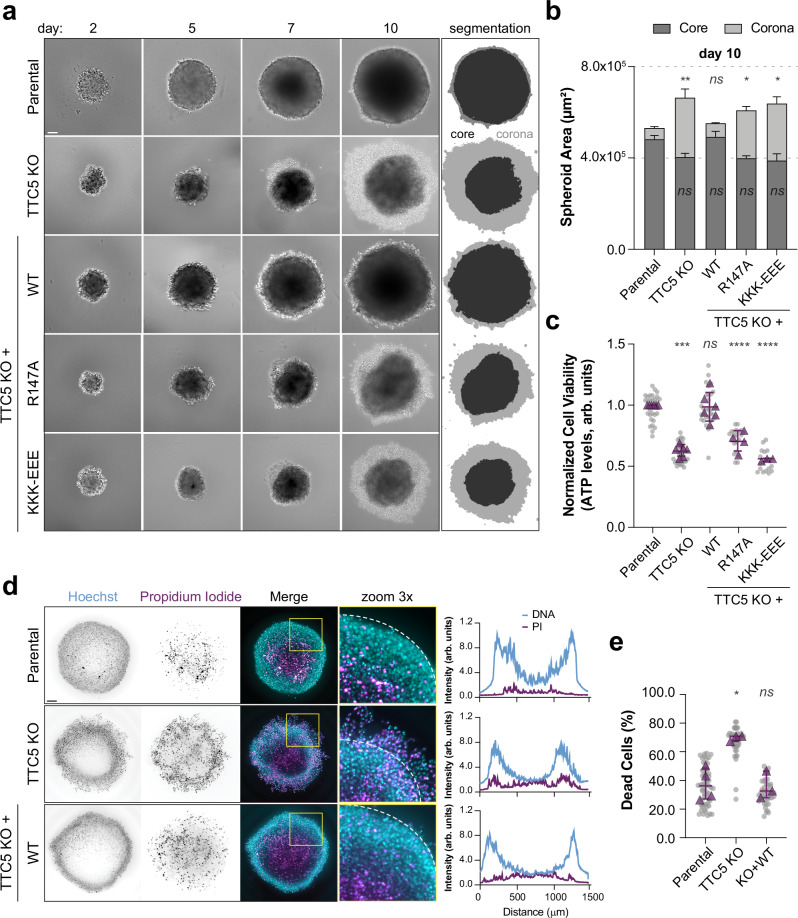
Tubulin autoregulation is required for normal spheroid growth and viability. **a** Representative brightfield images of spheroids from HeLa parental, TTC5 KO, and the indicated Flag-TTC5 rescue cell lines (day 2 to day 10). Scale bar, 100 μm. Trainable Weka Segmentation masks are shown: core (dark grey), corona (light grey). **b** Spheroid core and corona areas measured at day 10. Data indicate mean ± SEM from a minimum of three biological replicates (Parental, *n* = 21; TTC5 KO, *n* = 23; TTC5^WT^, *n* = 25; TTC5^R147A^, *n* = 28; TTC5^KKK-EEE^, *n* = 22 spheroids). *p* values were determined by one-way ANOVA followed by Holm-Šidák’s multiple comparisons test, comparing the indicated cell lines to the parental line. **c** Cell viability determined by ATP content across the indicated cell lines, normalized to parental spheroids. Data show mean ± SD from a minimum of three independent experiments (Parental, *n* = 31; TTC5 KO, *n* = 31; TTC5^WT^, *n* = 31; TTC5^R147A^, *n* = 20; TTC5^KKK-EEE^, *n* = 17 spheroids). *p* values were determined by one-way ANOVA followed by Holm-Šidák’s multiple comparisons test relative to the parental cell line. **d** Representative stills of HeLa parental, TTC5 KO, and TTC5^WT^ rescue spheroid labelled with Hoechst (all nuclei, cyan) and Propidium Iodide (PI, dead cells, magenta). Scale bar, 100 μm. Insets show 3x magnification emphasizing the spheroid core-corona transition (white dashed line). Graphs on the right show line scans measurements highlighting the distribution of all nuclei (cyan) and dead cells (magenta) across the spheroid diameter. **e** Percentage of dead cells (PI-positive) from all segmented nuclei measured at day 10 in HeLa parental, TTC5 KO and TTC5^WT^ spheroids. Data are shown as median ± IQR from a minimum of three independent experiments (Parental, *n* = 57; TTC5 KO, *n* = 42; WT, *n* = 41 spheroids). *p* values were calculated using Kruskal-Wallis test followed by Dunn’s multiple comparisons test, comparing TTC5 KO and TTC5^WT^ to parental spheroids. ^****^p < 0.0001, ^***^p < 0.001, ^**^p < 0.01, ^*^p < 0.05, ns - not significant. Exact *p* values can be found in the Source Data file.

To investigate the broader consequences of impaired microtubule dynamics, we monitored spheroid growth from days 2 to 10 in parental cells and cells deficient in tubulin autoregulation (Fig. 2a). Using a trainable segmentation tool (see “Methods” for details on the quantification), we divided each spheroid into two regions, a dense central core and a less dense outer layer (Fig. 2a “segmentation”). Over time, control spheroids retained a prominent core surrounded by a thin outer layer. In contrast, TTC5 KO spheroids exhibited progressive expansion, forming a large area of dispersed cells surrounding the spheroid, which we termed the corona. This phenotype was rescued by TTC5^WT^ re-expression (Fig. 2a, b). By day 10, the corona area in the absence of TTC5 was substantially expanded, leading to a marked increase in the overall spheroid size and prompting us to perform most phenotypic analyzes at this later time point. Notably, TTC5 KO spheroids re-expressing tubulin autoregulation-incompetent mutants phenocopied the knockout (Figs. 2a, b and S4a). Similar architectural abnormalities were reproduced in an independent TTC5 KO clone (Fig. S4b, c), strongly implicating loss of tubulin autoregulation as the cause of these defects.

To assess the impact on cell viability, we monitored ATP levels—as proxy of active cellular metabolism and survival—and observed a 40-50% reduction in TTC5 KO and autoregulation-incompetent mutant spheroids compared to controls (Fig. 2c). Because ATP levels alone cannot distinguish between differences in cell number and cell viability, we stained spheroids with Hoechst (to label all nuclei) and Propidium Iodide (PI, to label dead cells), thus gaining a more precise and spatial insight into the distribution of viable and non-viable cells. In control and TTC5^WT^ spheroids, dead cells were mostly confined to the spheroid center (Fig. 2d), consistent with the expected oxygen- and nutrient-limited conditions. In contrast, TTC5-deficient spheroids displayed dispersed dead cells across the spheroid, particularly enriched at the corona (Fig. 2d). Quantification of PI-positive cells revealed a two-fold increase in the percentage of dead cells in TTC5 KO spheroids compared to parental and TTC5^WT^ spheroids (Fig. 2e).

Halted progression through the cell cycle may contribute to increased cell death^42^. To test this hypothesis, we analyzed DNA content by flow cytometry (Fig. S5a–e). This analysis revealed no significant changes across the different cell lines (Fig. S6a), consistent with prior reports showing that TTC5-deficient cells complete mitosis within a similar timeframe as parental cells^19^. Supporting these observations, pharmacological inhibition of mitotic entry using the cyclin-dependent kinase 1 (Cdk1) inhibitor Ro-3306 did not prevent corona expansion in TTC5 KO spheroids (Fig. S6b, c).

One possibility was that peripheral cell death in autoregulation-deficient spheroids resulted from passive necrotic core disintegration. To test this hypothesis, we tracked individual living cells using Hoechst and PI. Time-lapse imaging showed that cell detachment and accumulation at the spheroid corona preceded cell death (Fig. S6d, yellow arrowhead). The probability of cell survival at the spheroid corona reduced over time, with approximately 80% of the cells dying after losing attachment (Fig. S6e). These data rule out the necrotic core dispersion as a likely driver of the observed phenotype. In addition, a spheroid migration assay revealed no evidence of enhanced migration capacity in TTC5-deficient cells (Fig. S6f–h).

Together, these data indicate that loss of tubulin autoregulation compromises multicellular architecture and cell viability, not through altered proliferation or invasion, but possibly via impaired cell adhesion.

### Tubulin autoregulation ensures proper cell-cell and cell-ECM adhesion in 3D assemblies

**Fig. 3:**
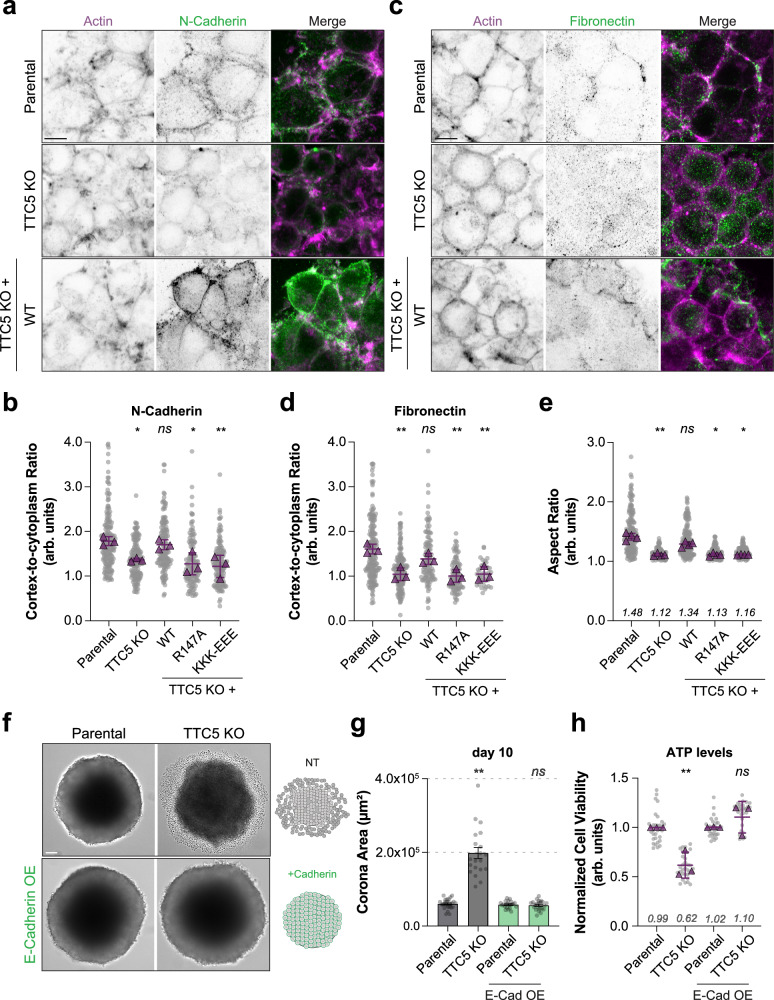
Tubulin autoregulation ensures proper cell-cell and cell-ECM adhesion in 3D assemblies. **a** Representative confocal images of 10-day-old HeLa parental, TTC5 KO and TTC5^WT^ spheroids immunostained for N-Cadherin (green) and counterstained with phalloidin-647 (magenta, Actin). Scale bar, 5 μm. **b** Quantification of N-Cadherin fluorescence intensity (cortex-to-cytoplasm ratio, see Fig. S7a). Data are shown as mean ± SD from three independent experiments (Parental, *n* = 176; TTC5 KO, *n* = 154; TTC5^WT^, *n* = 140; TTC5^R147A^, *n* = 119; TTC5^KKK-EEE^, *n* = 135 cells). *p* values indicate one-way ANOVA followed by Holm-Šidák’s multiple comparisons test, comparing each of the indicated cell lines with the parental line. **c** Confocal images of spheroids stained for Fibronectin (green) and phalloidin-647 (magenta, Actin). Scale bar, 5 μm. **d** Fluorescence intensity of Fibronectin. Data are shown as mean ± SD from three independent experiments (Parental, *n* = 186; TTC5 KO, *n* = 163; TTC5^WT^, *n* = 105; TTC5^R147A^, *n* = 95; TTC5^KKK-EEE^, *n* = 99 cells). *p* values were determined by one-way ANOVA followed by Holm-Šidák’s multiple comparisons test, comparing the indicated cell lines to the parental line. Representative examples of TTC5^R147A^ and TTC5^KKK-EEE^ mutants are shown in Fig. S7b, c. **e** Aspect ratio measurements in HeLa parental, TTC5 KO, and the indicated Flag-TTC5 rescue cell lines. Data are shown as median ± IQR from four independent experiments (Parental, *n* = 172; TTC5 KO, *n* = 169; TTC5^WT^, *n* = 181; TTC5^R147A^, *n* = 145; TTC5^KKK-EEE^, *n* = 185 cells). *p* values were calculated using the Kruskal-Wallis test followed by Dunn’s multiple comparisons test of each of the indicated cell lines with the parental line as reference. **f** Brightfield images of 10-day-old wildtype and E-Cadherin overexpressing HeLa parental and TTC5 KO spheroids. Scale bar, 100 μm. **g** Spheroid corona area quantification. Mean ± SEM from a minimum of three independent experiments (Parental (NT, *n* = 26; E-Cad OE, *n* = 22); TTC5 KO (NT, *n* = 20; E-cad OE, *n* = 22 spheroids). *p* values were determined by one-way ANOVA followed by Holm-Šidák’s multiple comparisons test, comparing TTC5 KO to parental spheroids. **h** Cell viability determined by ATP content across the indicated cell lines, normalized to parental spheroids within each condition. Data are shown as mean ± SD from three independent experiments (Parental NT, *n* = 32; E-Cad OE, *n* = 3; TTC5 KO NT, *n* = 31; E-cad OE, *n* = 30 spheroids). *p* values were determined by one-way ANOVA followed by Holm-Šidák’s multiple comparisons test, comparing TTC5 KO to parental spheroids. ^**^p < 0.01, ^*^p < 0.05, ns - not significant. Exact *p* values can be found in the Source Data file.

Cell adhesion is essential for the organization of individual cells into 3D assemblies^43^. Microtubules play a key role in this process by delivering regulatory factors, scaffolding signaling molecules, and directly interacting with adhesion components to coordinate cell-cell and cell-matrix adhesion^44^. We hypothesize that disruption in tubulin levels and microtubule dynamics may impair adhesion, potentially explaining the architectural defects observed in tubulin autoregulation-deficient spheroids. To test this hypothesis, we stained for bona fide adhesion proteins: N-cadherin, integrin α5, and fibronectin. Quantification of cortex-to-cytosol fluorescence ratios (Fig. S7a; see “Methods” section for a full description) showed reduced cortical localization of adhesion proteins in TTC5 KO and mutant spheroids (Figs. 3a–d and S7b–e). The levels of N-Cadherin (CDH2) and integrin α5 (ITGA5) were reduced in TTC5 KO spheroids (Fig. S7f). Importantly, these adhesion proteins do not contain the TTC5-recognition motif and are therefore unlikely to be directly cotranslationally regulated by TTC5 (Fig. S7g), implying that the observed effects are downstream consequences of disrupted tubulin homeostasis.

Consistent with loss of anchorage and weakened adhesion, autoregulation-deficient cells (TTC5 KO, TTC5^R147A^ and TTC5^KKK-EEE^) exhibited more round shapes (aspect ratio, cell major axis/minor axis ratio, ~1) than parental or TTC5^WT^ cells (Fig. 3e). The latter involves overexpression of wild-type TTC5, which may explain why these cells displayed an intermediate aspect ratio; however, no statistically significant difference was observed between TTC5^WT^ and parental cells (Fig. 3e).

To determine whether cell rounding reflected an early apoptotic response, we stained spheroids for the apoptosis marker cleaved caspase-3 (c-CASP3) and the adhesion molecule N-Cadherin. TTC5 KO cells that appeared rounded and showed reduced cortical N-cadherin staining were negative for c-CASP3, indicating that loss of adhesion precedes, rather than results from, apoptosis (Fig. S8a brown arrowhead).

Supporting the hypothesis that loss of adhesion compromises cell viability, overexpression of E-Cadherin (Fig. S8b) fully suppressed spheroid corona expansion and cell death (Fig. 3f–h). The same trend was observed when supplementing the culture medium with Matrigel, a basement membrane matrix rich in ECM components (Fig. S8c–e). Consistently, co-culturing of TTC5 KO with parental cells restored spheroid morphology (Fig. S8f) when parental cells constituted as little as 25% of the total cell population, suggesting that there is a collective input to overall spheroid adhesion.

Together, these findings establish that tubulin autoregulation is required for the localization of proteins that mediate cell–cell and cell–ECM adhesion at the plasma membrane, thereby supporting the architecture of multicellular 3D assemblies.

### Microtubule dynamics downstream of tubulin autoregulation control adhesion and tissue integrity

**Fig. 4:**
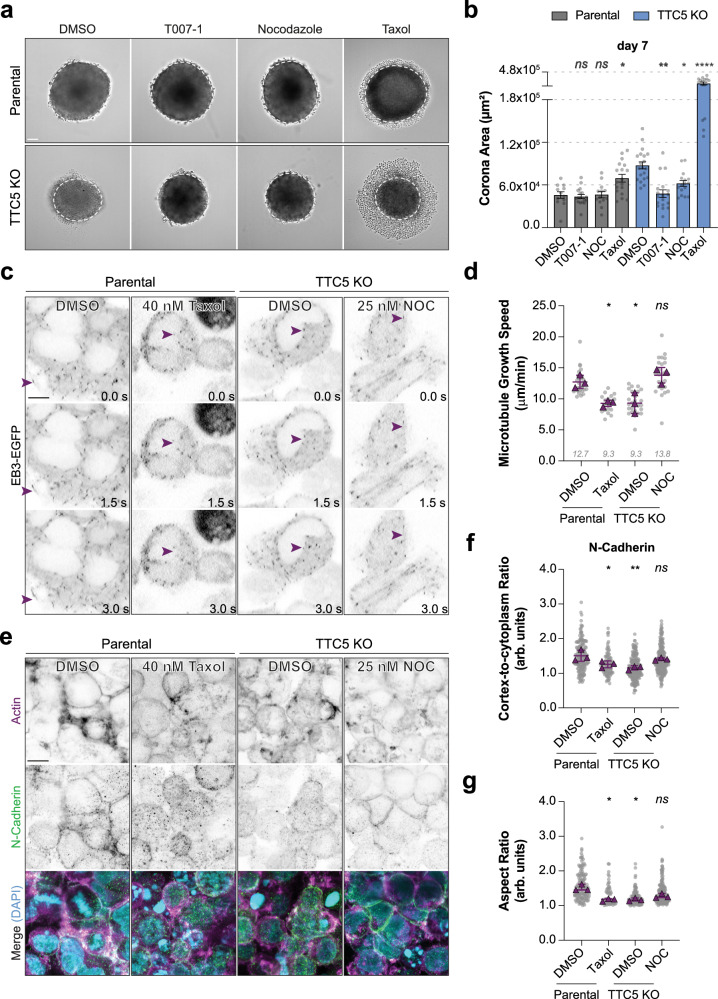
Microtubule dynamics downstream of tubulin autoregulation control adhesion and tissue integrity. **a** Brightfield images of 7-day-old HeLa parental and TTC5 KO spheroids treated for 48 hours with 1% DMSO, tubulin degrader T007-1 (400 nM), nocodazole (NOC, 25 nM), or taxol (40/20 nM, respectively). White dashed lines highlight the spheroid core-corona transition. Scale bar, 100 μm. **b** Spheroid corona areas at day 7. Data show mean ± SEM from three independent experiments (Parental DMSO, *n* = 12; T007-1, *n* = 17; NOC *n* = 13; taxol, *n* = 18; TTC5 KO DMSO, *n* = 20; T007-1, *n* = 18; NOC *n* = 16; taxol, *n* = 15 spheroids). *p* values reflect one-way ANOVA followed by HolmŠidák’s multiple comparisons test for each of the indicated treatments compared to its respective DMSO control. **c** Time-lapse imaging of HeLa parental and TTC5 KO spheroids stably expressing EB3-EGFP, showing microtubule plus-end tracking (1.5-seconds interval) after treatment with DMSO, nocodazole, or taxol for 48 hours. Magenta arrowhead follows a representative microtubule growth event over time. Scale bar, 5 μm. **d** Microtubule growth speed in interphase parental and TTC5 KO cells, measured over four to six consecutive frames. Data show mean ± SD from three independent experiments (Parental DMSO, *n* = 28, taxol, *n* = 18; TTC5 KO DMSO, *n* = 27 cells, NOC, *n* = 25). *p* values were determined by one-way ANOVA followed by Holm-Šidák’s multiple tests, comparing each condition with parental DMSO-treated spheroids. **e** Confocal images of 7-day-old HeLa parental and TTC5 KO spheroids post-treatment, stained for N-Cadherin (green), phalloidin-647 (magenta, Actin), and DAPI (cyan, DNA). Scale bar, 5 μm. **f** Cortex-to-cytoplasm N-Cadherin fluorescence intensity. Data show median ± IQR from three independent experiments (Parental DMSO, *n* = 142; taxol, *n* = 94; TTC5 KO DMSO, *n* = 219; NOC, *n* = 238 cells). *p* values were calculated using one-way ANOVA followed by Holm-Šidák’s multiple comparisons test, comparing each condition to parental DMSO-treated spheroids. **g** Aspect ratio of cells from parental and TTC5 KO spheroids upon the indicated treatments (Parental DMSO, *n* = 138; taxol, *n* = 61; TTC5 KO DMSO, *n* = 140; NOC, *n* = 180 cells). Data show median ± IQR from three independent experiments. *p* values were determined by Kruskal-Wallis test followed by Dunn’s multiple comparisons test, comparing parental DMSO-treated spheroids to the indicated conditions. ^****^p < 0.0001, ^**^p < 0.01, ^*^p < 0.05, ns - not significant. Exact *p* values can be found in the Source Data file.

To further probe the functional link between tubulin autoregulation and spheroid architecture, we directly manipulated tubulin levels and microtubule dynamics and assessed their impact on spheroid morphology. To capture early events and avoid confounding effects from corona formation and the associated decline in cell viability, treatments were initiated in 5-day-old spheroids. First, we treated spheroids with low doses of T007-1, a small-molecule that covalently modifies Cys-239 on β-tubulin and triggers proteasomal degradation of both α- and β-tubulins^45^. While no effect was observed in parental spheroids, in TTC5 KO spheroids, T007-1 treatment led to a reduction in corona area, rendering them indistinguishable from parental spheroids (Fig. 4a, b, white dashed line highlights the spheroid core-corona transition). This indicates that targeting the excess of tubulin accumulated in the absence of tubulin autoregulation can restore normal spheroid architecture.

Inducing mild microtubule destabilization with low-dose nocodazole (25 nM), which results in a significant increase in the microtubule growth speed (TTC5 KO: 9.3 μm/min versus 13.8 μm/min; Figs. 4c, d and S9a, b), reduced the corona area by ~20% in TTC5-deficient spheroids. On the contrary, treatment with low-dose taxol, a microtubule stabilizing agent which imposes slower microtubule dynamics (Parental: 12.7 μm/min versus 9.3 μm/min; Figs. 4c, d and S9a–d) and higher tubulin acetylation (Fig. S9e), led to the formation of the corona in parental spheroids, and exacerbated the phenotype in TTC5 KO spheroids, significantly increasing corona area (Fig. 4a, b). Notably, TTC5 KO cells were particularly sensitive to taxol, suggesting that excessive microtubule stabilization has detrimental effects on spheroid architecture (Fig. S9c, d). Fine-tuning microtubule dynamics, without imposing a change in tubulin levels (Fig. S9f), was sufficient to alter spheroid morphology. Moreover, low-dose nocodazole, reinstated N-Cadherin and Fibronectin cortical localization and partly rescued cell shape (Figs. 4e–g and S9g, h). In contrast, 40 nM Taxol treatment impaired N-Cadherin and Fibronectin localization, and promoted a more rounded cell morphology in parental spheroids (Figs. 4e–g and S9g, h), mimicking the phenotype found in tubulin autoregulation-deficient cells.

These results highlight that microtubule dynamics downstream of tubulin autoregulation are key for the localization of adhesion molecules. Imbalances in microtubule stability profoundly disrupt cell adhesion and 3D tissue organization, underscoring the importance of finely tuning tubulin homeostasis for maintaining tissue integrity and viability.

## Discussion

Tubulin autoregulation is a conserved feedback mechanism that maintains balanced tubulin biosynthesis by selectively degrading tubulin-encoding mRNAs when αβ-tubulin levels are excessive^46^. Recent mechanistic studies established that, when liberated from soluble αβ-tubulins, TTC5 recognizes nascent α- and β-tubulin chains during translation and, via recruitment of SCAPER and the CCR4-NOT deadenylase complex, initiates mRNA deadenylation and decay^19–21^. These discoveries have enabled the development of the first molecular tools to manipulate tubulin autoregulation in cells, opening the door to functional analyzes of this pathway.

Given its function in limiting runaway tubulin biosynthesis, tubulin autoregulation has been assumed to act as a quantity control pathway. Yet, pioneering studies relied exclusively on microtubule-targeting agents to trigger tubulin autoregulation, leaving its physiological relevance unexplored. In this study, we uncover a critical role for TTC5-mediated tubulin autoregulation in maintaining tubulin abundance, regulating microtubule dynamics, and preserving spheroid architecture. Combining genetic, biochemical, and imaging approaches, we demonstrate that loss of tubulin autoregulation leads to increased tubulin levels, disrupted microtubule dynamics, and weakened cell adhesion, therefore compromising spheroid morphology and viability. This work establishes tubulin quantity control as a core regulator of multicellular homeostasis.

Notably, our data indicate that loss of tubulin autoregulation does not result in a uniform increase across all tubulin isotypes but instead leads to selective changes in a subset of α- and β-tubulin proteins. These changes show strong concordance between mRNA and protein levels, supporting the view that autoregulation primarily acts through modulation of transcript abundance to control protein output. The selective nature of this response is reflected in the comparatively modest increase observed at the level of total tubulin, which integrates signals across all isotypes.

The extent of regulation varies across isotypes, with some showing stronger responses than others. Given that tubulin autoregulation depends on ongoing translation^17,47^, these differences likely reflect variation in translation rates and dynamics among tubulin isotypes, which would determine their exposure to TTC5-mediated recognition at the ribosome. In addition, isotype-specific features such as mRNA sequence elements or associated RNA-binding proteins^48^ may further modulate transcript stability or translational efficiency, thereby fine-tuning the autoregulatory response.

We propose a model in which TTC5-mediated response to excess unpolymerized tubulin couples translation to mRNA degradation, limiting mRNA accumulation and preventing tubulin buildup. At first glance, our findings seem at odds with previous work reporting moderately increased tubulin mRNA, yet unchanged protein levels upon loss of tubulin autoregulation in monolayer cultures^19^. However, monolayer systems may lack physiological signals that activate autoregulation, displaying minimal pathway activity at steady state. In contrast, 3D cultures such as spheroids exhibit higher autoregulation activity, allowing functional consequences to emerge. Consistent with this model, a switch from 2D to 3D culture was previously associated with differential tubulin gene expression^28^. Together, these observations suggest that tubulin autoregulation is context-dependent. Future work exploring how extracellular cues, such as ECM composition, matrix stiffness, and tissue geometry, regulate autoregulatory activity will be important to understand how tubulin output is tuned to cellular demands.

The relationship between tubulin concentration and microtubule dynamics is well established in vitro, where polymerization rate and dynamic instability scale linearly with tubulin availability^8–11^. Conversely, in cells, this link has remained elusive due to a lack of tools to selectively manipulate tubulin levels. Computational models have attempted to address this gap but generally fall short due to the large complexity of the tubulin network and the vast number of microtubule regulators^49–51^. Leveraging tubulin autoregulation as a molecular handle to increase tubulin levels, we find that even a modest increase in tubulin abundance reduces microtubule growth rate and growth length. These findings highlight a disconnect between bulk tubulin levels and polymerization dynamics in vivo and may explain previously reported defects in mitotic fidelity in tubulin autoregulation-deficient cells^19–21^.

This apparent discrepancy with in vitro behavior likely reflects the fact that, in cells, microtubule growth is not determined solely by bulk tubulin concentration but is instead limited by additional factors. These include the availability of nucleation sites, the activity of MAPs, and the partitioning of tubulin between polymerizable and non-polymerizable pools. Increased tubulin levels may therefore alter the balance of these regulatory systems, for example, by titrating MAPs or shifting the equilibrium between free and sequestered tubulin, ultimately resulting in slower plus-end growth despite higher total tubulin abundance. Similar changes in dynamics have been observed upon loss of core microtubule-associated proteins, such as EB3^52,53^, CAP-Gly Domain Containing Linker Protein 1 (CLIP1)^54^, or Cytoplasmic Linker Associated Protein 1 (CLASP1)^55^, placing tubulin levels on par with classical binding proteins in shaping microtubule behavior.

Future studies using tubulin small-molecule degraders^45,56^ to profile microtubule dynamics in regimes when tubulin building blocks are depleted could complement our approach and offer a comprehensive view of how microtubule dynamics scale with tubulin quantity in cells.

Tightly regulated dynamics are critical for proper microtubule network organization and function. Consistent with this, the increase in acetylated tubulin observed in TTC5 KO spheroids likely reflects a redistribution of stable microtubule subpopulations rather than a uniform increase in microtubule stability. As acetylation was quantified at the cell cortex, it remains possible that changes in microtubule organization or subcellular localization contribute to this phenotype.

Such architectural change is likely to disrupt directional transport and the delivery of key adhesion molecules to the plasma membrane, contributing to reduced cortical localization of cadherins and integrins^57–60^. Defective trafficking—perhaps due to altered kinesin or dynein motility along stabilized tracks—may underlie the failure to establish and maintain robust intercellular and ECM adhesions, and ultimately compromise the integrity of multicellular assemblies such as spheroids^44^. Endogenous labeling of the relevant ECM proteins and development of sophisticated imaging approaches adapted to 3D culture models will be required to test this hypothesis.

Our study provides a proof-of-principle that tubulin autoregulation is required for the integrity of multicellular assemblies. While we focused on tumor-like spheres, this pathway may contribute to the organization of diverse tissues, both normal and pathological. Although tubulin autoregulation appears functional across diverse human cell types^61–63^, different tissues likely require distinct tubulin levels to support specialized functions. Such specialization could be achieved through additional upstream regulators of TTC5 and/or SCAPER and fine-tuning of the tubulin autoregulation pathway. Similarly, tubulin autoregulation may act in pathological conditions, as seen in heart hypertrophy, where it seems to rewrite the tubulin isotype code^64^. The development of in vivo models and reporters of tubulin autoregulation will be key to charting this pathway’s activity across biological contexts.

Mutations in TTC5 and SCAPER have been linked to a range of human diseases, including hereditary dystonia, neurodevelopmental syndromes, and various cancers^24,65,66^, though the molecular underpinnings remain unknown. Our work raises the possibility that these phenotypes stem from disrupted microtubule behavior and function due to impaired tubulin quantity control—an idea that warrants further mechanistic study.

Given the importance of microtubule dynamics in cancer, and the observation that tubulins are often upregulated in tumors^67^, targeting TTC5 or the broader autoregulation machinery may represent a novel therapeutic strategy. Supporting this hypothesis, our data reveal increased sensitivity of tubulin autoregulation-deficient spheroids to microtubule-stabilizing poisons such as paclitaxel. Future work is required to profile the druggability of the tubulin autoregulation pathway and evaluate its potential for tumor-selective intervention.

Together, our findings place tubulin autoregulation at the core of microtubule regulation and multicellular integrity, offering insight into how cells orchestrate cytoskeletal dynamics via regulated protein biosynthesis. By uncovering a physiological role for this long-elusive feedback loop, we lay the groundwork for deeper exploration of protein quantity control and its implications in health and disease.

## Methods

### Cell culture and cell lines generation

Flp-In T-REx HeLa cells (Thermo Fisher Scientific, #R71407) were maintained in DMEM with GlutaMAX (Thermo Fisher Scientific, #10566016) supplemented with 10% Fetal Bovine Serum (FBS, Pan Biotech, #P30-3306) and 1× Penicillin-Streptomycin (Thermo Fisher Scientific, #15140122) at 37 °C in humidified conditions with 5% CO_2_.

Stable cell lines expressing TTC5 WT and autoregulation-incompetent mutants were generated previously^19^. E-Cadherin (Addgene, #28009) and EB3-EGFP (kindly provided by Prof. Charlotte Aumeier, University of Geneva) were subcloned into a pcDNA5/FRT/TO-EGFP vector using Gibson Assembly. Flp-In T-REx HeLa parental and TTC5 KO cells were co-transfected with pOG44 (Flp recombinase) and the plasmid of interest at a 1:1 ratio using Lipofectamine 3000 (Thermo Fisher Scientific, #L3000015), following the manufacturer’s protocol (#K650001). The following day, cells were selected with 0.2 mg/mL Hygromycin B (Corning, #30-240-CR) and 10 μg/mL Blasticidine S (Thermo Fisher Scientific, #R21001) for 10–15 days. Transgene expression was induced with 200 ng/mL Doxycycline (Sigma-Aldrich, #D9891-1G) for a minimum of 24 hours. All cell lines were routinely checked for mycoplasma contamination.

For transient expression of EB3-EGFP, ~400,000 cells (parental and TTC5 KO) were seeded in 6-well plates and transfected with 0.5 μg of EB3-EGFP plasmid using 1:5 ratio of DNA:Lipofectamine 3000. After 24 hours, cells were trypsinized and grown in spheroids for 5 days.

### 3D cell culture (human spheroids)

Spheroids were generated by seeding 1000 Flp-In T-REx HeLa or HEK293 cells per well into Nunclon Sphera 96-well U-shaped-bottom plates (Thermo Fisher Scientific #174925) in complete growth medium containing 200 ng/mL Doxycycline. Plates were centrifuged at 290 × *g* for 5 minutes at room-temperature and incubated at 37 °C in a humidified 5% CO_2_ atmosphere. Spheroids were maintained for up to 10 days in culture, with two media changes performed between days 5 and 10.

For rescue experiments, Matrigel (Corning, #356231, Lot n° 2262003) was added to the medium at 1 mg/mL on either day 5 or day 7, with analysis of spheroid growth and viability at day 10.

For drug treatment assays, microtubule-targeting agents and Cdk1 inhibitor were diluted in complete medium and added at day 5 or day 7. Final concentrations used were: nocodazole 25 nM or 1-2 μM (nocodazole shock), Paclitaxel 20, 40 or 80 nM, T007-1 400 nM, Colchicine 5 μM, and Ro-3306 5 μM at the indicated treatment duration.

### mRNA quantification by RT-qPCR

Parental, TTC5 KO, and indicated rescue cell lines were cultured as spheroids with 200 ng/mL Doxycycline for 10 days. For the tubulin autoregulation assay, spheroids were treated with 5 μM colchicine (Sigma-Aldrich, #PRH1764) or 0.01% DMSO (Sigma-Aldrich, #D2438) for 6 hours. Spheroids were dissociated with Accutase (Thermo Fisher Scientific, #00-4555-56), and RNA extracted using the NucleoSpin RNA Mini Kit (Macherey-Nagel, #740955). DNA synthesis was performed from 1 μg RNA using the SensiFAST cDNA Synthesis Kit (Bioline, #BIO-65054), as per the manufacturer’s instructions. qPCR was carried out by adding 10 ng of cDNA, 2x PowerUp SYBR Green Master Mix (Thermo Fisher Scientific, #A25777), and the indicated primers in Table 1 on a Bio-Rad thermocycler.

**Table 1:** Oligonucleotide primers used for RT-qPCR analysis

PGK1	mRNA-forward	5′ - CCGCTTTCATGTGGAGGAAGAAG - 3′
	mRNA-reverse	5′ - CTCTGTGAGCAGTGCCAAAAGC - 3′
EEF1A1	mRNA-forward	5′ - GATGGCAATGCCAGTGGAACCA - 3′
	mRNA-reverse	5′ - GAGAACACCAGTCTCCACTCGG - 3′
TUBA4A	mRNA-forward	5′ - GGCAAGGAGATCATTGACCCAG - 3′
	mRNA-reverse	5′ - CATCAGGAGTGAGGTGAAGCCA - 3′
TUBA1B	mRNA-forward	5′ - GAGGAGATGACTCCTTCAACACC - 3′
	mRNA-reverse	5′ - TGATGAGCTGCTCAGGGTGGAA - 3′
TUBA3C	mRNA-forward	5′ - GGAGCTCAACATGCGTGAGTG - 3′
	mRNA-reverse	5′ - CTTGCCAGCTCCAGTCTCAC - 3′
TUBB6	mRNA-forward	5′ - CGTCCGCAGAGCCAGTTC - 3′
	mRNA-reverse	5′ - ATCACTTCCCAAAACTTGGTGC - 3′
TUBB	mRNA-forward	5′ - GAAGCCACAGGTGGCAAATA - 3′
	mRNA-reverse	5′ - CGTACCACATCCAGGACAGA - 3′
TUBB4A	mRNA-forward	5′ - AACATGGCATCGACCCCACA - 3′
	mRNA-reverse	5′ - CTGGGGACATAATTTCCTCCTGT - 3′

Relative expression was analyzed via the ddCt method^68^, normalized to reference housekeeping genes (PGK1 and EEF1A1), and showed relative to DMSO or parental controls. Experiments include a minimum of three biological replicates. Data analysis and plotting were conducted using R/RStudio (v2022.12.0 + 353).

### Western blotting and quantification

Spheroids were dissociated into single cells with Accutase and lysed in RIPA buffer (50 mM Tris-HCl, pH 8.0, 150 mM NaCl, 1% Triton X-100, 0.5% sodium deoxycholate, 0.1% SDS) supplemented with protease (1:100, Halt™ Thermo Fisher Scientific, #78429) and phosphatase inhibitors (1:25, PhosSTOP, Roche, #4906845001).

Protein lysates (3–10 μg) were separated via SDS-PAGE on 4–12% Tris-Glycine pre-cast gels (Thermo Fisher Scientific, #XP04125BOX) or 10% homemade Tris-Glycine gels, and transferred to 0.2 μm nitrocellulose membranes (Amersham, Cytiva). After Ponceau S staining (Roth, #5938.1), membranes were blocked in 5% non-fat dry milk prepared in PBS containing 0.2% Tween-20 (Santa Cruz, #sc-29113). Primary antibodies: anti-α-tubulin (1:5000, Proteintech, #11224-1-AP, Lot n° 0013194), anti-β-tubulin (1:1500, ABCD antibodies, #AA344), anti-acetylated tubulin (Lys40) (1:1500, Proteintech, #66200-1-Ig, Clone No.7E5H8), and anti-GAPDH (1:10000, Cell Signaling Technology, #2118S, Lot n° 10) were incubated overnight at 4 °C. Detection was carried out using IRDye-conjugated secondary antibodies LI-COR 550 (1:10,000, Azure Biosystems, #AC2159), LI-COR 800 or 680 (1:5000, Thermo Fisher Scientific, #A32735; #A32729) and imaging was performed with the Sapphire system (Azure Biosystems).

Band intensities were quantified in Fiji (ImageJ, 1.54f). Briefly, rectangular regions of interest were manually drawn around each band, keeping the area constant throughout all lanes. Upon background subtraction, mean intensity values of α-, β- or acetylated tubulin were normalized to the corresponding GAPDH signal.

### Sample preparation for proteomic analysis

Spheroids from parental and TTC5 KO HeLa cells (10 days in culture) were harvested in PBS. Samples were prepared for liquid chromatography/mass spectrometry (LC/MS) using the phase-transfer surfactant method with minor modifications. First, proteins were extracted and solubilized using buffer containing 12 mM sodium deoxycholate, 12 mM sodium N-dodecanoylsarcosinate, and 100 mM Tris pH 9.0, with EDTA-free Protease Inhibitor Cocktail (Roche). Samples were sonicated for 4 minutes using a Bandelin Sonorex ultrasonic bath (FAUST) with 20-seconds on/20-seconds off cycles. Cell debris was removed after centrifugation at 18,000 × *g* for 20 minutes at 4 °C. Samples were reduced with 10 mM TCEP at 37 °C for 30 minutes and alkylated with 20 mM iodoacetamide in the dark at room temperature for 30 minutes. Alkylation reactions were quenched with 75 mM cysteine at room temperature for 10 minutes. Samples were diluted with 3.1 volumes of 50 mM ammonium bicarbonate. Lysyl endopeptidase and trypsin (Promega) were added at a 50:1 ratio of sample protein: enzyme (w/w) and samples were digested for 16 hours at 37 °C. Afterward, 1.77 volumes of ethyl acetate were added, and samples were acidified with trifluoroacetic acid (TFA), which was added to 0.46% (v/v). Following centrifugation at 12,000 × *g* for 5 minutes at room temperature, samples separated into two phases. The upper organic phase containing sodium deoxycholate was removed, and the lower aqueous phase containing digested tryptic peptides was dried using a centrifugal vacuum concentrator. Digested peptides were dissolved in 300 μL of 0.1% (v/v) TFA in 3% acetonitrile (v/v). Samples were sonicated for 1 minute, centrifuged at 15,000 × *g* for 15 minutes, and desalted using MonoSpin C18 columns (GL Sciences Inc.). Peptides were eluted from C18 columns using 0.1% TFA in 50% acetonitrile and dried in a vacuum concentrator. Tryptic peptides were dissolved in 0.1% (v/v) formic acid in 2% (v/v) acetonitrile for mass spectrometry analysis.

### Mass spectrometry

Samples were measured on an EASY nLC 1000 - Orbitrap Fusion Tribrid mass spectrometer equipped with a nanospray Flex™ ion source (ThermoFisher Scientific). Peptides were separated on a self-packed 1.9-μm particle, 75-μm inner diameter, 15 to 20-cm filling length homemade C18 column. A flow rate of 300 nL/min was used with a 114-min gradient (2–25% solvent B in 100 minutes, 25–45% solvent B in 7 minutes, 45–75% solvent B in 7 minutes). The gradient was followed with two rounds of washing steps; in each step, the gradient switched to 98% solvent B in 1 minute and remained for 2 times, then switched to 2% solvent B in 1 minute and remained for 2 minutes. After the second round of washing, 2% solvent B was reached in 1 minute and remained for 14 minutes for system equilibration. Solvent A was 0.1% (v/v) formic acid in LC/MS grade water and solvent B was 0.1% (v/v) formic acid in 100% (v/v) acetonitrile. The ion source settings from Tune were used for the mass spectrometer ion source properties.

For data-independent acquisition (DIA), data were acquired with 1 full MS and 38 overlapping isolation windows constructed covering the precursor mass range of 350–1200 *m/z*. For full mass spectrometry, Orbitrap resolution was set to 120,000. AGC target was set to 250% with a maximum injection time (IT) of 60 milliseconds. DIA segments were acquired at resolution of 30,000. AGC target was set to 2000% with a dynamic maximum IT. HCD fragmentation was set to a normalized collision energy of 27%, covering a fragment ion mass range of 200–2000 *m/z*.

For protein identification and quantification, raw files were analyzed using the directDIA+ (Deep) workflow in Spectronaut 20 software (release 20.3.251119, Biognosys) with default settings unless otherwise stated. Briefly, identification was performed using the UniProt reference proteome for human (release 2026_01 - 20416 entries). Digestion enzyme specificity was set to Trypsin/P, considering peptides between 7 and 52 residues with a maximum of two missed cleavages. Modifications included carbamidomethylation of cysteine as a fixed modification, and oxidation of methionine and acetyl (protein N-terminus) as variable modifications (maximum of 5). False discovery rate (FDR) control was set to 1% at the PSM, peptide, and protein group levels. MS1 and MS2 mass tolerances were set dynamically, with the ideal mass tolerance determined after a first-pass calibration (±40 ppm). Between 3 and 6 best fragment ions were used per peptide. Single-hit proteins were defined by stripped sequence and were excluded. Identified precursor quantification was based on peak area at the MS2 level without imputation of missing values. Protein group quantity is the average of the three best peptides. Cross-run normalization was carried out using normalization based on a retention-dependent local regression model^69^. Protein inference was computed with the parsimony approach using the IDPicker algorithm^70^.

For the two-group comparison, differential abundance testing was performed with an unpaired *t*-test. *Q*-values were the multiple testing corrected *p*-values. Data processing and visualization were done in R/Rstudio (v2022.12.0 + 353). Processed data are provided as Supplementary Data 1 and protein intensity values for each protein across all samples are provided as Supplementary Data 2. Mass spectrometry raw data have been uploaded to the ProteomeXchange Consortium using PRIDE with the dataset identifier PXD07680.

### Microscopy-based assays

#### Scanning confocal microscopy (EB3 comet tracking and counting)

Five- or seven-day-old spheroids transiently or stably expressing EB3-EGFP were transferred into µ-Slide 15 Well 3D dishes (iBidi, #81506) and imaged in phenol-red-free Leibovitz’s L-15 medium (Thermo Fisher Scientific, #21083027) supplemented with 10% FBS or regular culture medium with 10 mM HEPES (Pan Biotech, #P05-01100). Imaging was performed on a temperature-controlled Zeiss LSM980 confocal microscope fitted on an inverted Axio Observer 7 Microscope with an Airyscan detector optimized for a Plan-ApoChromat 63×/1.40 NA oil objective (0.085 μm/pixel). Acquisition optics were composed of a laser line 488 nm with 4-5× digital zooming. Time-lapse imaging captured one plane every 1.5 seconds for 1 minute. ZEN (blue edition, 3.3.89.0008) software was used for image acquisition and Airyscan processing.

EB3 comet dynamics were analyzed using the multiple-particle tracking MATLAB software u-track^71^. Custom parameters were set for: (1) comet detection: difference of Gaussians filter parameters 1–4 pixels; watershed segmentation 5 standard deviations; (2) tracking: maximum gap to close 3 frames; minimum length of track segments 4 frames; search radius 2–8 pixels; maximum forward/backward angles 40°/10°; break non-linear tracks; and (3) microtubule dynamics classification. Due to the low frequency of shrinkage events, we focused our analysis on microtubule growth length and speed, tracked over a minimum of 4.5 seconds (four to six consecutive frames). Cytoplasmic EB3-EGFP intensities, measured as the mean intensity on a region of interest in the cytoplasm of each analyzed cell, were used to exclude outliers (average intensity ± standard deviation, grouped per experiment). Multiple cells were analyzed from three-five spheroids per biological replicate. Due to imaging constraints, these measurements were performed at the spheroid periphery.

Detection of EB3 comet density per unit area in DMSO- and nocodazole-treated spheroids was performed using ImageJ. Images were processed by applying a Gaussian blur filter (radius = 2 pixels) prior to manual identification and counting of EB3 comets. Comet density was calculated as the number of comets per defined area.

#### Scanning confocal microscopy (fixed spheroids)

After processing spheroids for immunofluorescence (see section below), imaging was performed on the same confocal setup. Excitation wavelengths were composed of 405 nm, 488 nm, 561 nm, and 647 nm laser lines, with 1.5–2× digital zooming and a z-step of 0.2 μm. Due to the large spheroid volume and constraints in imaging depth, acquisition was limited to the peripheral regions of the spheroids. All images show max-intensity projections (representation only). Multiple cells were analyzed from two to four spheroids per biological replicate. Based on DAPI staining, the characteristic chromatin compaction and the presence of the mitotic spindle (when available), dead and mitotic cells were excluded from the quantification.

For analysis, sum-projection images were obtained in Fiji. Cell cortex was manually defined (using the segmented line tool, 20 pixels) with Phalloidin/actin staining as reference. Acetylated-tubulin and α-tubulin fluorescence mean intensities were measured along the cortical region (using actin localization as a reference) to avoid confounding effects arising from differences in nuclear-to-cytoplasmic ratios between parental and TTC5 KO cells, which alter the relative area occupied by the microtubule network. Results are presented as a ratio of acetylated-tubulin over total α-tubulin. For aspect ratio measurements (defined by the ratio of the cell width to its height), the polygon tool was used to mark the cell cortex, using phalloidin/actin staining as reference.

Cortical and cytoplasmic intensities were extracted via line scans (20–30 pixels) placed from the center of individual cells to the cortex, using actin as a reference. This metric directly captures changes in protein redistribution from the cortex to the cytoplasm. Cortex-to-cytoplasm ratio of adhesion proteins was defined as *p/m*, where *p* is the mean intensity within ± 1 μm from peak intensity (cell cortex) and *m* is the mean intensity 1–2 μm from *p* (into the cytoplasm).

#### Widefield microscopy (spheroid growth and drug response)

Spheroid growth was monitored from day 0 to day 10 on a Nikon Eclipse Ti2-E inverted microscope (Nikon), equipped with a Kinetix sCMOS camera (Photometrics), Spectrax Chroma light engine for fluorescence illumination (Lumencor), and an incubation chamber at 37 °C with controlled humidity and 5% CO_2_ (OkoLab). Brightfield multiple stage positions were acquired using NIS Elements (Nikon) equipped with a Plan Apochromat Lambda 10× objective (NA 0.45, Nikon) sampling 0.64 μm/pixel. Single plain images or maximum intensity projections were prepared in Fiji and spheroid area analyzed using a manual or automatic pipeline that runs trainable Weka Segmentation plugin (version 3.3.2) to identify core, corona, and background classes. Binary images were generated from the probability maps using Otsu threshold (auto adjustment was used as default, but manual adjustments were required in some instances), and areas (core, corona) measured in microns.

#### Widefield microscopy (cell viability assay)

Nine-day-old spheroids stained with Hoechst and PI to visualize all nuclei and dead cells, respectively, were imaged in steps of 5 μm, every 20 minutes for 10 hours. Images were denoised (NIS Elements), and maximum intensity projections of representative examples were prepared in Fiji and exported as still images. The probability of survival at the spheroid corona (%) was quantified by tracking individual Hoechst-positive cells located at the corona at the onset of imaging and following their survival over time based on PI signal. Cells that moved out of the focal plane during acquisition were excluded from the analysis.

#### High-throughput live-cell imaging (cell viability assay)

Ten-day-old spheroids were stained with Hoechst and PI and imaged using an ImageXpress Micro Confocal automated microscope (Molecular Devices™) equipped with a Plan Apochromat Lambda 10x objective (0.45 NA, Nikon). Image segmentation was performed using a custom module editor, MetaXpress from Molecular Devices. Briefly, single nuclei were masked and segmented using Hoechst signal. The percentage of dead cells was calculated by dividing the number of PI-positive cells by the total number of segmented nuclei.

#### High-throughput live-cell imaging (spheroid migration assay)

Seven-day-old spheroids were imaged in a 384-well plate coated with Matrigel for 48 h (see “Spheroid migration assay” section) using a IXM confocal automatic microscope with the same specs as indicated above. Spheroid and migration areas were segmented independently and quantified with MetaXpress Custom Module editor software based on brightfield imaged. Only cells migrating from the spheroid (3D) onto the Matrigel-coated surface (2D) were included in the analysis. The migration area was defined as the difference between the total area at timepoint 0, 24 and 48 hours and the spheroid area at the time of transfer (*t* = 0).

### Immunofluorescence

Spheroids (7 or 10 days) were fixed in 4% paraformaldehyde (PFA, Electron Microscopy Sciences #15713) in PBS for 30 minutes at 37 °C. Permeabilization was performed using PBS-0.2% Triton (Sigma-Aldrich X-100, #9036-19-5) during 30 minutes at room-temperature. Spheroids were washed 3× with PBS and incubated for 2 hours at room-temperature with blocking solution: 2% BSA (PAN Biotech, #P06-1391500, Lot n° H210207) diluted in PBS with 0.02% Tween 20 (PBS-T). Primary antibodies: anti-acetylated tubulin (1:200, Sigma-Aldrich, #T7451, Lot n° 0000312701), anti-α-tubulin (1:200, Proteintech, #11224-1-AP, Lot n° 0013194), anti-N-Cadherin (1:200, Proteintech, #22018-1-AP, Lot n° 00111704), anti-Fibronectin (1:1000, clone 1801, kind gift from Prof. Bernhard Wehrle-Haller, University of Geneva), anti-Integrin α5 (1:10, AA430-M2a, kind gift from Prof. Bernhard Wehrle-Haller, University of Geneva) and anti-cleaved Caspase3 (1:200, Proteintech, #68773-1, Lot n° 10041389) were diluted in blocking solution and incubated overnight at 4 °C. Subsequently, spheroids were washed 3x with PBS-T and incubated for 2 hours at room temperature with the corresponding secondary antibody: Alexa 488 (1:500, Thermo Fisher Scientific, #A32731, Lot n° WC318798) and 555 (1:500, Thermo Fisher Scientific, #A32727, Lot n° WA316324), together with DAPI (1:5000, 5 mg/mL, Thermo Fisher Scientific, #D1306) and phalloiding-647 (1:250, Thermo Fisher Scientific, #A22287, Lot n° 1583100). After three washes with PBS-T, spheroids were kept in PBS at 4 °C until imaging.

### Cell viability measurements

Ten-day-old spheroids were stained with PI (1:2000, 1 mg/mL, Sigma-Aldrich, #81845) and Hoechst 33342 (1:2000, 10 mg/mL, Molecular Probes, #H-3570) for 2 hours before imaging. Spheroids were processed for widefield or high-throughput microscopy as indicated in the “Microscopy-based assays” section.

For the ATP-based viability assay, spheroids at days 7 and 10 were incubated with CellTiter-Glo 3D reagent (Promega, #G9681) for 30 minutes, following the manufacturer’s protocol. Luminescence readout was measured on a Cytation 3 imaging reader (Gen5 3.14).

### Cell cycle profiles

Spheroids (10-day) were dissociated with Accutase for 10–15 minutes at 37 °C. After centrifugation (300 × *g* for 5 minutes at room-temperature), cells were fixed in 90% cold methanol (−20 °C, overnight). Following washes with PBS, DNA was stained with PI (1:60, 1 mg/mL) together with RNase treatment (1:150, Roche, #11119915001). Cell cycle profiles, based on PI staining, were acquired on a Gallios cytometer (Model 2L/8C, Beckman Coulter), and analyzed using Kaluza flow cytometry software (Beckman Coulter) and FCS Express 7. Due to limitations of the archival flow cytometry data files, only events retained after the initial scatter-based gate were available for re-analysis. The gating strategy is shown in Fig. S5a–e and the full dataset is available in the Source Data file.

### Spheroid migration assay

Seven-day-old parental, TTC5 KO, and TTC5^WT^ spheroids were manually transferred to a 384-well plate (Corning, #353962) previously coated with 125 μg/mL Matrigel (Corning, #356231, 2 hours at room temperature) following an established protocol^72^. Cell migration was recorded for 2 days (time points 0, 24, and 48 hours) using an IXM confocal automatic microscope (see “Microscopy-based assays” section for imaging and quantification details).

### Statistical analysis

For all datasets, the number of independent biological replicates (N), the number of cells or spheroids analyzed (n), and statistical tests with the corresponding *p* values are detailed in the figures or figure legends. No statistical method was used to predetermine the sample size. The experiments were not randomized, and the researchers were not blinded to allocation during experiments and outcome assessment. For experiments with three or more biological replicates, statistical analyzes were performed using the N number in R and GraphPad Prism 8, which reports exact *p* values up to four decimal places, with smaller values shown as *p* < 0.0001. When applicable (n > 10), the D’Agostinho-Pearson omnibus normality test was used to determine if the data followed a normal distribution. For datasets with *n* < 10 (e.g., RT-qPCR and WB analyzes), normality was determined using the Shapiro-Wilk test. When *α* = 0.05 for most of the compared conditions, statistical significance between conditions was determined by Student’s *t*-test or one-way ANOVA followed by Holm-Šidák multiple comparisons test. When *α* < 0.05, non-parametric tests were used, including the Kolmogorov-Smirnov test or the Kruskal-Wallis test followed by Dunn’s multiple comparisons correction. Data are presented as mean ± Standard deviation (SD) or Standard Error of the Mean (SEM); or median ± Interquartile Range (IQR), depending on the normality of the data distribution. Outliers were identified and removed using the ROUT method (*Q* = 1%). Datasets used for analysis and plotting are shown in the Source Data file. For all graphs, statistical significance is annotated as follows: ^∗∗∗∗^*p* < 0.0001, ^∗∗∗^p < 0.001, ^∗∗^*p* < 0.01, ^∗^*p* < 0.05, and ns - not significant.

### Reporting summary

Further information on research design is available in the Nature Portfolio Reporting Summary linked to this article.

## Supplementary information

Supplementary Information

Description of Additional Supplementary Files

Supplementary Data 1

Supplementary Data 2

Reporting Summary

Transparent Peer Review file

## Source data

Source Data

## Data Availability

All the data are available in the Source Data file and on the publicly available PRIDE repository with the dataset identifier PXD07680. Source data are provided with this paper.

## References

[1] Muroyama, A. & Lechler, T. Microtubule organization, dynamics and functions in differentiated cells. *Development* **144**, 3012–3021 (2017). 28851722 10.1242/dev.153171 PMC5611961

[2] Prosser, S. L. & Pelletier, L. Mitotic spindle assembly in animal cells: a fine balancing act. *Nat. Rev. Mol. Cell Biol.* **18**, 187–201 (2017). 28174430 10.1038/nrm.2016.162

[3] Wade, R. H. & Hyman, A. A. Microtubule structure and dynamics. *Curr. Opin. Cell Biol.* **9**, 12–17 (1997). 9013674 10.1016/s0955-0674(97)80146-9

[4] Mitchison, T. & Kirschner, M. Dynamic instability of microtubule growth. *Nature* **312**, 237–242 (1984). 6504138 10.1038/312237a0

[5] Bodakuntla, S., Jijumon, A. S., Villablanca, C., Gonzalez-Billault, C. & Janke, C. Microtubule-associated proteins: structuring the cytoskeleton. *Trends Cell Biol.* **29**, 804–819 (2019). 31416684 10.1016/j.tcb.2019.07.004

[6] Goodson, H. V. & Jonasson, E. M. Microtubules and microtubule-associated proteins. *Cold Spring Harb. Perspect. Biol.* **10**, a022608 (2018). 10.1101/cshperspect.a022608 PMC5983186 29858272

[7] Gudimchuk, N. B. & McIntosh, J. R. Regulation of microtubule dynamics, mechanics and function through the growing tip. *Nat. Rev. Mol. Cell Biol.* **22**, 777–795 (2021). 34408299 10.1038/s41580-021-00399-x

[8] Zheng, Y., Wong, M. L., Alberts, B. & Mitchison, T. Nucleation of microtubule assembly by a gamma-tubulin-containing ring complex. *Nature* **378**, 578–583 (1995). 8524390 10.1038/378578a0

[9] Chretien, D., Fuller, S. D. & Karsenti, E. Structure of growing microtubule ends: two-dimensional sheets close into tubes at variable rates. *J. Cell Biol.* **129**, 1311–1328 (1995). 7775577 10.1083/jcb.129.5.1311 PMC2120473

[10] Bieling, P. et al. Reconstitution of a microtubule plus-end tracking system in vitro. *Nature* **450**, 1100–1105 (2007). 18059460 10.1038/nature06386

[11] Hernandez-Vega, A. et al. Local nucleation of microtubule bundles through tubulin concentration into a condensed tau phase. *Cell Rep.* **20**, 2304–2312 (2017). 28877466 10.1016/j.celrep.2017.08.042 PMC5828996

[12] Walker, R. A. et al. Dynamic instability of individual microtubules analyzed by video light microscopy: rate constants and transition frequencies. *J. Cell Biol.* **107**, 1437–1448 (1988). 3170635 10.1083/jcb.107.4.1437 PMC2115242

[13] Ben-Ze’ev, A., Farmer, S. R. & Penman, S. Mechanisms of regulating tubulin synthesis in cultured mammalian cells. *Cell* **17**, 319–325 (1979). 455467 10.1016/0092-8674(79)90157-0

[14] Cleveland, D. W., Lopata, M. A., Sherline, P. & Kirschner, M. W. Unpolymerized tubulin modulates the level of tubulin mRNAs. *Cell* **25**, 537–546 (1981). 6116546 10.1016/0092-8674(81)90072-6

[15] Yen, T. J., Machlin, P. S. & Cleveland, D. W. Autoregulated instability of beta-tubulin mRNAs by recognition of the nascent amino terminus of beta-tubulin. *Nature* **334**, 580–585 (1988). 3405308 10.1038/334580a0

[16] Cleveland, D. W. Autoregulated instability of tubulin mRNAs: a novel eukaryotic regulatory mechanism. *Trends Biochem Sci.* **13**, 339–343 (1988). 3072712 10.1016/0968-0004(88)90103-x

[17] Bachurski, C. J., Theodorakis, N. G., Coulson, R. M. & Cleveland, D. W. An amino-terminal tetrapeptide specifies cotranslational degradation of beta-tubulin but not alpha-tubulin mRNAs. *Mol. Cell Biol.* **14**, 4076–4086 (1994). 8196646 10.1128/mcb.14.6.4076-4086.1994 PMC358773

[18] Yen, T. J., Gay, D. A., Pachter, J. S. & Cleveland, D. W. Autoregulated changes in stability of polyribosome-bound beta-tubulin mRNAs are specified by the first 13 translated nucleotides. *Mol. Cell Biol.* **8**, 1224–1235 (1988). 2835666 10.1128/mcb.8.3.1224-1235.1988 PMC363267

[19] Lin, Z. et al. TTC5 mediates autoregulation of tubulin via mRNA degradation. *Science* **367**, 100–104 (2020). 31727855 10.1126/science.aaz4352 PMC6942541

[20] Hopfler, M. et al. Mechanism of ribosome-associated mRNA degradation during tubulin autoregulation. *Mol. Cell* **83**, 2290–2302.e2213 (2023). 37295431 10.1016/j.molcel.2023.05.020 PMC10403363

[21] Batiuk, A. et al. Soluble alphabeta-tubulins reversibly sequester TTC5 to regulate tubulin mRNA decay. *Nat. Commun.* **15**, 9963 (2024). 39551769 10.1038/s41467-024-54036-0 PMC11570694

[22] Cleveland, D. W., Pittenger, M. F. & Feramisco, J. R. Elevation of tubulin levels by microinjection suppresses new tubulin synthesis. *Nature* **305**, 738–740 (1983). 6633643 10.1038/305738a0

[23] Tate, J. G. et al. COSMIC: the catalogue of somatic mutations in cancer. *Nucleic Acids Res.* **47**, D941–D947 (2019). 30371878 10.1093/nar/gky1015 PMC6323903

[24] Musante, L. et al. TTC5 syndrome: clinical and molecular spectrum of a severe and recognizable condition. *Am. J. Med. Genet. A* **188**, 2652–2665 (2022). 35670379 10.1002/ajmg.a.62852 PMC9541101

[25] Rasheed, A. et al. Bi-allelic TTC5 variants cause delayed developmental milestones and intellectual disability. *J. Med. Genet.* **58**, 237–246 (2021). 32439809 10.1136/jmedgenet-2020-106849 PMC9648057

[26] Tatour, Y. et al. Mutations in SCAPER cause autosomal recessive retinitis pigmentosa with intellectual disability. *J. Med. Genet.* **54**, 698–704 (2017). 28794130 10.1136/jmedgenet-2017-104632

[27] Wormser, O. et al. SCAPER localizes to primary cilia and its mutation affects cilia length, causing Bardet-Biedl syndrome. *Eur. J. Hum. Genet*. **27**, 928–940 (2019). 30723319 10.1038/s41431-019-0347-z PMC6777442

[28] Gasic, I., Boswell, S. A. & Mitchison, T. J. Tubulin mRNA stability is sensitive to change in microtubule dynamics caused by multiple physiological and toxic cues. *PLoS Biol.* **17**, e3000225 (2019). 30964857 10.1371/journal.pbio.3000225 PMC6474637

[29] Basu, S. et al. A role for tubulin in cellular quality control and proteostasis. Preprint at *bioRxiv* https://www.biorxiv.org/content/10.64898/2026.04.06.716648v1 (2026).

[30] Mooney, D. J., Hansen, L. K., Langer, R., Vacanti, J. P. & Ingber, D. E. Extracellular matrix controls tubulin monomer levels in hepatocytes by regulating protein turnover. *Mol. Biol. Cell* **5**, 1281–1288 (1994). 7696710 10.1091/mbc.5.12.1281 PMC301157

[31] Pampaloni, F., Reynaud, E. G. & Stelzer, E. H. The third dimension bridges the gap between cell culture and live tissue. *Nat. Rev. Mol. Cell Biol.* **8**, 839–845 (2007). 17684528 10.1038/nrm2236

[32] Baker, B. M. & Chen, C. S. Deconstructing the third dimension: how 3D culture microenvironments alter cellular cues. *J. Cell Sci.* **125**, 3015–3024 (2012). 22797912 10.1242/jcs.079509 PMC3434846

[33] Cacciamali, A., Villa, R. & Dotti, S. 3D cell cultures: evolution of an ancient tool for new applications. *Front. Physiol.* **13**, 836480 (2022). 35936888 10.3389/fphys.2022.836480 PMC9353320

[34] Mueller-Klieser, W. Three-dimensional cell cultures: from molecular mechanisms to clinical applications. *Am. J. Physiol.* **273**, C1109–C1123 (1997). 9357753 10.1152/ajpcell.1997.273.4.C1109

[35] von Glasenapp, V. et al. Spatio-temporal control of mitosis using light via a Plk1 inhibitor caged for activity and cellular permeability. *Nat. Commun.* **16**, 1599 (2025). 39971898 10.1038/s41467-025-56746-5 PMC11840123

[36] Fennema, E., Rivron, N., Rouwkema, J., van Blitterswijk, C. & de Boer, J. Spheroid culture as a tool for creating 3D complex tissues. *Trends Biotechnol.* **31**, 108–115 (2013). 23336996 10.1016/j.tibtech.2012.12.003

[37] Browning, A. P. et al. Quantitative analysis of tumour spheroid structure. *eLife* **10**, e73020 (2021). 34842141 10.7554/eLife.73020 PMC8741212

[38] Spoerri, L., Beaumont, K. A., Anfosso, A. & Haass, N. K. Real-time cell cycle imaging in a 3D cell culture model of melanoma. *Methods Mol. Biol.* **1612**, 401–416 (2017). 28634959 10.1007/978-1-4939-7021-6_29

[39] Smyrek, I. et al. E-cadherin, actin, microtubules and FAK dominate different spheroid formation phases and important elements of tissue integrity. *Biol. Open* **8**, bio037051 (2019). 30578251 10.1242/bio.037051 PMC6361217

[40] Lin, R. Z. & Chang, H. Y. Recent advances in three-dimensional multicellular spheroid culture for biomedical research. *Biotechnol. J.* **3**, 1172–1184 (2008). 18566957 10.1002/biot.200700228

[41] Piperno, G., LeDizet, M. & Chang, X. J. Microtubules containing acetylated alpha-tubulin in mammalian cells in culture. *J. Cell Biol.* **104**, 289–302 (1987). 2879846 10.1083/jcb.104.2.289 PMC2114420

[42] Evan, G. I., Brown, L., Whyte, M. & Harrington, E. Apoptosis and the cell cycle. *Curr. Opin. Cell Biol.* **7**, 825–834 (1995). 8608013 10.1016/0955-0674(95)80066-2

[43] Gumbiner, B. M. Cell adhesion: the molecular basis of tissue architecture and morphogenesis. *Cell* **84**, 345–357 (1996). 8608588 10.1016/s0092-8674(00)81279-9

[44] Akhmanova, A., Stehbens, S. J. & Yap, A. S. Touch, grasp, deliver and control: functional cross-talk between microtubules and cell adhesions. *Traffic* **10**, 268–274 (2009). 19175539 10.1111/j.1600-0854.2008.00869.x

[45] Yang, J. et al. Covalent modification of Cys-239 in beta-tubulin by small molecules as a strategy to promote tubulin heterodimer degradation. *J. Biol. Chem.* **294**, 8161–8170 (2019). 30940730 10.1074/jbc.RA118.006325 PMC6527155

[46] Gasic, I. Regulation of tubulin gene expression: from isotype identity to functional specialization. *Front. Cell Dev. Biol.* **10**, 898076 (2022). 35721507 10.3389/fcell.2022.898076 PMC9204600

[47] Gay, D. A., Sisodia, S. S. & Cleveland, D. W. Autoregulatory control of beta-tubulin mRNA stability is linked to translation elongation. *Proc. Natl. Acad. Sci. USA*. **86**, 5763–5767 (1989). 2762294 10.1073/pnas.86.15.5763 PMC297710

[48] Preitner, N. et al. APC is an RNA-binding protein, and its interactome provides a link to neural development and microtubule assembly. *Cell* **158**, 368–382 (2014). 25036633 10.1016/j.cell.2014.05.042 PMC4133101

[49] Mourao, M., Schnell, S. & Shaw, S. L. Macroscopic simulations of microtubule dynamics predict two steady-state processes governing array morphology. *Comput. Biol. Chem.* **35**, 269–281 (2011). 22000798 10.1016/j.compbiolchem.2011.06.002

[50] Mohapatra, L., Lagny, T. J., Harbage, D., Jelenkovic, P. R. & Kondev, J. The limiting-pool mechanism fails to control the size of multiple organelles. *Cell Syst.* **4**, 559–567.e514 (2017). 28544883 10.1016/j.cels.2017.04.011 PMC5906859

[51] Rank, M., Mitra, A., Reese, L., Diez, S. & Frey, E. Limited resources induce bistability in microtubule length regulation. *Phys. Rev. Lett.* **120**, 148101 (2018). 29694156 10.1103/PhysRevLett.120.148101

[52] Ferreira, J. G., Pereira, A. J., Akhmanova, A. & Maiato, H. Aurora B spatially regulates EB3 phosphorylation to coordinate daughter cell adhesion with cytokinesis. *J. Cell Biol.* **201**, 709–724 (2013). 23712260 10.1083/jcb.201301131 PMC3664705

[53] Yang, C. et al. EB1 and EB3 regulate microtubule minus end organization and Golgi morphology. *J. Cell Biol.* **216**, 3179–3198 (2017). 28814570 10.1083/jcb.201701024 PMC5626540

[54] Komarova, Y. A., Akhmanova, A. S., Kojima, S., Galjart, N. & Borisy, G. G. Cytoplasmic linker proteins promote microtubule rescue in vivo. *J. Cell Biol.* **159**, 589–599 (2002). 12446741 10.1083/jcb.200208058 PMC2173097

[55] Mimori-Kiyosue, Y. et al. CLASP1 and CLASP2 bind to EB1 and regulate microtubule plus-end dynamics at the cell cortex. *J. Cell Biol.* **168**, 141–153 (2005). 15631994 10.1083/jcb.200405094 PMC2171674

[56] Gasic, I. et al. Tubulin resists degradation by cereblon-recruiting PROTACs. *Cells* **9**, 1083 (2020). 32349222 10.3390/cells9051083 PMC7290497

[57] Stehbens, S. J. et al. Dynamic microtubules regulate the local concentration of E-cadherin at cell-cell contacts. *J. Cell Sci.* **119**, 1801–1811 (2006). 16608875 10.1242/jcs.02903

[58] Mary, S. et al. Biogenesis of N-cadherin-dependent cell-cell contacts in living fibroblasts is a microtubule-dependent kinesin-driven mechanism. *Mol. Biol. Cell* **13**, 285–301 (2002). 11809840 10.1091/mbc.01-07-0337 PMC65089

[59] Waterman-Storer, C. M., Salmon, W. C. & Salmon, E. D. Feedback interactions between cell-cell adherens junctions and cytoskeletal dynamics in newt lung epithelial cells. *Mol. Biol. Cell* **11**, 2471–2483 (2000). 10888682 10.1091/mbc.11.7.2471 PMC14933

[60] Kaverina, I., Rottner, K. & Small, J. V. Targeting, capture, and stabilization of microtubules at early focal adhesions. *J. Cell Biol.* **142**, 181–190 (1998). 9660872 10.1083/jcb.142.1.181 PMC2133026

[61] Fellous, A., Ginzburg, I. & Littauer, U. Z. Modulation of tubulin mRNA levels by interferon in human lymphoblastoid cells. *EMBO J.* **1**, 835–839 (1982). 6964957 10.1002/j.1460-2075.1982.tb01256.x PMC553118

[62] Caron, J. M., Jones, A. L. & Kirschner, M. W. Autoregulation of tubulin synthesis in hepatocytes and fibroblasts. *J. Cell Biol.* **101**, 1763–1772 (1985). 3902854 10.1083/jcb.101.5.1763 PMC2113958

[63] Lau, J. T., Pittenger, M. F., Havercroft, J. C. & Cleveland, D. W. Reconstruction of tubulin gene regulation in cultured mammalian cells. *Ann. N. Y Acad. Sci.* **466**, 75–88 (1986). 3460447 10.1111/j.1749-6632.1986.tb38385.x

[64] Phyo, S. A. et al. Transcriptional, post-transcriptional, and post-translational mechanisms rewrite the tubulin code during cardiac hypertrophy and failure. *Front. Cell Dev. Biol.* **10**, 837486 (2022). 35433678 10.3389/fcell.2022.837486 PMC9010559

[65] Fasham, J. et al. Delineating the expanding phenotype associated with SCAPER gene mutation. *Am. J. Med Genet. A* **179**, 1665–1671 (2019). 31192531 10.1002/ajmg.a.61202 PMC6772143

[66] Hussey, S. P. & Fritz-Laylin, L. K. The Missing Link”: The Tubulin Mutation Database Connects Over 1500 Missense mutations with phenotypes across eukaryotes. *Cytoskeleton* **76**, 175–176 (2019). 30907069 10.1002/cm.21517

[67] Parker, A. L., Kavallaris, M. & McCarroll, J. A. Microtubules and their role in cellular stress in cancer. *Front. Oncol.* **4**, 153 (2014). 24995158 10.3389/fonc.2014.00153 PMC4061531

[68] Livak, K. J. & Schmittgen, T. D. Analysis of relative gene expression data using real-time quantitative PCR and the 2(-Delta Delta C(T)) Method. *Methods* **25**, 402–408 (2001). 11846609 10.1006/meth.2001.1262

[69] Callister, S. J. et al. Normalization approaches for removing systematic biases associated with mass spectrometry and label-free proteomics. *J. Proteome Res.* **5**, 277–286 (2006). 16457593 10.1021/pr050300l PMC1992440

[70] Zhang, B., Chambers, M. C. & Tabb, D. L. Proteomic parsimony through bipartite graph analysis improves accuracy and transparency. *J. Proteome Res.* **6**, 3549–3557 (2007). 17676885 10.1021/pr070230d PMC2810678

[71] Applegate, K. T. et al. plusTipTracker: quantitative image analysis software for the measurement of microtubule dynamics. *J. Struct. Biol.* **176**, 168–184 (2011). 21821130 10.1016/j.jsb.2011.07.009 PMC3298692

[72] Vinci, M., Box, C., Zimmermann, M. & Eccles, S. A. Tumor spheroid-based migration assays for evaluation of therapeutic agents. *Methods Mol. Biol.* **986**, 253–266 (2013). 23436417 10.1007/978-1-62703-311-4_16

