# Supplementary material for: Tubulin autoregulation tunes microtubule dynamics to support multicellular architecture and viability
Source: Nat Commun. 2026 Jul 22;17:6813. doi: 10.1038/s41467-026-75341-w (PMC13392243; doi:10.1038/s41467-026-75341-w)
Supplement: Supplementary file 1 — Supplementary Information [file 41467_2026_75341_MOESM1_ESM.docx]

Supplementary Information

Almeida AC et al, 2026

Tubulin autoregulation tunes microtubule dynamics to support multicellular architecture and viability

Supplementary Figure 1

Supplementary Figure 2

Supplementary Figure 3

Supplementary Figure 4

Supplementary Figure 5

Supplementary Figure 6

Supplementary Figure 7

Supplementary Figure 8

Supplementary Figure 9

Supplementary Figure 1


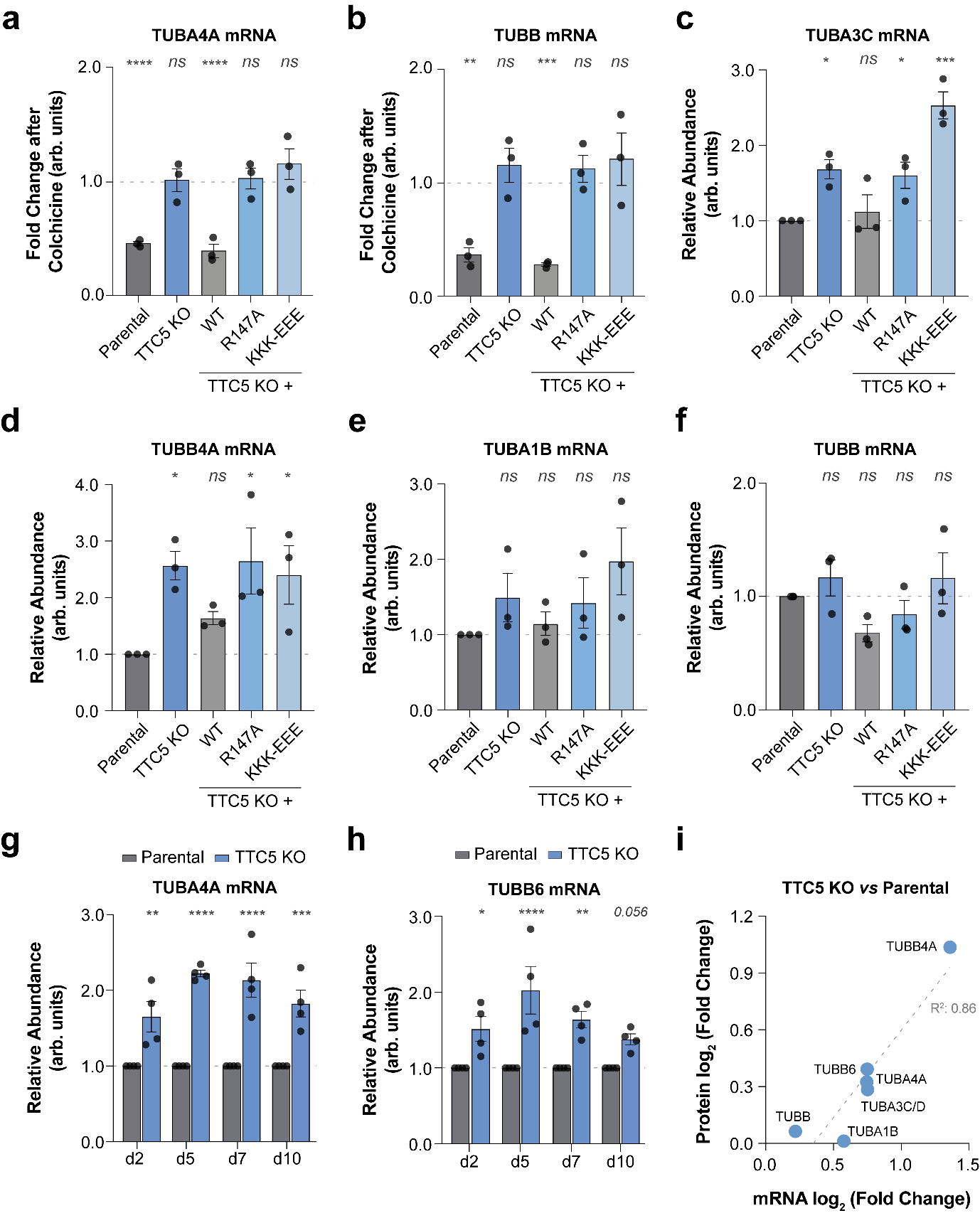


Fig. S1. (related to Fig. 1) Autoregulation assay with HeLa parental, TTC5 KO, and the indicated Flag-TTC5 rescue cell lines, normalized to the DMSO condition. TUBA4A a and TUBB b mRNA levels after 6 hours of colchicine treatment from three independent experiments. Data represent mean ± SEM. *p* values were determined using one-way ANOVA followed by Holm-Šidák’s multiple comparisons test, comparing each cell line to its corresponding DMSO-treated condition. Relative abundance of TUBA3C c, TUBB4A d, TUBA1B e, and TUBB f mRNA in HeLa parental, TTC5 KO, and the indicated Flag-TTC5 cell lines in 10-day-old spheroids, normalized to housekeeping transcripts and parental levels. Data show mean ± SEM from a minimum of three independent experiments. *p* values reflect one-way ANOVA followed by Holm-Šidák’s multiple comparisons test for each of the indicated cell lines compared to the parental line. Relative abundance of TUBA4A g and TUBB6 h transcripts during spheroid growth (days 2, 5, 7, and 10). Graphs represent mean ± SEM from three independent biological replicates (including technical replicates in one). *p* values were determined by one-way ANOVA followed by Holm-Šidák’s multiple comparisons test, comparing each indicated time point to the parental line. i Correlation between tubulin protein levels (MS dataset, log2 (Fold Change)) and transcript levels (RT-qPCR, log2 (Fold Change)) in TTC5 KO spheroids. Simple linear regression is shown (R2: 0.86). *****p<*0.0001, ****p<*0.001, ***p<*0.01, **p<*0.05, ns - not significant. Exact *p* values can be found in the Source Data file.

Supplementary Figure 2


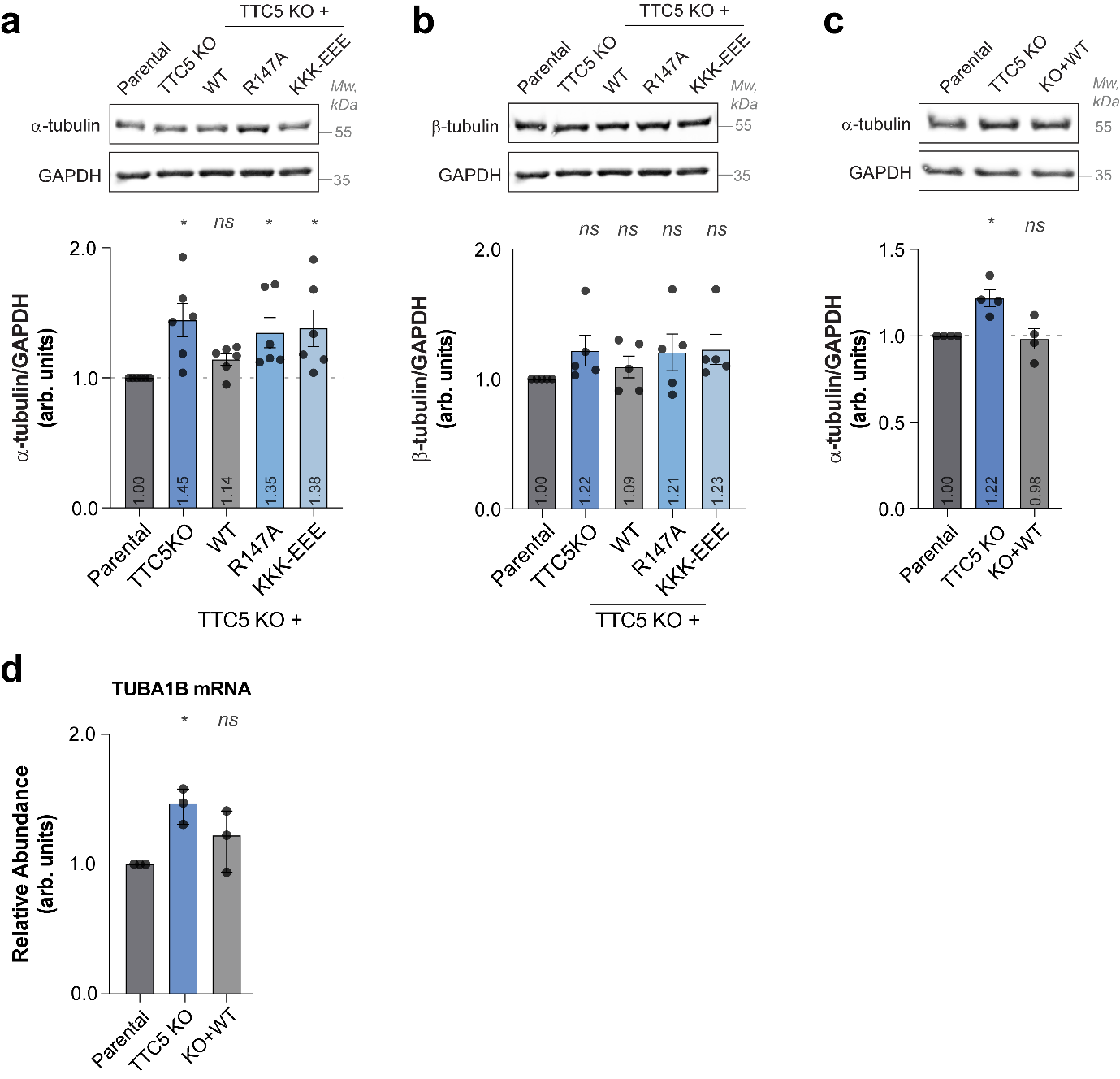


**Fig. S2. (related to Fig. 1)** Western blot analysis of total a-tubulin **a** and b-tubulin **b** protein normalized to the loading control (GAPDH) and parental cell line. Data show mean ± SEM from a minimum of four independent experiments (including technical replicates in one or two). *p* values were determined by one-way ANOVA followed by Holm-Šidák’s multiple comparisons test, comparing each indicated cell line to the parental line. **c** Western blot analysis of total a-tubulin protein levels in HEK293 parental, TTC5 KO, and TTC5WT spheroids. Data are shown as mean ± SEM from three independent experiments (including technical replicates in one). *p* values were calculated using one-way ANOVA followed by Holm-Šidák’s multiple comparisons test relative to the parental cell line. **d** Relative abundance of TUBA1B mRNA in HEK293 parental, TTC5 knockout, and TTC5WT in 10-day-old spheroids, normalized to housekeeping transcripts and parental levels. Data show mean ± SEM from three independent experiments. *p* values reflect one-way ANOVA followed by Holm-Šidák’s multiple comparisons test for each of the indicated cell lines compared to parental spheroids.**p*<0.05, ns - not significant. Exact *p* values can be found in the Source Data file.


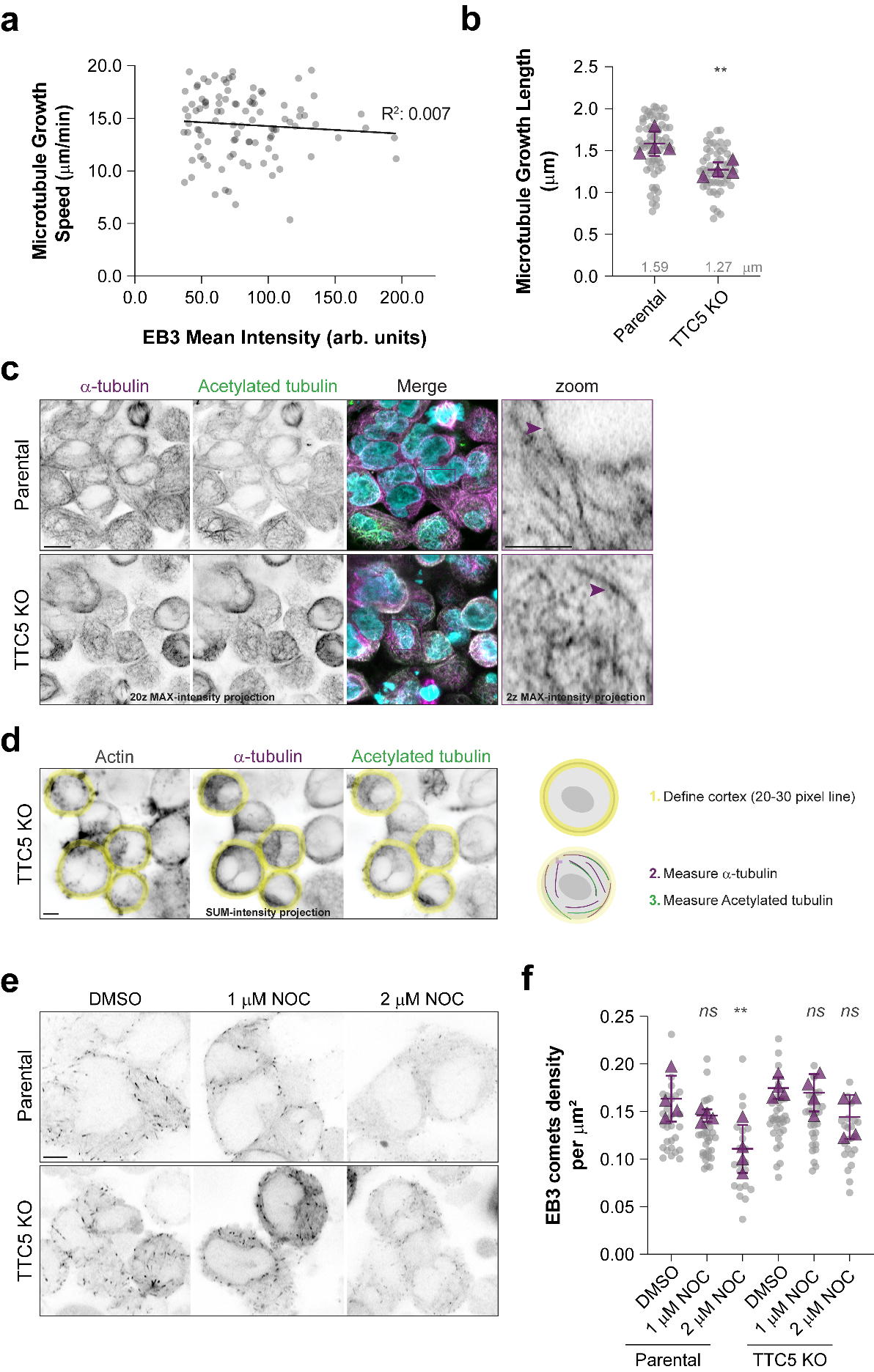
Supplementary Figure 3

**Fig. S3. (related to Fig. 1)** **a** Correlation between microtubule growth speed and cytoplasmic EB3 intensity. Simple linear regression is shown (R2: 0.007), indicating no correlation. **b** Microtubule growth length in the indicated cell lines. Data show mean ± SD from four independent experiments (Parental, *n=*65; TTC5 KO, *n=*54 cells). *p* value indicates an unpaired, two-tailed Student’s *t*-test comparing means between TTC5 KO and the parental cell line. **c** Representative stills of 10-day-old HeLa parental and TTC5 KO spheroids immunostained for total a-tubulin (magenta), acetylated a-tubulin (green), and DNA (DAPI, cyan). Scale bar, 5 mm. Insets show 3x magnification of a maximum-intensity projection of 5 z-planes, highlighting microtubules (magenta arrowhead). **d** Schematic representation of the quantification strategy for α-tubulin and acetylated α-tubulin at the cell cortex. Representative sum-intensity projection images show actin (used to define the cell cortex), α-tubulin, and acetylated α-tubulin channels. **e** HeLa parental and TTC5 KO spheroids stably expressing EB3-EGFP were treated with DMSO or increasing doses of nocodazole (1 and 2 mM) for 1 hour. Scale bar, 5 mm. **f** EB3 comet density per area (mm2) in interphase parental and TTC5 KO cells measured in one frame. Data show mean ± SD from four independent experiments (Parental DMSO, *n=*28, 1 mM NOC, *n=*30, 2 mM NOC, *n=*21; TTC5 KO DMSO, *n=*33 cells, 1 mM NOC, *n=*31, 2 mM NOC, *n=*22). *p* values reflect one-way ANOVA followed by Holm-Šidák’s multiple comparisons test for the indicated treatments compared to their respective DMSO control. ***p<*0.01, ns - not significant. Exact *p* values can be found in the Source Data file.

**Supplementary Figure 4**


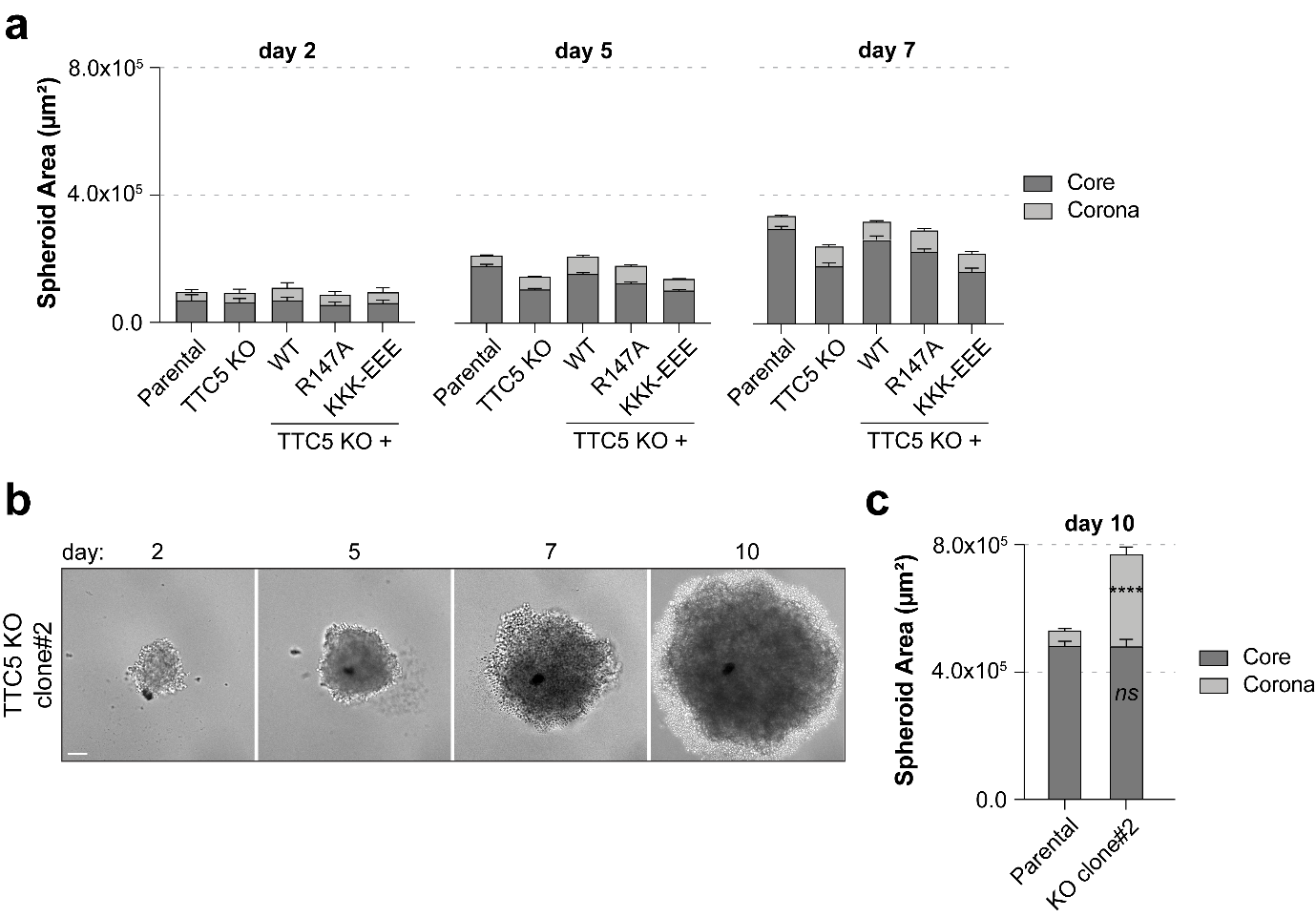


**Fig. S4. (related to Fig. 2) a** Quantification of spheroid core and corona areas from Fig. 2a at day 2, 5, and 7. Data represent mean ± SEM from three independent experiments (Parental, *n=*15/36/25; TTC5 KO, *n=*15/33/18; TTC5*WT*, *n=*15/36/21; TTC5*R147A*, *n=*15/36/23; TTC5*KKK-EEE*, *n=*15/35/17 spheroids) **b** Representative brightfield images of spheroids formed by TTC5 KO (clone#2) cells. Scale bar, 100 mm. **c** Quantification of spheroid core and corona area. Parental spheroids data correspond to Fig. 2b. Data show mean ± SEM from two independent experiments (Parental, *n=*21; TTC5 KO, *n=*23 spheroids). *p* values indicate an unpaired two-tailed Student’s *t*-test comparing TTC5 KO (clone#2) spheroids to parental. *****p<*0.0001, *ns* - not significant. Exact *p* values can be found in the Source Data file.

**
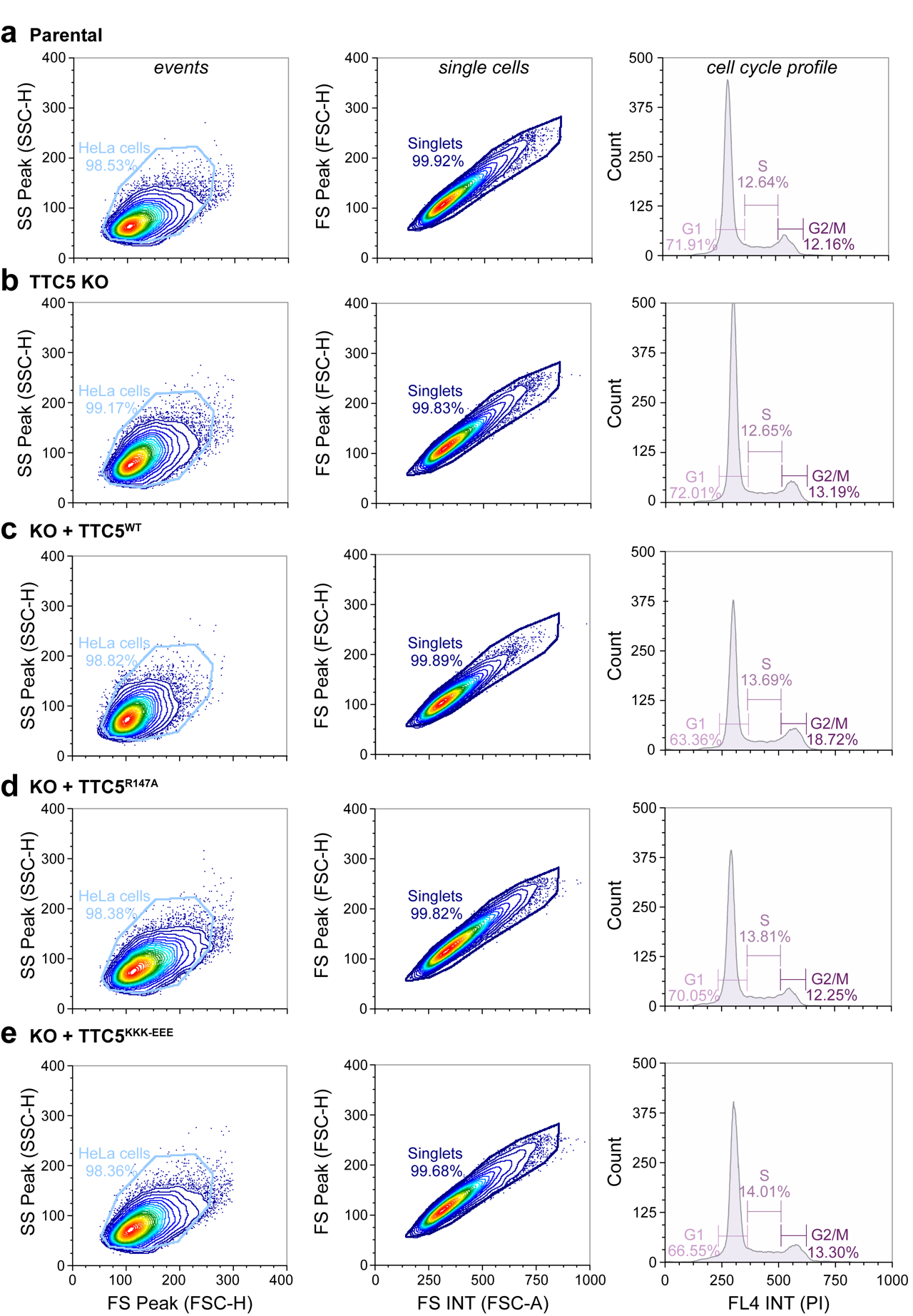
Supplementary Figure 5**

**Fig. S5.** **(related to Fig. 2)** Representative gating strategy used for flow cytometric analysis of single-cell suspensions dissociated from HeLa spheroids of **a** parental, **b** TTC5 KO, **c** TTC5*WT*, **d** TTC5*R147A*, and **e** TTC5*KKK-EEE* cells. The left panel shows side scatter height (SSC-H) versus forward scatter height (FSC-H) used to identify the main cell population. The middle panel shows forward scatter height (FSC-H) versus forward scatter area (FSC-A) used for singlet discrimination. The right panel shows the distribution of propidium iodide fluorescence intensity (PI; FLA4 INT) used for downstream quantification of cell cycle profiles: G1, S and G2/M phases. The full dataset from a minimum of two independent replicates can be found in the Source Data file.


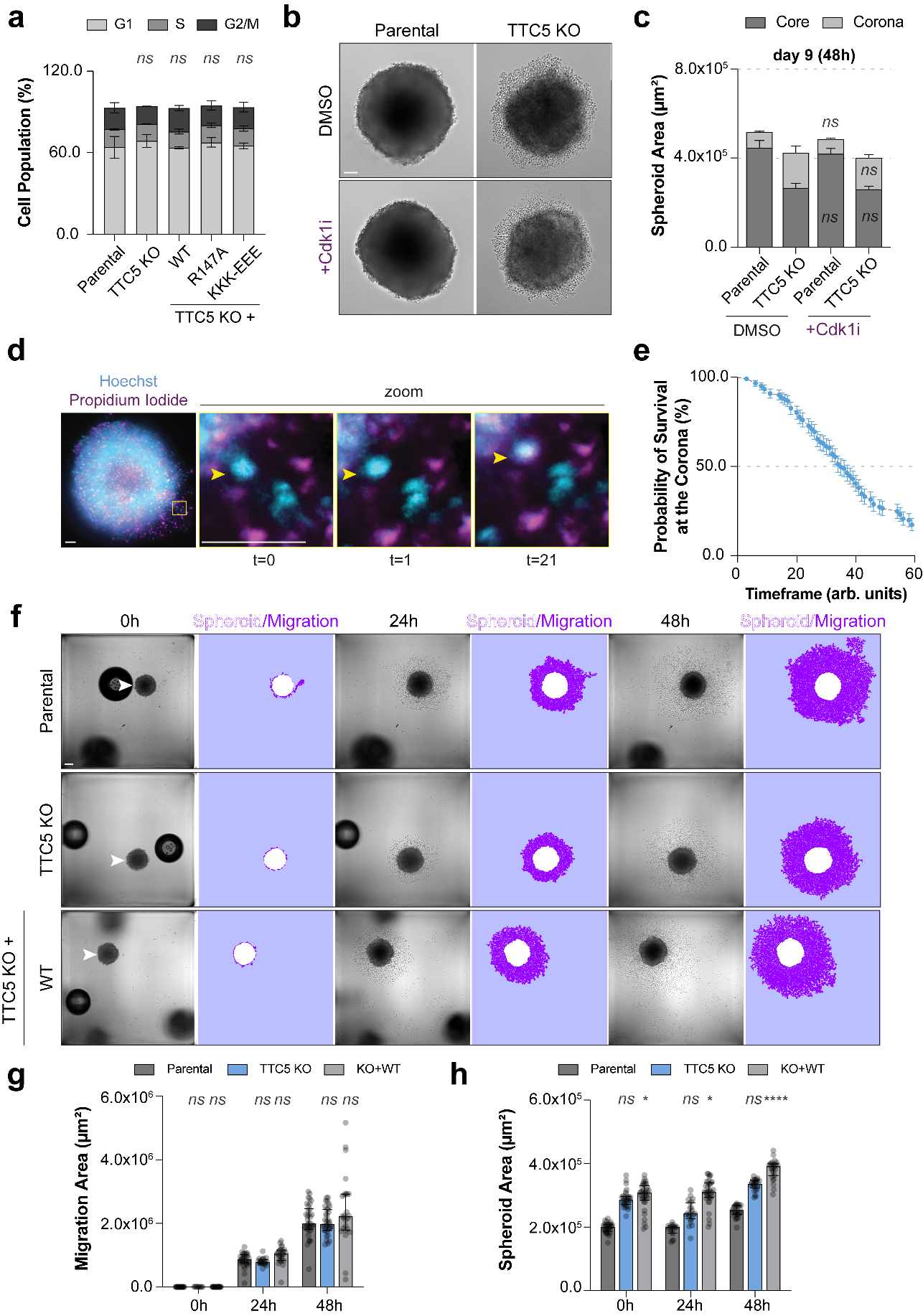
**Supplementary Figure 6**

**Fig. S6.** **(related to Fig. 2)** **a** Flow cytometry analysis of cell cycle distribution using PI to assess DNA content. Data represent mean ± SD from a minimum of two independent experiments. No significant differences were detected by one-way ANOVA. Gating strategy to define cell cycle stages is found in the Source Data file. **b** Representative images of HeLa parental and TTC5 KO spheroids treated with DMSO or 5 mM Ro-3306 (Cdk1 inhibitor) for 48 hours. **c** Quantification of spheroid corona area at day 9. Data show mean ± SEM from two independent experiments (Parental DMSO, *n=*9; TTC5 KO DMSO, *n=*8; Parental Cdk1i, *n=*7; TTC5 KO Cdk1i, *n=*8 spheroids). *p* values reflect one-way ANOVA followed by Holm-Šidák’s multiple comparisons test for Cdk1i-treated spheroids compared to their respective control. **d** Live-cell imaging of TTC5 KO spheroid stained with Hoechst (all nuclei, cyan) and PI (dead nuclei, magenta). Images were acquired every 20 minutes. Yellow arrowhead highlights a cell located at the spheroid corona that dies at timeframe =21 (positive for both Hoechst and PI). Scale bars, 100 mm. **e** Probability of cell survival for cells located at the spheroid corona from two independent experiments. **f** Spheroid migration assay performed for 48 hours using HeLa parental, TTC5 KO, and TTC5WT lines. Segmentation masks show migration area (magenta) and spheroid area (white). White arrowhead highlights the spheroid at timepoint 0 hours. Scale bar, 100 mm. **g** Quantification of migration and **h** spheroid area. Data are shown as median ± IQR from two independent experiments (Migration area: Parental, *n=*30/37/43; TTC5 KO, *n=*16/28/46; TTC5*WT*, *n=*34/36/36 spheroids; Spheroid area: Parental, *n=*36/34/40; TTC5 KO, *n=*30/27/46; TTC5*WT*, *n=*36/35/37 spheroids). *p* values were determined by the Kruskal-Wallis test followed by Dunn’s multiple comparisons test, comparing parental spheroids to the indicated conditions. *****p<*0.0001, **p<*0.05, ns - not significant. Exact *p* values can be found in the Source Data file. **Supplementary Figure 7**

**
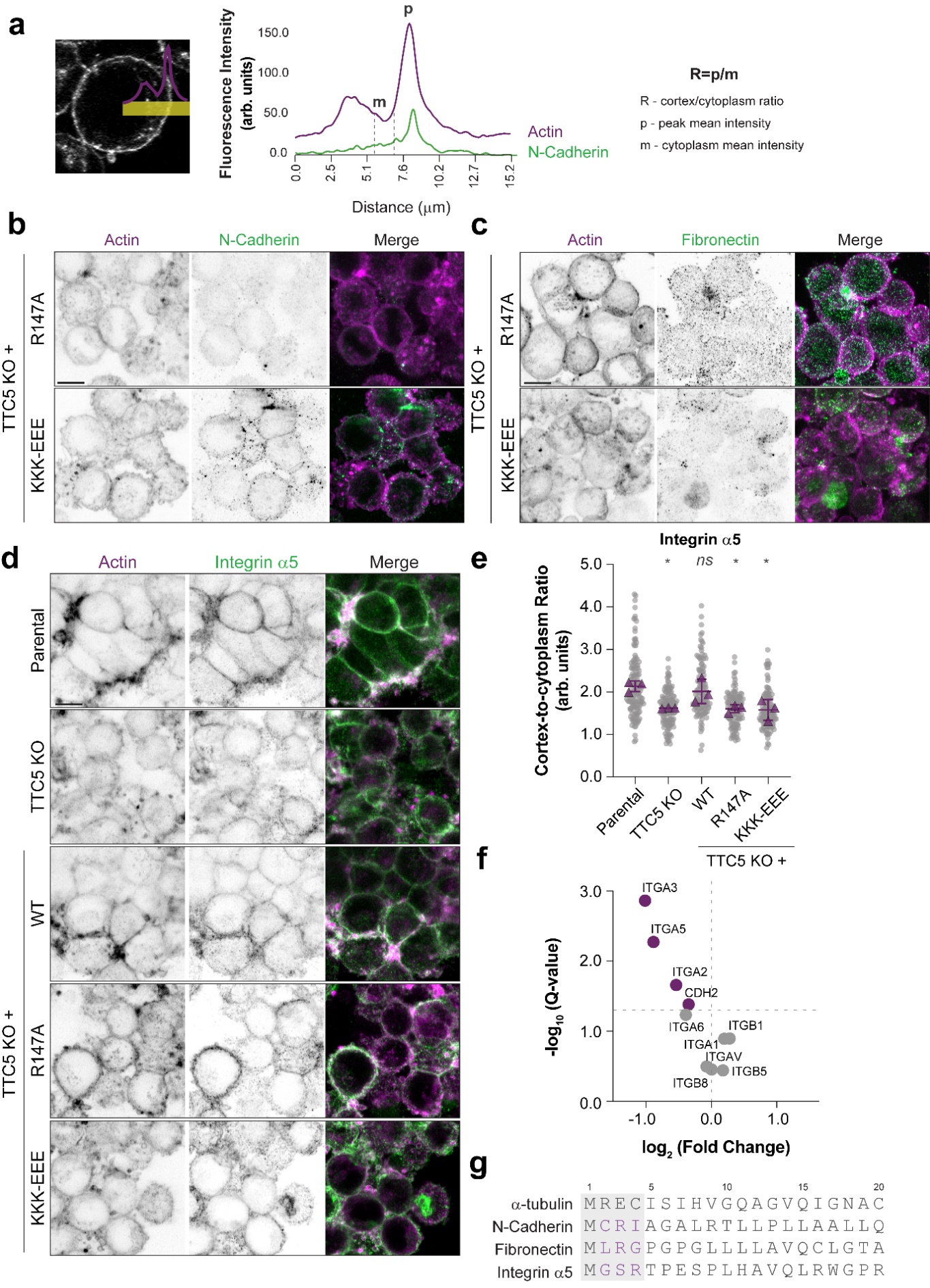
**

**Fig. S7. (related to Fig. 3) a** Schematic for the calculation of the cortex-to-cytoplasmic ratio (R) for actin (magenta) and a protein of interest (e.g., N-Cadherin, green): *p* represents the mean intensity ± 1 mm from cortical peak; *m* represents the mean intensity 1 mm in the cytoplasm, before *p*. Representative confocal images of TTC5*R147A* and TTC5*KKK-EEE* spheroids cultured for 10 days counterstained with phalloidin-647 (magenta, Actin) and immunostained for **b** N-Cadherin (green) or **c** Fibronectin (green). Cortex-to-cytoplasm ratios are shown in Fig. 3b and 3d, respectively. Scale bar, 5 mm. **d** Immunofluorescence of 10-day-old HeLa parental, TTC5 KO, and Flag-TTC5 spheroids stained for Integrin a5 (green) and counterstained with phalloidin-647 (magenta, Actin). Scale bar, 5 mm. **e** Cortex-to-cytoplasm ratio of Integrin a5 fluorescence intensity. Data show mean ± SD from three independent experiments (Parental, *n=*132; TTC5 KO, *n=*121; TTC5*WT*, *n=*113; TTC5*R147A*, *n=*108; TTC5*KKK-EEE*, *n=*110 cells). *p* values were determined by one-way ANOVA followed by Holm-Šidák’s multiple comparisons test, comparing each indicated cell line to the parental line. **f** Volcano plot showing the relative abundance of N-Cadherin (CDH2) and Integrins in TTC5 KO versus parental spheroids, measured by quantitative mass spectrometry. Horizontal dashed line indicates Q-value of 0.05, and vertical dashed lines reflect a fold-change of 1.5. **g** Sequence alignment of the N-terminal of α-tubulin with those of the adhesion proteins analyzed in this study: N-Cadherin, Fibronectin, and Integrin α5 (1-20 amino acids). The TTC5-recognition motif (MREC), present in α-tubulin, is highlighted in the grey rectangle. This motif is absent in the adhesion proteins (magenta). **p<*0.05, ns - not significant. Exact *p* values can be found in the Source Data file.

**
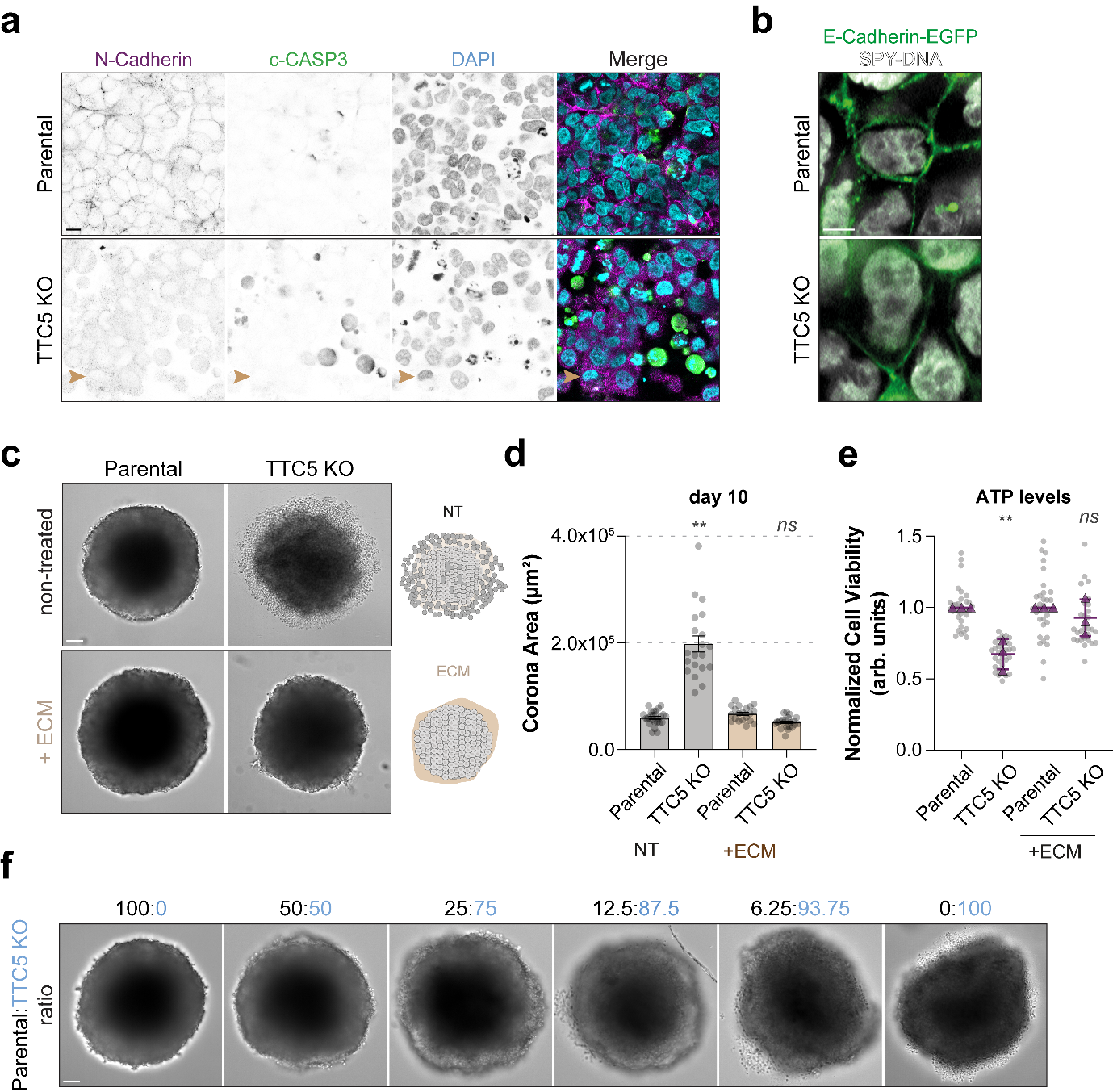
Supplementary Figure 8**

**Fig. S8. (related to Fig. 3)** **a** Immunofluorescence of 10-day-old HeLa parental and TTC5 KO spheroids stained for N-Cadherin (magenta), cleaved-caspase 3 (c-CASP3, green), and DAPI (cyan, DNA). Brown arrowhead highlights a representative TTC5 KO cell, negative for both N-Cadherin and c-CASP3. The staining was reproduced in three independent experiments. Scale bar, 10 mm. **b** Representative confocal images of HeLa parental and TTC5 KO spheroids expressing E-Cadherin-EGFP. E-Cadherin localizes to cell-cell junctions (green) in three biological replicates. SiR-DNA was used to label nuclei (white). **c** Brightfield images of 10-day-old HeLa parental and TTC5 KO spheroids grown in normal medium (non-treated, NT) or supplemented with 1 mg/mL Matrigel (+ECM) between days 5 and 7 days. Scale bar, 100 mm. **d** Spheroid core and corona area quantification at day 10, while showing mean ± SEM from three independent experiments (Parental, *n=*22; TTC5 KO, *n=*16; Parental+ECM, *n=*20; KO+ECM*, n=*22 spheroids). Untreated conditions are also shown in Fig. 3g. *p* values indicate an unpaired two-tailed Student’s *t-*test comparing TTC5 KO spheroids to parental per condition. **e** Cell viability determined by ATP content across the indicated cell lines, normalized to parental spheroids per condition. Data show mean ± SD from three independent experiments (Parental, *n=*33; TTC5 KO, *n=*31; Parental+ECM, *n=*34; KO+ECM, *n=*28 spheroids). *p* values were determined by one-way ANOVA followed by Holm-Šidák’s multiple comparisons test comparing TTC5 KO spheroids to parental. **f** Representative images of spheroids formed by parental and TTC5 KO cells at the indicated ratios. Spheroid morphology was rescued with as few as 25% of parental cells in three independent experiments. ***p<*0.01, ns - not significant. Exact *p* values can be found in the Source Data file.

**
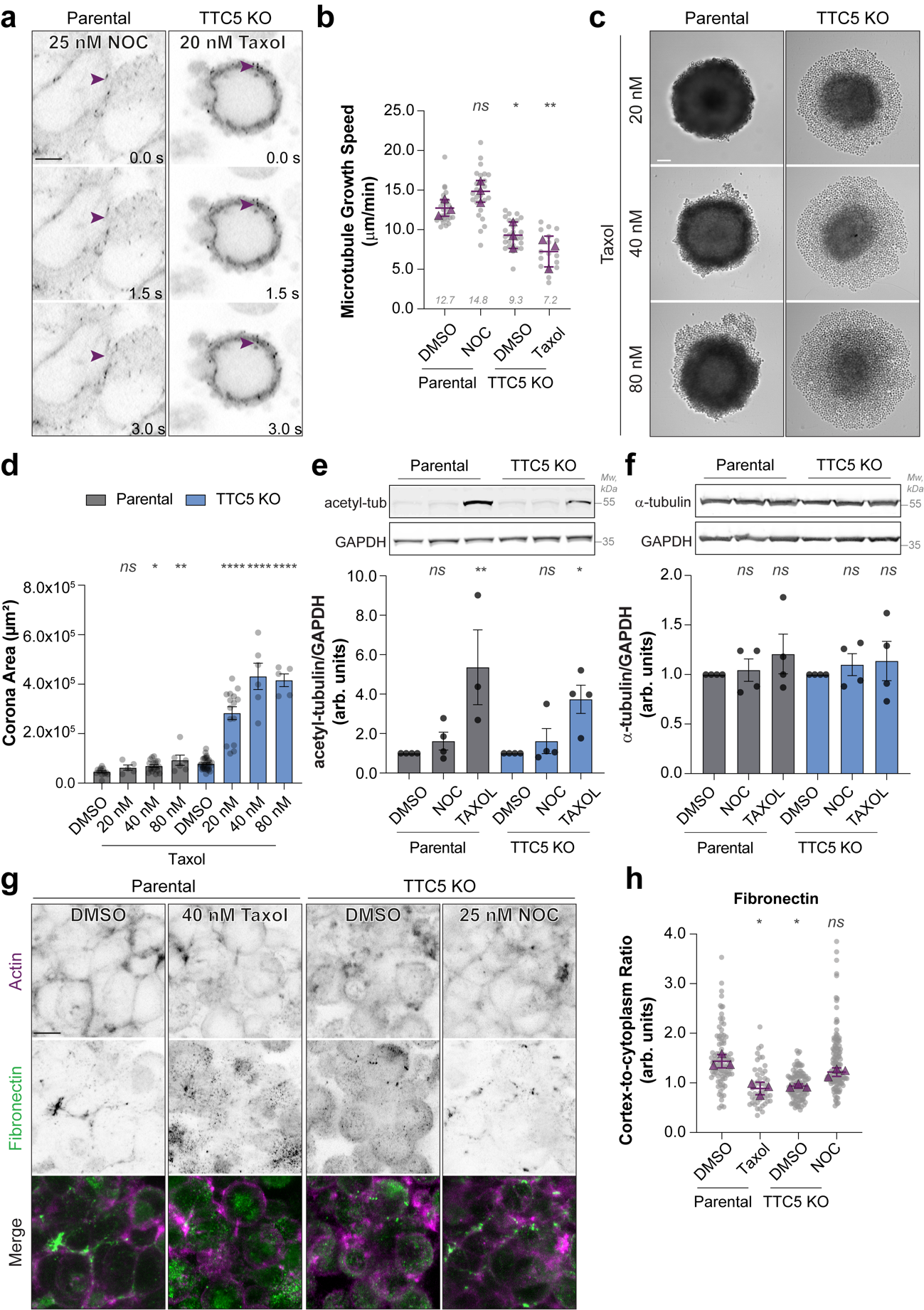
Supplementary Figure 9**

**Fig. S9.** **(related to Fig. 4) a** Time-lapse imaging of HeLa parental and TTC5 KO spheroids stably expressing EB3-EGFP, showing microtubule plus-end tracking (1.5-second interval) after treatment with DMSO, nocodazole, or taxol for 48 hours. The remaining conditions can be found in Fig. 4c-d. The magenta arrowhead follows a representative microtubule growth event over time. Scale bar, 5 mm. **b** Microtubule growth speed measured over four to six consecutive frames. Data show mean ± SD from three independent experiments (Parental DMSO, *n=*28, NOC, *n=*26; TTC5 KO DMSO, *n=* 27, taxol, *n=*17 cells). p values were determined by one-way ANOVA followed by Holm-Šidák’s multiple comparisons test, comparing each condition with its respective control. (c) Brightfield images of 7-day spheroids from HeLa parental and TTC5 KO treated with increasing concentrations of taxol (20, 40, and 80 nM) for 48 hours. Scale bar, 100 mm. (d) Quantification of spheroid areas across the different concentrations at day 7 (48 hours). DMSO and taxol (40/20 nM, for parental and TTC5 KO, respectively) data are shown in Fig. 4b. Data shown mean ± SEM (Parental DMSO, *n=*13; 20nM, *n=*5; 40 nM, *n=*18; 80nM, *n=*6; TTC5 KO DMSO, *n=*31; 20nM, *n=*15; 40nM, *n=*6; 80nM, *n=*5 spheroids). *p* values were determined by one-way ANOVA followed by Holm-Šidák’s multiple comparisons method, comparing each condition with parental DMSO-treated spheroids. Western blot analysis of **e** acetylated tubulin, and **f** a-tubulin protein levels normalized to GAPDH (loading control) in the indicated HeLa spheroids after treatment. Data show mean ± SEM from a minimum of three independent experiments normalized to parental or TTC5 KO cell line. *p* values were determined by one-way ANOVA. **g** Confocal images of 7-day-old HeLa parental and TTC5 KO spheroids post-treatment, stained for Fibronectin (green), phalloidin-647 (magenta, Actin), and DAPI (cyan, DNA). Scale bar, 5 mm. **h** Cortex-to-cytoplasm fibronectin fluorescence intensity. Data show median ± IQR from three independent experiments (Parental DMSO, *n=*142; taxol*, n=*94; TTC5 KO DMSO, *n=*219; NOC, *n=*238 cells)*. p* values were calculated using one-way ANOVA followed by Holm-Šidák’s multiple comparisons test, comparing each condition to parental DMSO-treated spheroids. *****p<*0.0001, ***p<*0.01, **p<*0.05, ns - not significant. Exact *p* values can be found in the Source Data file.
