# Supplementary material for: Tubulin autoregulation tunes microtubule dynamics to support multicellular architecture and viability
Source: Nat Commun. 2026 Jul 22;17:6813. doi: 10.1038/s41467-026-75341-w (PMC13392243; doi:10.1038/s41467-026-75341-w)
Supplement: Supplementary file 2 — Description of Additional Supplementary Files [file 41467_2026_75341_MOESM2_ESM.docx]

Description of Additional Supplementary Files

Title: Supplementary Data 1

Description: Extended mass spectrometry dataset relative to Figure 1E and Figure S7F.

Title: Supplementary Data 2

Description: Protein intensity values for each protein across all samples analyzed by mass spectrometry
