# Supplementary material for: Tubulin autoregulation tunes microtubule dynamics to support multicellular architecture and viability
Source: Nat Commun. 2026 Jul 22;17:6813. doi: 10.1038/s41467-026-75341-w (PMC13392243; doi:10.1038/s41467-026-75341-w)
Supplement: Supplementary file 5 — Reporting Summary [file 41467_2026_75341_MOESM5_ESM.pdf]

nature portfolio

Corresponding author(s): Ivana Gasic and Ana Coelho Almeida

Last updated by author(s): Jun 15, 2026



# Reporting Summary

Nature Portfolio wishes to improve the reproducibility of the work that we publish. This form provides structure for consistency and transparency in reporting. For further information on Nature Portfolio policies, see our Editorial Policies and the Editorial Policy Checklist.

## Statistics

For all statistical analyses, confirm that the following items are present in the figure legend, table legend, main text, or Methods section.

| n/a | Confirmed | |
|---|---|---|
| ☐ | ☒ | The exact sample size (*n*) for each experimental group/condition, given as a discrete number and unit of measurement |
| ☐ | ☒ | A statement on whether measurements were taken from distinct samples or whether the same sample was measured repeatedly |
| ☐ | ☒ | The statistical test(s) used AND whether they are one- or two-sided<br>*Only common tests should be described solely by name; describe more complex techniques in the Methods section.* |
| ☒ | ☐ | A description of all covariates tested |
| ☐ | ☒ | A description of any assumptions or corrections, such as tests of normality and adjustment for multiple comparisons |
| ☐ | ☒ | A full description of the statistical parameters including central tendency (e.g. means) or other basic estimates (e.g. regression coefficient) AND variation (e.g. standard deviation) or associated estimates of uncertainty (e.g. confidence intervals) |
| ☐ | ☒ | For null hypothesis testing, the test statistic (e.g. *F*, *t*, *r*) with confidence intervals, effect sizes, degrees of freedom and *P* value noted<br>*Give P values as exact values whenever suitable.* |
| ☒ | ☐ | For Bayesian analysis, information on the choice of priors and Markov chain Monte Carlo settings |
| ☒ | ☐ | For hierarchical and complex designs, identification of the appropriate level for tests and full reporting of outcomes |
| ☐ | ☒ | Estimates of effect sizes (e.g. Cohen's *d*, Pearson's *r*), indicating how they were calculated |

*Our web collection on statistics for biologists contains articles on many of the points above.*

## Software and code

Policy information about availability of computer code

| | |
|---|---|
| Data collection | Software built in the microscope for image acquisition: Zeiss ZEN (blue edition, 3.3.89.0008), NIS Elements (Nikon), and MetaXpress (Molecular Devices). Sapphire system (Azure Biosystems) was used for WB acquisition. |
| Data analysis | Fiji (ImageJ, 1.54f), R/ R Studio (v2022.12.0+353), GraphPad Prism 8 and Microsoft Excel were used for data analysis.<br>u-track, Matlab package, was used for EB3 comet tracking (https://github.com/DanuserLab/u-track) and Spectronaut (Biognosys) for MS protein identification and quantification.<br>Figures were generated in Adobe Illustrator 2023 and 2025. |

For manuscripts utilizing custom algorithms or software that are central to the research but not yet described in published literature, software must be made available to editors and reviewers. We strongly encourage code deposition in a community repository (e.g. GitHub). See the Nature Portfolio guidelines for submitting code & software for further information.

## Data

Policy information about availability of data

All manuscripts must include a data availability statement. This statement should provide the following information, where applicable:

- Accession codes, unique identifiers, or web links for publicly available datasets
- A description of any restrictions on data availability
- For clinical datasets or third party data, please ensure that the statement adheres to our policy

The proteomics data generated in this study have been deposited in the available in the PRIDE repository under accession code PXD076805.

## Research involving human participants, their data, or biological material

Policy information about studies with human participants or human data. See also policy information about sex, gender (identity/presentation), and sexual orientation and race, ethnicity and racism.

| | |
|---|---|
| Reporting on sex and gender | *Use the terms sex (biological attribute) and gender (shaped by social and cultural circumstances) carefully in order to avoid confusing both terms. Indicate if findings apply to only one sex or gender; describe whether sex and gender were considered in study design; whether sex and/or gender was determined based on self-reporting or assigned and methods used.*<br>*Provide in the source data disaggregated sex and gender data, where this information has been collected, and if consent has been obtained for sharing of individual-level data; provide overall numbers in this Reporting Summary. Please state if this information has not been collected.*<br>*Report sex- and gender-based analyses where performed, justify reasons for lack of sex- and gender-based analysis.* |
| Reporting on race, ethnicity, or other socially relevant groupings | *Please specify the socially constructed or socially relevant categorization variable(s) used in your manuscript and explain why they were used. Please note that such variables should not be used as proxies for other socially constructed/relevant variables (for example, race or ethnicity should not be used as a proxy for socioeconomic status).*<br>*Provide clear definitions of the relevant terms used, how they were provided (by the participants/respondents, the researchers, or third parties), and the method(s) used to classify people into the different categories (e.g. self-report, census or administrative data, social media data, etc.)*<br>*Please provide details about how you controlled for confounding variables in your analyses.* |
| Population characteristics | *Describe the covariate-relevant population characteristics of the human research participants (e.g. age, genotypic information, past and current diagnosis and treatment categories). If you filled out the behavioural & social sciences study design questions and have nothing to add here, write "See above."* |
| Recruitment | *Describe how participants were recruited. Outline any potential self-selection bias or other biases that may be present and how these are likely to impact results.* |
| Ethics oversight | *Identify the organization(s) that approved the study protocol.* |

Note that full information on the approval of the study protocol must also be provided in the manuscript.

# Field-specific reporting

Please select the one below that is the best fit for your research. If you are not sure, read the appropriate sections before making your selection.

☒ Life sciences ☐ Behavioural & social sciences ☐ Ecological, evolutionary & environmental sciences

For a reference copy of the document with all sections, see nature.com/documents/nr-reporting-summary-flat.pdf

# Life sciences study design

All studies must disclose on these points even when the disclosure is negative.

| | |
|---|---|
| Sample size | For single cell measurements, between 45-240 cells were analyzed per condition. For spheroid analysis, a minimum of 5 spheroids were quantified per condition. Exact number of cells/spheroids is indicated in the figure legends or methods section. |
| Data exclusions | ROUT method (Q=1%) was used to remove outliers, as indicated in the Methods and Source Data file. |
| Replication | For each experiment, the number of biological replicates is indicated in the figure legends. |
| Randomization | Randomization was not suitable with this study as the indicated biological samples all have distinct genotypes or drug treatments, and there were no inherent biases in any of the applied assays. |
| Blinding | Blinding was not relevant to this study because the experimentalist had to prepare the samples and treatments. However, all data analysis was streamlined and automated whenever possible such that no bias is invoked. |

# Reporting for specific materials, systems and methods

We require information from authors about some types of materials, experimental systems and methods used in many studies. Here, indicate whether each material, system or method listed is relevant to your study. If you are not sure if a list item applies to your research, read the appropriate section before selecting a response.

## Materials & experimental systems

| n/a | Involved in the study | |
|---|---|---|
| ☐ | ☒ | Antibodies |
| ☐ | ☒ | Eukaryotic cell lines |
| ☒ | ☐ | Palaeontology and archaeology |
| ☒ | ☐ | Animals and other organisms |
| ☒ | ☐ | Clinical data |
| ☒ | ☐ | Dual use research of concern |
| ☒ | ☐ | Plants |

## Methods

| n/a | Involved in the study | |
|---|---|---|
| ☒ | ☐ | ChIP-seq |
| ☐ | ☒ | Flow cytometry |
| ☒ | ☐ | MRI-based neuroimaging |

## Antibodies

Antibodies used

anti-alpha-tubulin (WB - 1:5000; IF - 1:200, Proteintech, #11224-1-AP, Lot nº 0013194)
anti-beta-tubulin (WB - 1:1500, ABCD Antibodies, #AA344)
anti-acetyl-tubulin (Lys40) (WB - 1:1500, Proteintech, #66200-1-Ig, Clone No.7E5H8)
anti-GAPDH (WB - 1:10000, Cell Signaling Technology, #2118S, Lot nº 10)
LI-COR 550 (1:10000, Azure Biosystems, #AC2159, Lot nº 240322-56)
LI-COR 800 (1:5000, Thermo Fisher Scientific, #A32735, Lot nº WA318266)
LI-COR 680 (1:5000, Thermo Fisher Scientific, #A32729)
anti-acetylated tubulin (1:200, Sigma-Aldrich, #T7451, Lot nº 0000312701)
anti-N-Cadherin (1:200, Proteintech, #22018-1-AP, Lot nº 00111704)
anti-Fibronectin (1:1000, clone 1801, kind gift from Prof. Bernhard Wehrle-Haller, University of Geneva)
anti-Integrin α5 (1:10, AA430-M2a, kind gift from Prof. Bernhard Wehrle-Haller, University of Geneva)
anti-cleaved Caspase3 (1:200, Proteintech, #68773-1, Lot nº 10041389)
Alexa 488 (1:500, Thermo Fisher Scientific, #A32731, Lot nº WC318798)
Alexa 555 (1:500, Thermo Fisher Scientific, #A32727, Lot nº WA316324)

Validation

Antibody specificity was based on validation data provided by the manufacturers. No newly developed antibodies were used in this study.

## Eukaryotic cell lines

Policy information about cell lines and Sex and Gender in Research

Cell line source(s)

Flp-In T-REx HeLa and HEK cells - Thermo Fisher Scientific.

Authentication

None of the cell lines used were authenticated.

Mycoplasma contamination

Cell lines used in this study were regularly tested for mycoplasma contaminations.

Commonly misidentified lines
(See ICLAC register)

*Name any commonly misidentified cell lines used in the study and provide a rationale for their use.*

## Plants

Seed stocks

*Report on the source of all seed stocks or other plant material used. If applicable, state the seed stock centre and catalogue number. If plant specimens were collected from the field, describe the collection location, date and sampling procedures.*

Novel plant genotypes

*Describe the methods by which all novel plant genotypes were produced. This includes those generated by transgenic approaches, gene editing, chemical/radiation-based mutagenesis and hybridization. For transgenic lines, describe the transformation method, the number of independent lines analyzed and the generation upon which experiments were performed. For gene-edited lines, describe the editor used, the endogenous sequence targeted for editing, the targeting guide RNA sequence (if applicable) and how the editor was applied.*

Authentication

*Describe any authentication procedures for each seed stock used or novel genotype generated. Describe any experiments used to assess the effect of a mutation and, where applicable, how potential secondary effects (e.g. second site T-DNA insertions, mosiacism, off-target gene editing) were examined.*

# Flow Cytometry

## Plots

Confirm that:

- [x] The axis labels state the marker and fluorochrome used (e.g. CD4-FITC).
- [x] The axis scales are clearly visible. Include numbers along axes only for bottom left plot of group (a 'group' is an analysis of identical markers).
- [x] All plots are contour plots with outliers or pseudocolor plots.
- [x] A numerical value for number of cells or percentage (with statistics) is provided.

## Methodology

| | |
|---|---|
| Sample preparation | Sample preparation details are available at the Material and Methods section "Cell cycle profiles". |
| Instrument | Gallios, Model 2L/8C (Beckman Coulter). |
| Software | Data analysis was performed using Kaluza, version 2.1 (Beckman Coulter) and FCS Express 7. |
| Cell population abundance | Between 5,000 and 30,000 cells were evaluated for each sample. The full dataset and corresponding cell-cycle profiles are available in the Source Data file. |
| Gating strategy | Cells were gated based on FSC/SSC and FS Peak/FS INT parameters to eliminate cell debris and doublets, respectively. The complete gating strategy is provided in Fig. S5a–e. |

- [x] Tick this box to confirm that a figure exemplifying the gating strategy is provided in the Supplementary Information.
