# Supplementary material for: Tubulin autoregulation tunes microtubule dynamics to support multicellular architecture and viability
Source: Nat Commun. 2026 Jul 22;17:6813. doi: 10.1038/s41467-026-75341-w (PMC13392243; doi:10.1038/s41467-026-75341-w)
Supplement: Supplementary file 6 — Transparent Peer Review file [file 41467_2026_75341_MOESM6_ESM.pdf]

nature portfolio

Peer Review File

Tubulin autoregulation tunes microtubule dynamics to support multicellular architecture and viability

Corresponding Author: Dr Ivana Gasic

This file contains all reviewer reports in order by version, followed by all author rebuttals in order by version.

Version 0:

Reviewer comments:

Reviewer #1

(Remarks to the Author)
The manuscript by Almeida et al. investigates tissue-level consequences of tubulin autoregulation using HeLa cell spheroids as a 3D multicellular model. The authors report that disrupting tubulin autoregulation by knocking out TTC5, a protein that recognizes tubulin mRNAs and recruits other proteins leading to mRNA degradation, causes elevated cytosolic tubulin levels, microtubule network hyperstabilization and altered tissue architecture due to altered cell adhesions and cell death in the spheroids. The study aims to shed light on the biological functions of tubulin autoregulation in 3D tissues, which have mostly been studied thus far in 2D cultures and under conditions of microtubule drug treatments to artificially induce tubulin autoregulator pathways. Overall, this work contributes to our understanding of how global tubulin levels are tuned to support tissue dynamics and architecture.

This is an interesting study addressing the biological significance of tubulin autoregulation, a process that remains largely unexplored. A key strength is the use of a tractable HeLa cell spheroid system to test tubulin autoregulatory mechanisms in a 3D context, which has not been previously examined. Notably, disrupting autoregulation leads to a modest increase in total tubulin and selective enrichment of certain isoforms, but not others. The resulting decrease in cell viability upon disruption of autoregulation highlights the importance of appropriately sensing and regulating soluble tubulin levels in tissue-like environments.
Several issues limit the impact of the work in its current form, particularly concerning the link between disrupted autoregulation, altered tubulin levels, and microtubule stability. Despite elevated acetylation (a marker of microtubule stability) microtubule growth lifetimes remain unchanged, and, counter intuitively, growth rates are slower despite higher total tubulin levels. Thus, the connection between altered tubulin regulation and microtubule stability or dynamics is not clearly established, and it remains unclear whether the reduced viability arises directly from altered microtubule stability or through indirect effects. With additional quantification and a clearer interpretation of the tubulin and stability data, however, the study could make a valuable contribution to understanding how autoregulation influences microtubule organization and function in 3D contexts.

Major comments

Figure 1.

The authors report that TTC5 KO increases tubulin mRNA and soluble tubulin in day 10 spheroids. However, the statement “Mass Spectrometry corroborated the enrichment of most a- and b-tubulin isotypes in TTC5 KO spheroids” overstates the result as only a subset of isotypes (4 of 12) are significantly enriched in Fig. 1E. Furthermore, quantification of total tubulin protein levels suggests that they are only mildly increased (P=0.05, which is just approaching statistical significance) in TTC5 KO cells (extended fig 1D). Please change the wording to reflect the modest effect on total tubulin levels and that this is restricted to certain enriched isotypes – perhaps it is the relative tubulin isotype composition that is important, rather than the global levels per se? In Fig. 1D/E, TUBA4A protein is increased in TTC5 KO spheroids while its mRNA is not statistically elevated. Please discuss possible reasons (e.g., post-transcriptional regulation, increased protein stability / changes in protein degradation pathways). Extended Fig. B and C lack statistical significance reporting, so while we can see from 1E that TUBB and TUBAIB protein levels are not elevated in TTC5 KO, it’s not clear how this relates to mRNA levels. Why are some isotypes affected more than others? Overall, the findings could be made clearer by explicitly stating which tubulin isoform mRNAs are significantly changed and which are not, and then mapping which mRNA changes

correspond to which tubulin protein changes (and which do not).

The authors interpret increased acetylated-tubulin staining as “hyper-stabilized” microtubules. However, their live-imaging quantification at day 5 shows unchanged growth lifetime (i.e. time spent in the growth phase equating to stability) while reporting slower growth rates. Acetylation is commonly taken as a marker enriched on longer-lived, low-turnover microtubules, but unchanged growth lifetime argues against an overall increase in stability. A simple prediction based on in vitro data would be that elevated tubulin concentration alone accelerates microtubule growth. Therefore, the manuscript should discuss this apparent contradiction and offer plausible mechanisms. Two technical limitations render the current interpretation uncertain (i) acetylation was quantified only at the cortex, whereas the dynamics measurements (e.g., Fig. 1F) appear to have been taken across the cytoplasm, meaning distinct microtubule populations are being compared, and (ii) microtubule dynamics were measured in day 5 spheroids, while protein and mRNA levels were measured at day 10. I recommend the authors provide whole-cell quantification of acetylated tubulin normalized to total tubulin to determine whether the increase is global or cortex-restricted, and the authors should measure microtubule dynamics at day 10 (when tubulin mRNA and protein were quantified) otherwise the link between tubulin protein levels and microtubule dynamics remains speculative. Including orthogonal stability assays (e.g., nocodazole or cold treatment) would also help to substantiate the findings; or at minimum, acknowledge that increased acetylation may reflect a redistribution of stable microtubule subpopulations rather than a cell-wide increase in stability.

Figure 2.

By day 10, the authors state that the corona area in TTC5KO spheroids “equaled or exceeded” the size of the core. However, quantification in Fig. 2B and Extended Fig. 2C suggests that while the corona is larger in TTC5KO than in parental spheroids, it does not exceed the size of the core. A clearer way to represent this would be to plot the core-to-corona area ratio for each spheroid, which would allow direct visualization of relative expansion and clarify whether the corona truly surpasses the core in size, if that is relevant.

For the viability data (Fig. 2C), please clarify whether the luminescence (ATP) readouts were normalized to total cell number per spheroid, as differences in ATP content could otherwise reflect variation in cell number rather than true differences in viability. Regarding the statement that “cell detachment and accumulation at the spheroid corona preceded cell death (Extended Data Fig. 3D), implicating defective adhesion rather than necrotic core dispersion,” only a single example is shown. This claim should be quantified, as currently only a single example is shown.
Finally, it is important to clarify whether dying or apoptotic cells were excluded from the analyses of microtubule acetylation, as cell death could independently alter microtubule organization. This distinction is crucial to determine whether the observed microtubule phenotype represents a cause or consequence of reduced viability.

Figure 3.

The quantification of data in 3A-B (and corresponding extended data) appears to rely on a line scan from cell center to periphery, which could be problematic when the cortical signal is non-homogeneous; perhaps a more representative metric would be to measure fluorescence intensity along the entire cell perimeter (as done for acetylated-tubulin in Fig. 1).
Figure 3E – Could the observed cell rounding and loss of cell–cell junctions also reflect activation of an early cell death program (e.g. apoptosis induced by tubulin imbalance, leading to secondary junction loss) rather than a direct effect of altered tubulin levels on adhesion complexes? Can the authors distinguish between these possibilities, for example, by assessing earlier apoptosis markers (e.g., caspases, Annexin V, or nuclear morphology) in these conditions? Why is the aspect ratio phenotype not rescued with WT TTC5 in the graph in 3E?

It would be helpful to also show the state of the microtubule network in the same cells displayed in Figs. 3A and C. This would strengthen the connection between tubulin levels, microtubule organization, and adhesion phenotypes.

Figure 4.

Figure 4 requires clarification before the claims about a restored cortical corona and N-cadherin localization are convincing. I cannot see a defined “corona” in the images shown in Fig. 4A; it is not clear which feature the authors are labeling as the corona nor why day 7 spheroids were used here when the manuscript otherwise emphasizes day 10 as the point where phenotypes are most robust (Fig. 2). Please repeat the analysis at day 10 (or justify the choice of day 7) and include clearer annotations that define the corona and its boundaries as well as quantification of both core and corona area.

For Fig. 4D, the statement that “low-dose nocodazole reinstated cortical N-cadherin localization” requires statistical support: is the cortical N-cadherin signal in parental + DMSO statistically non significantly different from TTC5KO + low-dose nocodazole? Are integrin and fibronectin localization, and cell viability are also restored by nocodazole treatment? Have the authors measured the additional effects of nocodazole and taxol on cell migration and division in these experiments, which also contribute to tissue architecture and integrity.

Finally, penetration of microtubule-targeting agents in 3D spheroids is a concern. Have the authors verified that the drug treatments alter microtubule dynamics throughout the spheroid (edge versus intermediate versus core) rather than only at the periphery. Without this, it is difficult to determine whether rescue of adhesion/corona phenotypes is due to a uniform effect on microtubules throughout the spheroid tissue.

Minor comments

1. Y-axis on graphs in 3B/D should say cortex-to-cytoplasm F.I ratio.
2. Y-axis in Figure 2C should state what is actually being measured (ATP luminescence?)
3. Missing citation for Walker et al., J Cell Biol 1988, in introduction, as demonstration of microtubule growth rate scaling with tubulin concentration in vitro.
4. The spheroid migration assay requires a more detailed methodological description and definition of the "migration area" for those not familiar with the assay. Do individual cells migrate within the spheroids, and could this be measured as well?
5. Microtubule growth length is presumably calculated over a set period of time but this is not noted, and should be clarified. Given that they report no effect of TTC5 KO on microtubule lifetimes, this is essentially another way of reporting the growth rate.

Reviewer #2

(Remarks to the Author)
In this study, Almeida et al. explore the biological significance of tubulin autoregulation, a feedback mechanism that maintains proper levels of α- and β-tubulin proteins. Using human 3D (HeLa) cell spheroids, the authors show that loss of TTC5 (by knockout (KO)) or TTC5 mutants that disable tubulin autoregulation cause accumulation of soluble tubulin (mRNA and protein). Accumulation of α- and β-tubulin, in turn, leads to decreased microtubule growth rates (assessed by EB3 tracking), increased acetylation of α-tubulin K40, and a redistribution of microtubules towards the cortex. The authors then show that tubulin autoregulation defective spheroids exhibit an abnormal architecture characterized by a dispersive "corona" of detached cells; detached cells have an increased propensity to die. Detachment appears to be caused by impaired cell-cell and cell-matrix adhesion; TTC5 KO and TTC5 mutant spheroids showed reduced cortical localization of N-cadherin, integrin a5, and fibronectin relative to wild type spheroids. The effects of deleting TTC5 or tubulin autoregulation defective TTC5 mutants could be rescued by reintroducing functional TTC5, E-cadherin overexpression, or modulating microtubule dynamics with mild destabilization (nocodazole). In contrast, taxol, which stabilizes microtubules, worsened phenotypes caused by defects in tubulin autoregulation. In summary, the authors demonstrate that tubulin autoregulation is important for fine-tuning microtubule dynamics, and that this in turn maintains cell-cell and cell-matrix adhesion, specifically in spheroids. This work suggests that tubulin autoregulation safeguards spheroid organization, connecting molecular control of tubulin synthesis to higher-order structural integrity.

Concerns
1. Previous work by Lin et al. (2020) (Gasic was one of the investigators on this manuscript) showed that defects in tubulin autoregulation did not change tubulin protein levels in monolayer cultures (tubulin mRNA levels did increase), which is at odds with findings in this work. The authors speculate that cell context is important, that is, cells organized into a 3D structure respond differently than cells in 2D. This explanation is not terribly satisfying, but I understand that it may be beyond the scope of this paper to determine the mechanism(s) that underlies this difference. It would be worthwhile, however, to show that a cell line other than HeLa responds in a similar manner.

2. It is puzzling that microtubule growth rates are slower in tubulin autoregulation defective cells. In many (most) contexts, microtubule growth rates mirror soluble tubulin concentration: microtubules grow faster as tubulin levels increase. The authors offer no explanation for this paradox. Please address.

3. The authors show western blot data for alpha-tubulin (Extended Data Figure 1D) to argue that tubulin levels are elevated. Please also show western blot data for beta-tubulin.

4. In Figure 4, the authors show that nocodazole rescues the dispersed phenotype of tubulin autoregulation spheroids, whereas taxol worsens the phenotype. The authors speculate that low dose nocodazole restores microtubule dynamics, which I take to mean that microtubule growth rates should increase in the presence of low dose nocodazole. This is counterintuitive (see Point 2). It would be useful to measure microtubule growth rates (by tracking of EB3-GFP comets as performed in Figure 1) in nocodazole- and taxol-treated spheroids.

5. It is not clear if the authors omitted mitotic cells when quantifying the levels of K40 acetylated alpha-tubulin. Mitotic spindle microtubules are highly acetylated (Figure 1H), so the authors should comment on how they dealt with dividing cells (~5-10% for HeLa cells in monolayer).

6. It does not appear to me that reintroduction of TTC5 into TTC5 KO cells rescues the levels of fibronectin in spheroid cultures (Figure 3C).

7. There are no data (western blotting, or cell imaging of fluorescent E-cadherin) to show that E-Cadherin is actually overexpressed in HeLa cells (Figures 3F-H).

Reviewer #3

(Remarks to the Author)
In this manuscript, Ana Almeida and colleagues study the role of tubulin autoregulation, a feedback mechanism controlling tubulin protein synthesis, in maintaining microtubule dynamics, cell adhesion, and tissue integrity. By using 3D human cell spheroids, they show that loss of the key regulator TTC5 disrupts this pathway, leading to excess tubulin, hyperstable

microtubules, and compromised cell–cell and cell–matrix adhesion, disorganized multicellular architecture and reduced cell viability. This work may contribute to a better understanding of diseases involving TTC5 or SCAPER and may open new avenues for targeted therapies that modulate microtubule stability or tubulin levels. While the topic is potentially interesting, the authors fail to clearly demonstrate their claim that TTC5-mediated regulation of microtubules is responsible for cell–cell and cell–ECM adhesion and multicellular architecture, and the mechanisms underlying this potential pathway remain mainly hypothetical.

General comments

1. The topic of this study is centered on the regulation of tubulin and microtubules. However, it is unclear whether total tubulin levels, soluble tubulin, or microtubules are involved in the observed processes. The authors should show images of the microtubule network in the different conditions and a quantification of tubulin protein levels. As it is, only one image of microtubule immunofluorescence is shown (Figure 1H), which does not allow the reader to distinguish any microtubules, let alone acetylated ones.
2. Showing the microtubule network and how it is modified or rescued by nocodazole, TOO7.1, or taxol treatment would be essential to strengthen the authors' conclusions that TTC5 acts through this regulation. Does TTC5 only regulate tubulins? Does it have other targets? If so, can we exclude their contribution to the observed phenomena?
3. If TTC5 acts through microtubule stabilization or acetylation, can the effects be mimicked by increasing microtubule acetylation (e.g., using an HDAC6 inhibitor)? The effects of nocodazole and taxol can be very different depending on the concentration used. Here, the concentrations of both taxol and nocodazole are very low, which should result in similar effects on microtubule dynamics, both stabilizing microtubule dynamics (doi: 10.1038/nrc1317, for instance). The sentence (line 210) 'Restoring microtubule dynamics in TTC5 KO spheroids (low-dose nocodazole)…' is an overstatement unless the authors actually show that nocodazole treatment rescues EB1 dynamics and acetylation levels.
4. Do these low doses of taxol and nocodazole affect tubulin expression levels by a TTC5-dependent autoregulation, which would change significantly the interpretation of the results? Or do they act only downstream of TTC5-dependent autoregulation by correcting microtubule dynamics? Can TTC5 KO effects be rescued by a downregulation of tubulin expression?
5. The conclusion (line 187) that 'tubuline autoregulation is critical for maintaining proper cell–cell and cell–ECM adhesion' is not supported by clear data. Here, adhesion is not measured. Only N-cadherin, fibronectin, and α5 integrin levels are shown, and the staining is not very clear. Can the authors show the protein levels by Western blot? The entire study is performed on spheroids. Are the effects shown here limited to the 3D situation? If cell–cell and cell–ECM adhesions are perturbed, one might also expect to see this in 2D, where the imaging may be more demonstrative.
6. What is the molecular mechanism underlying the effect of TTC5 on the downregulation of N-cadherin, integrins, and fibronectin? Is this a transcriptional regulation, or does it rely on microtubule-dependent transport? If so, why are the immunofluorescence levels lower? Is it possible to mimic TTC5 KO effects by inhibiting kinesins?
7. The statistical tests used in the study are not appropriate. The authors frequently use the two-tailed Student's t-test, while non-parametric tests should be used. Most of the time, there are several conditions compared, so the Kruskal–Wallis test should be used (for instance, in Figure 1C, 1D). Moreover, it seems that although there are N = 3 independent experiments (Figure 1G, 1I), the authors used n instead of N for the statistical analysis. This is also valid for Figures 2, 3, and 4.

Specific comments

Fig. 2D. Can the authors show a higher-magnification image of the periphery of the spheroids? cells seem to form a ring at the periphery of the spheroid, but it is not clear whether they are at the very edge or slightly behind.
Fig. 3. Can the authors show the effect of E-cadherin OE on the propidium iodide staining, as in Figure 2, where the effects are much clearer?
Fig. 4B. For consistency, can the authors show the corona results as in Figures 2B and 3G?
Extended Data Fig. 3E–G. The migration assay is not very convincing. How do the authors define the "migration area"? Is it normalized by the initial size of the spheroid or by the number of living cells? It is also very surprising that cell migration is unaffected if both cell–cell adhesion and cell–ECM adhesion are altered.
Extended Data Fig. 6B. Which statistical test has been used?

Reviewer #4

(Remarks to the Author)

Lines 96-98 - "Mass Spectrometry corroborated the enrichment of most a- and b-tubulin isotypes in TTC5 KO spheroids, albeit to a varying extent (Fig. 1E), strengthening the hypothesis that tubulin autoregulation is required to maintain tubulin quantity."
- The graph shows most of the tubulins are below the q-value cut-off score, so, please reword the phrase to more accurately describe the data in Fig. 1E.

Also, please indicate the number of biological replicates for the experimental data presented in Fig 1E.

For the Mass Spectrometry Methods section, the data analysis section lacks several critical details required for reproducibility under current proteomics reporting standards (Molecular & Cellular Proteomics, 2023).

Please include:
Database release date
Number of protein entries searched
Software versioning

Please specify whether it’s Spectronaut’s internal implementation or an external mProphet post-processor.

For the FASTA Search Parameters please include:
Precursor and fragment mass tolerances (ppm values).
Charge range searched.
Minimum/maximum peptide length.
Minimum number of peptides per protein.

Data Deposition:
Please deposit all raw DIA files, Spectronaut project files, and search outputs in a ProteomeXchange repository (PRIDE, MassIVE, jPOST, Panorama Public, etc.) and provide an accession number (e.g., PXD#######) and password if under review.

Quantification:
Please include the quantified signal type and the number/type of fragment ions retained per precursor. For protein, state the protein inference rule, shared-peptide handling, and the aggregation scheme.

The Supplemental Table
Only summary differential statistics are provided (log_2 fold-change and q-values). Please include the protein intensity data for each protein within each sample.

Version 1:

Reviewer comments:

Reviewer #1

(Remarks to the Author)
In the revised manuscript, the authors have adequately addressed all of my concerns

Reviewer #2

(Remarks to the Author)
The authors have addressed my concerns and I now support publication of this manuscript in Nature Communications.

Reviewer #3

(Remarks to the Author)
I thank the authors for their thorough responses to my comments and questions. These have satisfactorily addressed my concerns and improved the manuscript, which I now consider suitable for publication.

Reviewer #4

(Remarks to the Author)
No further comments.

Open Access This Peer Review File is licensed under a Creative Commons Attribution 4.0 International License, which permits use, sharing, adaptation, distribution and reproduction in any medium or format, as long as you give appropriate credit to the original author(s) and the source, provide a link to the Creative Commons license, and indicate if changes were made.
In cases where reviewers are anonymous, credit should be given to 'Anonymous Referee' and the source.
The images or other third party material in this Peer Review File are included in the article’s Creative Commons license, unless indicated otherwise in a credit line to the material. If material is not included in the article’s Creative Commons license and your intended use is not permitted by statutory regulation or exceeds the permitted use, you will need to obtain permission directly from the copyright holder.
To view a copy of this license, visit https://creativecommons.org/licenses/by/4.0/

Almeida et al, 2025

Tubulin autoregulation tunes microtubule dynamics to support multicellular architecture and viability

Reviewer #1 (Remarks to the Author)

The manuscript by Almeida et al. investigates tissue-level consequences of tubulin autoregulation using HeLa cell spheroids as a 3D multicellular model. The authors report that disrupting tubulin autoregulation by knocking out TTC5, a protein that recognizes tubulin mRNAs and recruits other proteins leading to mRNA degradation, causes elevated cytosolic tubulin levels, microtubule network hyperstabilization and altered tissue architecture due to altered cell adhesions and cell death in the spheroids. The study aims to shed light on the biological functions of tubulin autoregulation in 3D tissues, which have mostly been studied thus far in 2D cultures and under conditions of microtubule drug treatments to artificially induce tubulin autoregulator pathways. Overall, this work contributes to our understanding of how global tubulin levels are tuned to support tissue dynamics and architecture.

This is an interesting study addressing the biological significance of tubulin autoregulation, a process that remains largely unexplored. A key strength is the use of a tractable HeLa cell spheroid system to test tubulin autoregulatory mechanisms in a 3D context, which has not been previously examined. Notably, disrupting autoregulation leads to a modest increase in total tubulin and selective enrichment of certain isoforms, but not others. The resulting decrease in cell viability upon disruption of autoregulation highlights the importance of appropriately sensing and regulating soluble tubulin levels in tissue-like environments. Several issues limit the impact of the work in its current form, particularly concerning the link between disrupted autoregulation, altered tubulin levels, and microtubule stability. Despite elevated acetylation (a marker of microtubule stability) microtubule growth lifetimes remain unchanged, and, counter intuitively, growth rates are slower despite higher total tubulin levels. Thus, the connection between altered tubulin regulation and microtubule stability or dynamics is not clearly established, and it remains unclear whether the reduced viability arises directly from altered microtubule stability or through indirect effects. With additional quantification and a clearer interpretation of the tubulin and stability data, however, the study could make a valuable contribution to understanding how autoregulation influences microtubule organization and function in 3D contexts.

Major comment.

<u>Figure 1.</u>

The authors report that TTC5 KO increases tubulin mRNA and soluble tubulin in day 10 spheroids. However, the statement "Mass Spectrometry corroborated the enrichment of most a- and b-tubulin isotypes in TTC5 KO spheroids" overstates the result as only a subset of isotypes (4 of 12) are significantly enriched in Fig. 1E. Furthermore, quantification of total tubulin protein levels suggests that they are only mildly increased (P=0.05, which is just approaching statistical significance) in TTC5

KO cells (extended fig 1D). Please change the wording to reflect the modest effect on total tubulin levels and that this is restricted to certain enriched isotypes – perhaps it is the relative tubulin isotype composition that is important, rather than the global levels per se? In Fig. 1D/E, TUBA4A protein is increased in TTC5 KO spheroids while its mRNA is not statistically elevated. Please discuss possible reasons (e.g., post-transcriptional regulation, increased protein stability / changes in protein degradation pathways). Extended Fig. B and C lack statistical significance reporting, so while we can see from 1E that TUBB and TUBAlB protein levels are not elevated in TTC5 KO, it's not clear how this relates to mRNA levels. Why are some isotypes affected more than others? Overall, the findings could be made clearer by explicitly stating which tubulin isoform mRNAs are significantly changed and which are not, and then mapping which mRNA changes correspond to which tubulin protein changes (and which do not).

We have revised the text to explicitly indicate which tubulin isotypes are significantly enriched by mass spectrometry and to clarify that this enrichment is restricted to a subset of isotypes rather than reflecting a uniform increase across all tubulin isotypes (lines 92-98 and 100-102).
To strengthen the correspondence between mRNA and protein data, we included an additional biological replicate for TUBB6 and TUBA4A (now updated in Fig. 1C-D). We also performed additional mRNA measurements for the other isotypes significantly enriched at the protein level in TTC5 KO spheroids (TUBA3C and TUBB4A). The trends remained consistent, and these data are now shown in Extended Data Fig. 1C-D. Moreover, we now provide a plot comparing mRNA and protein fold changes (KO *versus* parental cells) for individual tubulin isotypes (Extended Data Fig. 1I), revealing a strong concordance whereby isotypes with increased transcript levels also show increased protein abundance. In contrast, those isotypes that do not change at the mRNA level remain unchanged at the protein level.
In the Discussion section, we now explicitly discuss the differential behavior of tubulin isotypes. In particular, we propose that variation in isotype responsiveness may reflect differences in translation rates and dynamics, given the co-translational nature of tubulin autoregulation. We further note that isotype-specific features, such as mRNA-associated factors, may contribute to the observed variability, although these remain to be identified (lines 289-295).
Finally, statistical annotations, including non-significant comparisons, have been added to Extended Data Fig. 1E–F.

The authors interpret increased acetylated-tubulin staining as "hyper-stabilized" microtubules. However, their live-imaging quantification at day 5 shows unchanged growth lifetime (i.e. time spent in the growth phase equating to stability) while reporting slower growth rates. Acetylation is commonly taken as a marker enriched on longer-lived, low-turnover microtubules, but unchanged growth lifetime argues against an overall increase in stability. A simple prediction based on in vitro data would be that

elevated tubulin concentration alone accelerates microtubule growth. Therefore, the manuscript should discuss this apparent contradiction and offer plausible mechanisms. Two technical limitations render the current interpretation uncertain (i) acetylation was quantified only at the cortex, whereas the dynamics measurements (e.g., Fig. 1F) appear to have been taken across the cytoplasm, meaning distinct microtubule populations are being compared, and (ii) microtubule dynamics were measured in day 5 spheroids, while protein and mRNA levels were measured at day 10. I recommend the authors provide whole-cell quantification of acetylated tubulin normalized to total tubulin to determine whether the increase is global or cortex-restricted, and the authors should measure microtubule dynamics at day 10 (when tubulin mRNA and protein were quantified) otherwise the link between tubulin protein levels and microtubule dynamics remains speculative. Including orthogonal stability assays (e.g., nocodazole or cold treatment) would also help to substantiate the findings; or at minimum, acknowledge that increased acetylation may reflect a redistribution of stable microtubule subpopulations rather than a cell-wide increase in stability.

We have revised the manuscript to address the apparent discrepancy between increased tubulin levels and reduced microtubule growth rates. While *in vitro* studies show that microtubule polymerization scales with tubulin concentration, in cells, this relationship is less straightforward, in part because microtubule growth is shaped by a complex network of regulatory factors. These include the availability of nucleation sites (e.g., γ-TuRCs, augmin) and the activity of microtubule-associated proteins (MAPs) that regulate tubulin incorporation, stabilization, or sequestration (e.g., chTOG, CLASPs, stathmin). We now explicitly discuss that altered MAP activity or tubulin partitioning could account for reduced growth rates despite increased tubulin levels (lines 319-326).

We also revisited our analysis of microtubule dynamics. In the original analysis, growth lifetime was constrained by the requirement to track EB3 comets for a minimum of four consecutive frames (4.5 seconds), limiting sensitivity to detect differences in this parameter. We therefore removed the lifetime analysis and instead focused on growth speed and growth length over a defined time window of 4.5-7.5 seconds. These revised analyses show a significant reduction in TTC5 KO cells (Fig. 1G and Extended Data Fig. 3B).

To further assess microtubule stability, we treated cells with nocodazole (1 or 2 µM, 1 hour) and performed live-cell imaging of EB3-EGFP. After treatment with the highest dose of nocodazole, TTC5 KO cells exhibited a higher EB3 comet density compared to parental cells (0.15 vs 0.12 comets/μm^2), consistent with increased resistance to microtubule depolymerization and suggesting altered microtubule stability (Extended Data Fig. 3E-F; lines 127-133).

Tubulin acetylation was quantified specifically at the cell cortex. We chose this approach to avoid confounding effects arising from differences in nuclear-to-cytoplasmic ratios between parental and TTC5 KO cells, which alter the relative area occupied by the microtubule network (see panel below). We now clarify this quantification strategy in the Methods (lines 555-558) and acknowledge in the Discussion that increased acetylation may reflect a localized redistribution of stable microtubule populations rather than a uniform, cell-wide increase in stability (lines 335-339).

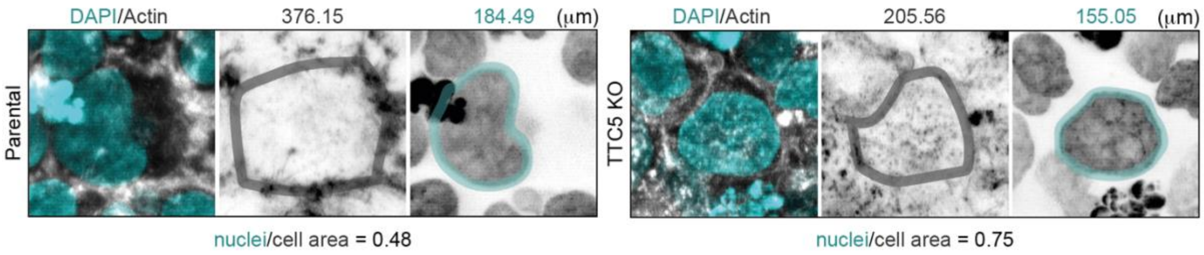

Finally, microtubule dynamics were measured at day 5 spheroids due to technical constraints associated with transient EB3-EGFP expression, which becomes too weak for reliable tracking at later stages. To address the temporal mismatch with mRNA and protein measurements (day 10), we now provide tubulin mRNA levels (TUBA4A and TUBB6) across spheroid development (day 2 to 10; Extended Data Fig. 1G-H), showing that transcript levels are consistently elevated throughout this period. We further show that the mRNA and protein levels show a high level of correlation (Extended Data Fig. 1I). Together, these data support the interpretation that the observed effects on microtubule dynamics are maintained across spheroid development.

Figure 2.

By day 10, the authors state that the corona area in TTC5KO spheroids "equaled or exceeded" the size of the core. However, quantification in Fig. 2B and Extended Fig. 2C suggests that while the corona is larger in TTC5KO than in parental spheroids, it does not exceed the size of the core. A clearer way to represent this would be to plot the core-to-corona area ratio for each spheroid, which would allow direct visualization of relative expansion and clarify whether the corona truly surpasses the core in size, if that is relevant.

We have now clarified that corona expansion increases the overall spheroid area in TTC5 KO and mutant spheroids, but does not exceed the size of the core (lines 149-151).
We chose to retain the stacked representation of core and corona areas because it allows direct comparison of both compartments across genotypes. In contrast, a core-to-corona ratio would collapse these two parameters into a single value and could be difficult to interpret, as an increased ratio may result either from a larger corona or from a smaller core. We therefore consider the current representation to provide a more informative view of the phenotype.

For the viability data (Fig. 2C), please clarify whether the luminescence (ATP) readouts were normalized to total cell number per spheroid, as differences in ATP content could otherwise reflect variation in cell number rather than true differences in viability. Regarding the statement that "cell detachment and accumulation at the spheroid corona preceded cell death (Extended Data Fig. 3D), implicating defective adhesion rather than necrotic core dispersion," only a single example is shown. This claim should be quantified, as currently only a single example is shown. Finally, it is important to clarify whether dying or apoptotic cells were excluded from the analyses of microtubule acetylation, as cell death could independently alter microtubule organization. This distinction is crucial to determine whether the observed microtubule phenotype represents a cause or consequence of reduced viability.

Because ATP-based luminescence measurements can be influenced by total cell number, we now interpret this assay as reflecting a combination of cell viability and spheroid size. To address this, we complemented these measurements with orthogonal live/dead staining using Hoechst and propidium iodide (PI), which directly assesses cell viability at the single-cell level. These analyses confirm increased cell death in TTC5 KO spheroids independently of ATP measurements and are now emphasized in the Results (lines 158-167).
To address the concern regarding cell detachment and death, we have now quantified this process. Cells accumulating at the spheroid corona were tracked over time, and their probability of survival was measured. This analysis shows detachment and accumulation at the periphery, followed by a progressive decrease in survival probability, indicating that detachment precedes cell death. These data are now included in Extended Data Fig. 5E and described in the Results (lines 176-180).
In the Methods section, we now state explicitly that, based on DAPI staining and the characteristic chromatin compaction, dead cells were excluded from all analyses, including tubulin acetylation and cell adhesion quantifications (lines 550-552).

Figure 3.
The quantification of data in 3A-B (and corresponding extended data) appears to rely on a line scan from cell center to periphery, which could be problematic when the cortical signal is non-homogeneous; perhaps a more representative metric would be to measure fluorescence intensity along the entire cell perimeter (as done for acetylated-tubulin in Fig. 1).
Figure 3E – Could the observed cell rounding and loss of cell–cell junctions also reflect activation of an early cell death program (e.g. apoptosis induced by tubulin imbalance, leading to secondary junction loss) rather than a direct effect of altered tubulin levels on adhesion complexes? Can the authors distinguish between these possibilities, for example, by assessing earlier apoptosis markers (e.g.,

caspases, Annexin V, or nuclear morphology) in these conditions? Why is the aspect ratio phenotype not rescued with WT TTC5 in the graph in 3E?

It would be helpful to also show the state of the microtubule network in the same cells displayed in Figs. 3A and C. This would strengthen the connection between tubulin levels, microtubule organization, and adhesion phenotypes.

We have retained the cortex-to-cytoplasm fluorescence intensity ratio, as this metric directly captures changes in subcellular localization—specifically, redistribution from the cortex to the cytoplasm—rather than changes in total protein abundance. This rationale is now clarified in the Methods section (lines 562-566). In this case, the non-homogeneous localization of fibronectin does not pose a bias to our analysis, because its localization is vastly lost in TTC5-deficient and mutant spheroids.

To determine whether the observed cell rounding and loss of cortical adhesion markers reflect an early apoptotic response, we stained spheroids for the apoptosis marker cleaved caspase-3 (c-CASP3) and the adhesion molecule N-cadherin. TTC5 KO cells that appeared rounded and showed reduced cortical N-cadherin staining were negative for c-CASP3, indicating that loss of adhesion precedes, rather than results from, apoptosis. These data are now included in Extended Data Fig.7A and described in the Results (lines 210-214).

The TTC5^{WT} rescue condition corresponds to overexpression of wild-type TTC5. As this may itself perturb cellular homeostasis, it likely accounts for the partial rescue observed in the aspect ratio quantification. This point is now explicitly stated in the text (lines 206-209). Statistical analyses, however, confirm that there are no significant differences between parental and TTC5^{WT} cells in the updated Fig. 3E.

The experimental conditions used in Fig. 3A and 3C are identical to those in Fig. 1H-I, where microtubule organization and acetylation were characterized. The possible link between altered microtubule organization and adhesion phenotypes is highlighted in the discussion section (lines 334-342).

Figure 4.

Figure 4 requires clarification before the claims about a restored cortical corona and N-cadherin localization are convincing. I cannot see a defined "corona" in the images shown in Fig. 4A; it is not clear which feature the authors are labeling as the corona nor why day 7 spheroids were used here when the manuscript otherwise emphasizes day 10 as the point where phenotypes are most robust (Fig. 2). Please repeat the analysis at day 10 (or justify the choice of day 7) and include clearer annotations that define the corona and its boundaries as well as quantification of both core and corona area.

For Fig. 4D, the statement that "low-dose nocodazole reinstated cortical N-cadherin localization" requires statistical support: is the cortical N-cadherin signal in parental + DMSO statistically non significantly different from TTC5KO + low-dose nocodazole? Are integrin and fibronectin localization,

and cell viability are also restored by nocodazole treatment? Have the authors measured the additional effects of nocodazole and taxol on cell migration and division in these experiments, which also contribute to tissue architecture and integrity.

We have now improved the visualization of the corona in Fig. 4A by adding a dashed line to clearly delineate the core-corona boundary. Microtubule-targeting agents were added at day 5, before corona formation and the associated decline in cell viability. As a result, analysis was performed at day 7 to capture the effects of these treatments during spheroid development. At later time points (e.g., day 10), extensive cell death in untreated TTC5 KO spheroids complicates interpretation. This rationale is now clarified in the text (lines 231-233).

In Fig. 4D, we now include a direct statistical comparison between microtubule growth speed in Parental + DMSO and TTC5 KO + low-dose nocodazole conditions, which shows that there is no statistically significant difference between these two experimental conditions.

In addition, we extended the analysis to other adhesion markers: fibronectin localization is also restored upon nocodazole treatment in TTC5 KO spheroids and lost after taxol-induced microtubule stabilization in parental cells (Extended Data Fig. 8G-H).

With respect to additional cellular processes, we did not detect baseline differences in proliferation or migration between parental and TTC5 KO spheroids (Extended Data Fig. 5A-C and 5F-H). These observations suggest that altered adhesion, rather than changes in migration or division, is the primary driver of corona expansion.

Finally, penetration of microtubule-targeting agents in 3D spheroids is a concern. Have the authors verified that the drug treatments alter microtubule dynamics throughout the spheroid (edge versus intermediate versus core) rather than only at the periphery. Without this, it is difficult to determine whether rescue of adhesion/corona phenotypes is due to a uniform effect on microtubules throughout the spheroid tissue.

Drug penetration into spheroids has been rigorously validated in our previous work using the same system and slightly bulkier small-molecule compounds, demonstrating efficient penetration through the corona and proliferative outer layers, and detectable, albeit reduced, penetration into the spheroid core (von Glasenapp et al., Nature Communications, 2025). To directly support our conclusion that modulation of microtubule dynamics underlies the rescue of adhesion and corona phenotypes, we generated HeLa stable cell lines expressing EB3-EGFP and measured microtubule growth dynamics in spheroids treated with DMSO, low-dose nocodazole, or taxol (Figure 4C-D and Extended Data Fig. 8A-B). In both parental and TTC5 KO spheroids, low-dose nocodazole increased microtubule growth speed, whereas taxol reduced growth speed, consistent with their respective effects on microtubule

dynamics. Due to imaging constraints, these measurements were performed at the spheroid periphery, which is now explicitly stated in the manuscript (lines 538-539).

While we cannot directly assess drug effects in the spheroid core, the consistent phenotypic rescue of adhesion and corona organization suggests that modulation of microtubule dynamics in accessible regions is sufficient to influence overall spheroid architecture.

Minor comments:

1. Y-axis on graphs in 3B/D should say cortex-to-cytoplasm F.I ratio. This is now rectified.
2. Y-axis in Figure 2C should state what is actually being measured (ATP luminescence?) We corrected the Y-axis label.
3. Missing citation for Walker et al., J Cell Biol 1988, in introduction, as demonstration of microtubule growth rate scaling with tubulin concentration in vitro. The citation was added in line 34.
4. The spheroid migration assay requires a more detailed methodological description and definition of the "migration area" for those not familiar with the assay. Do individual cells migrate within the spheroids, and could this be measured as well? Additional methodological details were added to the Methods section (lines 595-601). Only cells that migrated out of the spheroid (3D) onto the Matrigel-coated dish (2D) were detected and quantified. Individual cell migration within intact spheroids can be assessed by single-cell tracking; however, we did not observe directional migratory behavior of HeLa cells when monitoring cell detachment and death events.
5. Microtubule growth length is presumably calculated over a set period of time but this is not noted, and should be clarified. Given that they report no effect of TTC5 KO on microtubule lifetimes, this is essentially another way of reporting the growth rate.

This point is addressed in our response to Fig. 1. Briefly, we have clarified that microtubule growth length was measured over a fixed interval and revised the analysis accordingly, focusing on growth speed and growth length parameters. These details are now explicitly described in the Methods (lines 533-535).

Reviewer #2 (Remarks to the Author):

In this study, Almeida et al. explore the biological significance of tubulin autoregulation, a feedback mechanism that maintains proper levels of α- and β-tubulin proteins. Using human 3D (HeLa) cell spheroids, the authors show that loss of TTC5 (by knockout (KO)) or TTC5 mutants that disable tubulin autoregulation cause accumulation of soluble tubulin (mRNA and protein). Accumulation of α- and β-tubulin, in turn, leads to decreased microtubule growth rates (assessed by EB3 tracking), increased acetylation of α-tubulin K40, and a redistribution of microtubules towards the cortex. The authors then show that tubulin autoregulation defective spheroids exhibit an abnormal architecture characterized by a dispersive "corona" of detached cells; detached cells have an increased propensity to die. Detachment appears to be caused by impaired cell-cell and cell-matrix adhesion; TTC5 KO and TTC5 mutant

spheroids showed reduced cortical localization of N-cadherin, integrin a5, and fibronectin relative to wild type spheroids. The effects of deleting TTC5 or tubulin autoregulation defective TTC5 mutants could be rescued by reintroducing functional TTC5, E-cadherin overexpression, or modulating microtubule dynamics with mild destabilization (nocodazole). In contrast, taxol, which stabilizes microtubules, worsened phenotypes caused by defects in tubulin autoregulation. In summary, the authors demonstrate that tubulin autoregulation is important for fine-tuning microtubule dynamics, and that this in turn maintains cell-cell and cell-matrix adhesion, specifically in spheroids. This work suggests that tubulin autoregulation safeguards spheroid organization, connecting molecular control of tubulin synthesis to higher-order structural integrity.

Concerns

1. Previous work by Lin et al. (2020) (Gasic was one of the investigators on this manuscript) showed that defects in tubulin autoregulation did not change tubulin protein levels in monolayer cultures (tubulin mRNA levels did increase), which is at odds with findings in this work. The authors speculate that cell context is important, that is, cells organized into a 3D structure respond differently than cells in 2D. This explanation is not terribly satisfying, but I understand that it may be beyond the scope of this paper to determine the mechanism(s) that underlies this difference. It would be worthwhile, however, to show that a cell line other than HeLa responds in a similar manner.

To address the reviewer's concern, we extended our analysis to an independent cell line. We cultured HEK293 cells under 3D conditions, and measured tubulin mRNA and protein levels in parental and TTC5 KO spheroids, as well as TTC5 KO spheroids stably re-expressing $TTC5^{WT}$ (Extended Data Fig. 2C-D). We observed a similar increase in tubulin mRNA and protein levels upon loss of TTC5 in this system, a phenotype that is rescued by the re-expression of $TTC5^{WT}$ and consistent with our findings in HeLa spheroids.

These results indicate that the effect of tubulin autoregulation on tubulin protein levels in 3D is not cell line-specific and support the notion that cellular context is relevant.

2. It is puzzling that microtubule growth rates are slower in tubulin autoregulation defective cells. In many (most) contexts, microtubule growth rates mirror soluble tubulin concentration: microtubules grow faster as tubulin levels increase. The authors offer no explanation for this paradox. Please address.

This point overlaps with a concern raised by Reviewer 1 regarding Figure 1 of our manuscript. We have revised the manuscript to address the apparent discrepancy between increased tubulin levels and reduced microtubule growth rates. While *in vitro* studies show that microtubule polymerization scales with tubulin concentration, in cells, tubulin is typically present below the critical concentration, and microtubule growth is often limited by additional factors. These include the availability of nucleation

sites (e.g., γ-TuRCs, augmin) and the activity of microtubule-associated proteins (MAPs) that regulate tubulin incorporation, stabilization, or sequestration (e.g., chTOG, CLASPs, stathmin). We now explicitly discuss that altered MAP activity or tubulin partitioning could account for reduced growth rates despite increased tubulin levels (lines 319-326).

3. The authors show western blot data for alpha-tubulin (Extended Data Figure 1D) to argue that tubulin levels are elevated. Please also show western blot data for beta-tubulin.

We have now included western blot data and quantification for β-tubulin (Extended Data Fig. 2B). Although the difference did not reach statistical significance, the data suggest a modest increase (~20%) in β-tubulin levels in tubulin autoregulation-deficient spheroids.

4. In Figure 4, the authors show that nocodazole rescues the dispersed phenotype of tubulin autoregulation spheroids, whereas taxol worsens the phenotype. The authors speculate that low dose nocodazole restores microtubule dynamics, which I take to mean that microtubule growth rates should increase in the presence of low dose nocodazole. This is counterintuitive (see Point 2). It would be useful to measure microtubule growth rates (by tracking of EB3-GFP comets as performed in Figure 1) in nocodazole- and taxol-treated spheroids.

To address this concern, we generated HeLa cells stably expressing EB3-EGFP and measured microtubule growth dynamics in parental and TTC5 KO spheroids treated with DMSO, nocodazole, or taxol. Low-dose nocodazole increased microtubule growth speed (statistically significant in TTC5 KO spheroids), consistent with enhanced microtubule dynamicity, whereas taxol reduced growth speed, indicative of increased microtubule stability (statistically significant in parental spheroids). The corresponding data are shown in Figure 4C-D and Extended Data Figure 8A-B and described in the results section (lines 240-247).
These measurements demonstrate that, under the conditions used, nocodazole and taxol exert opposite effects on microtubule dynamics in our system. Importantly, restored growth dynamics by nocodazole correlate with rescued spheroid architecture, supporting a direct link between microtubule dynamics and the observed phenotype.

5. It is not clear if the authors omitted mitotic cells when quantifying the levels of K40 acetylated alpha-tubulin. Mitotic spindle microtubules are highly acetylated (Figure 1H), so the authors should comment on how they dealt with dividing cells (~5-10% for HeLa cells in monolayer).

Mitotic cells were excluded from all quantitative analyses, including K40 acetylated alpha-tubulin. We identified mitotic cells based on their characteristic morphology of the DNA and the presence of the mitotic spindle. This is now explicitly stated in the Methods (lines 550-552).

6. It does not appear to me that reintroduction of TTC5 into TTC5 KO cells rescues the levels of fibronectin in spheroid cultures (Figure 3C).

The representative image shown in Figure 3C has been replaced with a more representative example. An additional biological replicate was added to the analysis, strengthening the reproducibility and statistical significance of the rescue effect observed in $TTC5^{WT}$ spheroids.
In addition, we now clarify in the text that the $TTC5^{WT}$ rescue line corresponds to overexpression of wild-type TTC5, which may explain why these cells displayed an intermediate phenotype (lines 206-209). However, this apparent partial rescue did not reach statistical significance compared to the control condition.

7. There are no data (western blotting, or cell imaging of fluorescent E-cadherin) to show that E-Cadherin is actually overexpressed in HeLa cells (Figures 3F-H).

We provide live-cell imaging data showing E-cadherin expression in both parental and TTC5 KO spheroids (Extended Data Fig. 7B). These images demonstrate a clear E-cadherin signal and its correct localization at cell-cell junctions upon overexpression.

Reviewer #3 (Remarks to the Author):
In this manuscript, Ana Almeida and colleagues study the role of tubulin autoregulation, a feedback mechanism controlling tubulin protein synthesis, in maintaining microtubule dynamics, cell adhesion, and tissue integrity. By using 3D human cell spheroids, they show that loss of the key regulator TTC5 disrupts this pathway, leading to excess tubulin, hyperstable microtubules, and compromised cell–cell and cell–matrix adhesion, disorganized multicellular architecture and reduced cell viability. This work may contribute to a better understanding of diseases involving TTC5 or SCAPER and may open new avenues for targeted therapies that modulate microtubule stability or tubulin levels. While the topic is potentially interesting, the authors fail to clearly demonstrate their claim that TTC5-mediated regulation of microtubules is responsible for cell–cell and cell–ECM adhesion and multicellular architecture, and the mechanisms underlying this potential pathway remain mainly hypothetical.

General comments
1. The topic of this study is centered on the regulation of tubulin and microtubules. However, it is unclear whether total tubulin levels, soluble tubulin, or microtubules are involved in the observed

processes. The authors should show images of the microtubule network in the different conditions and a quantification of tubulin protein levels. As it is, only one image of microtubule immunofluorescence is shown (Figure 1H), which does not allow the reader to distinguish any microtubules, let alone acetylated ones.

The distinction between total, soluble, and polymerized tubulin is an important point. Biochemical fractionation to directly measure soluble versus polymerized tubulin pools is technically challenging in 3D spheroid systems and cannot be performed reliably without disrupting the architecture and microtubule network.

To address this question functionally, we leveraged multiple orthogonal perturbations (Fig. 4). First, reducing excess tubulin levels in TTC5 KO spheroids restores normal spheroid architecture, indicating that elevated tubulin abundance is causative. Second, directly modulating microtubule dynamics rescues the phenotype, demonstrating that microtubule behavior is a key downstream effector. Finally, inducing similar changes in microtubule dynamics in parental spheroids is sufficient to recapitulate the spheroid corona phenotype.

Together, these complementary approaches establish a functional link between tubulin levels, microtubule dynamics, and spheroid organization, even in the absence of direct biochemical measurements of tubulin partitioning.

To improve visualization of the microtubule network, we have now included additional representative images and adjusted display settings to better resolve microtubule structures (Extended Data Fig. 3C).

2. Showing the microtubule network and how it is modified or rescued by nocodazole, TOO7.1, or taxol treatment would be essential to strengthen the authors' conclusions that TTC5 acts through this regulation. Does TTC5 only regulate tubulins? Does it have other targets? If so, can we exclude their contribution to the observed phenomena?

Because we propose that altered microtubule dynamics are the primary cause for the phenotypes observed, to visualize the microtubule network under the different treatment conditions, we have now included EB3-EGFP live-cell imaging and measured microtubule growth speed in control and drug-treated spheroids (Fig. 4C-D, and Extended Data Fig. 8A-B). At the low concentrations of taxol used in this study, the parental cells display microtubule dynamics comparable to control-treated TTC5 KO cells. Conversely, at low concentrations of nocodazole, TTC5 KO cells display microtubule dynamics comparable to untreated parental cells. These data strengthen our conclusion that TTC5 acts through microtubule dynamics.

Consistently, western blot analysis did not reveal significant changes in total tubulin levels upon treatment with microtubule destabilizer or stabilizer (Extended Data Fig. 8F), while taxol treatment led to an increase in tubulin acetylation (Extended Data Fig. 8E), in line with enhanced microtubule

stabilization under this condition. Notably, this is also the condition in which cell adhesion defects and corona expansion are exacerbated, supporting a functional link between altered microtubule behavior and the observed phenotype.

TTC5 has been characterized as a factor mediating tubulin mRNA decay through co-translational recognition of nascent tubulin chains. To date, tubulin transcripts are the only established targets of this pathway. While we cannot formally exclude additional substrates, several lines of evidence support that the observed phenotypes are primarily mediated through tubulin regulation. First, the $TTC5^{R147A}$ mutant, which specifically disrupts recognition of the tubulin nascent peptide, fully phenocopies the TTC5 knockout. This mutation is expected to impair TTC5 interaction with tubulin-specific N-terminal motifs, and similar interactions with unrelated nascent peptides are unlikely. Second, the selective changes observed at the level of tubulin mRNA and protein, together with the phenotypic rescue upon restoring tubulin levels or microtubule dynamics (Fig. 4), further support a tubulin-centered mechanism. Together, these findings strongly argue that TTC5 acts predominantly through the regulation of tubulin.

3. If TTC5 acts through microtubule stabilization or acetylation, can the effects be mimicked by increasing microtubule acetylation (e.g., using an HDAC6 inhibitor)? The effects of nocodazole and taxol can be very different depending on the concentration used. Here, the concentrations of both taxol and nocodazole are very low, which should result in similar effects on microtubule dynamics, both stabilizing microtubule dynamics (doi: 10.1038/nrc1317, for instance). The sentence (line 210) 'Restoring microtubule dynamics in TTC5 KO spheroids (low-dose nocodazole)…' is an overstatement unless the authors actually show that nocodazole treatment rescues EB1 dynamics and acetylation levels.

We have now directly measured microtubule dynamics in parental and TTC5 KO spheroids under the indicated treatment conditions using EB3-EGFP live imaging. At the concentrations used, nocodazole increased, whereas taxol reduced microtubule polymerization rates. These data are now summarized in Figure 4C-D and Extended Data Fig. 8A-B. Based on these measurements, we have revised the manuscript's wording to avoid overstatement. We now explicitly state that low-dose nocodazole restores microtubule growth dynamics in TTC5 KO spheroids, rather than inferring this effect (lines 240-243). In addition, we find that taxol treatment leads to a significant decrease in the microtubule growth speed and increase in tubulin acetylation levels (Fig. 4C-D and Extended Data Fig. 8F), consistent with enhanced microtubule stabilization (lines 243-247). Under these conditions, spheroid architecture is altered and resembles the phenotype observed in TTC5 KO spheroids, supporting a link between increased microtubule stability and impaired tissue organization.

Regarding the suggestion to directly modulate microtubule acetylation (e.g., via HDAC6 inhibition), we note that tubulin acetylation is generally considered a consequence of prolonged microtubule lifetime rather than a primary determinant of microtubule dynamics. In this context, acetylation serves

as a marker of stabilized, long-lived microtubules, but does not itself drive changes in microtubule growth behavior. We therefore interpret the increased acetylation observed in TTC5 KO and taxol-treated conditions as a downstream readout of altered microtubule dynamics, rather than a causal factor in the observed phenotype. Accordingly, we focused on directly measuring and perturbing microtubule dynamics, which we show is sufficient to modulate spheroid architecture (Figure 4C-D and Extended Data Fig. 8A-B). These data support a model in which changes in microtubule dynamics, rather than acetylation per se, underlie the observed defects.

Together, these results demonstrate that pharmacological modulation of microtubule dynamics produces distinct and measurable effects in our system, and that restoring or perturbing these dynamics is sufficient to modulate spheroid architecture.

4. Do these low doses of taxol and nocodazole affect tubulin expression levels by a TTC5-dependent autoregulation, which would change significantly the interpretation of the results? Or do they act only downstream of TTC5-dependent autoregulation by correcting microtubule dynamics? Can TTC5 KO effects be rescued by a downregulation of tubulin expression?

Extended Data Fig. 8F shows that low doses of nocodazole and taxol do not significantly alter total tubulin protein levels, likely due to long tubulin protein half-life, and relatively short and mild treatment. Rather, under the conditions used, these compounds act downstream by modulating microtubule dynamics without affecting tubulin abundance.

To directly address whether TTC5 KO phenotypes can be rescued by reducing tubulin levels, we used a small molecule tubulin degrader (T007-1) in TTC5 KO spheroids (Fig. 4A-B). This treatment fully rescues spheroid architecture. In parallel, modulating microtubule dynamics using low-dose nocodazole also rescues the phenotype (Fig. 4).

These results demonstrate that both the upstream perturbation (increased tubulin levels) and the downstream consequence (altered microtubule dynamics) can be independently targeted to restore normal tissue organization. This supports a model in which TTC5-dependent regulation of tubulin levels controls microtubule dynamics, which in turn governs spheroid architecture.

Importantly, these treatments were applied after spheroid formation (day 5), allowing normal early development before perturbation. In contrast, alternative approaches such as tubulin knockdown would be expected to impair spheroid formation at earlier stages, as reported in conditions of strong microtubule depletion (Smyrek et al., Biol Open, 2019)

5. The conclusion (line 187) that 'tubuline autoregulation is critical for maintaining proper cell–cell and cell–ECM adhesion' is not supported by clear data. Here, adhesion is not measured. Only N-cadherin, fibronectin, and α5 integrin levels are shown, and the staining is not very clear. Can the authors show the protein levels by Western blot? The entire study is performed on spheroids. Are the effects shown

here limited to the 3D situation? If cell–cell and cell–ECM adhesions are perturbed, one might also expect to see this in 2D, where the imaging may be more demonstrative.

We have revised the conclusion to reflect our data more accurately (lines 222-224).
Our analysis focuses on the subcellular localization of adhesion proteins rather than their total abundance. Specifically, we quantified cortex-to-cytoplasm fluorescence ratios to assess redistribution of adhesion components from the plasma membrane to the cytosol. This metric directly captures the mislocalization of adhesion proteins (N-Cadherin, Fibronectin, and integrin α5) in TTC5-deficient or -mutant spheroids. Total levels of N-cadherin (CDH2) and integrins (ITGA3, ITGA5, ITGA2, ITGA1, ITG6, ITGA1, ITGAV, ITGB5, ITGB8) are decreased or not altered in TTC5 KO spheroids, according to the mass spectrometry data. This result is now shown in Extended Data Figure 6F. The levels of fibronectin are not included in the analysis, as they were not detected in the LC-MS analysis.
Regarding analysis in 2D conditions, we note that tubulin autoregulation is not strongly engaged in standard 2D HeLa cultures, as we observe minimal, if any, increase in tubulin levels upon TTC5 loss in this context. As a result, we would not expect to observe corresponding adhesion phenotypes under these conditions. Our findings, therefore, highlight the importance of cellular context, with 3D systems revealing functional consequences of tubulin autoregulation that are not apparent in 2D cultures. This point is discussed in the revised manuscript (lines 298-308).

6. What is the molecular mechanism underlying the effect of TTC5 on the downregulation of N-cadherin, integrins, and fibronectin? Is this a transcriptional regulation, or does it rely on microtubule-dependent transport? If so, why are the immunofluorescence levels lower? Is it possible to mimic TTC5 KO effects by inhibiting kinesins?

We have clarified in the revised manuscript that total levels of the analyzed adhesion proteins are reduced in tubulin autoregulation–deficient spheroids, as supported by mass spectrometry data (lines 199-200 and Extended Data Fig. 6F). Together with the imaging data, this indicates that both protein abundance and subcellular localization are affected.
While reduced protein levels can reflect altered transcriptional regulation, the known function of TTC5 is in the post-transcriptional control of tubulin expression via the recognition and binding to the nascent-tubulin motif (Extended Data Fig. 7G). This mechanism does not directly account for transcriptional regulation of adhesion-related genes, making it unlikely that the observed reduction in protein levels is primarily driven by TTC5. On the other hand, we propose that TTC5 is involved via the alteration of microtubule dynamics, which in turn can impair trafficking, delivery, and retention of adhesion components at the plasma membrane. Mislocalization of adhesion complexes may promote their turnover via endocytic or degradative pathways, contributing to the observed decrease in protein abundance.

While perturbation of motor proteins (e.g., kinesins) represents an interesting approach to further dissect this mechanism, directly testing these possibilities falls beyond the scope of the present study. Instead, our data establish a functional link between tubulin autoregulation, microtubule dynamics, and adhesion protein localization, providing a framework for future mechanistic investigations.

7. The statistical tests used in the study are not appropriate. The authors frequently use the two-tailed Student's t-test, while non-parametric tests should be used. Most of the time, there are several conditions compared, so the Kruskal–Wallis test should be used (for instance, in Figure 1C, 1D). Moreover, it seems that although there are N = 3 independent experiments (Figure 1G, 1I), the authors used n instead of N for the statistical analysis. This is also valid for Figures 2, 3, and 4.

Statistical analyses have been revised throughout the manuscript. Normality was assessed for each dataset using the D'Agostino–Pearson test ($n > 10$) or the Shapiro–Wilk test ($n < 10$). Based on these results, appropriate parametric or non-parametric tests were applied. For normally distributed data, statistical significance was assessed using Student's t-test or one-way ANOVA with Holm-Šidák correction for multiple comparisons. For non-normally distributed data, non-parametric tests were used, including Kolmogorov-Smirnov or Kruskal-Wallis tests with Dunn's correction.
In addition, all statistical analyses have been performed using the number of biological replicates (N) when $N \geq 3$. This information has been updated in the Methods (lines 648-664).

Specific comments
Fig. 2D. Can the authors show a higher-magnification image of the periphery of the spheroids? cells seem to form a ring at the periphery of the spheroid, but it is not clear whether they are at the very edge or slightly behind.

Fig. 2D was updated, and we now provide an inset with a zoom of 3x at the periphery of spheroids, with the core-corona transition highlighted with a white dashed line.

Fig. 3. Can the authors show the effect of E-cadherin OE on the propidium iodide staining, as in Figure 2, where the effects are much clearer?

PI and Hoechst staining were used in Fig, 2D-E as an orthogonal approach to validate the ATP-based luminescence assay for assessing cell viability, showing strong concordance between the two methods. Based on this validation, ATP-based assays were used as a robust and quantitative readout for subsequent experiments. For the E-cadherin overexpression condition, we now provide data from three independent experiments, strengthening the reproducibility and statistical significance of the rescue effect observed in TTC5 KO spheroids (Fig. 3H).

Fig. 4B. For consistency, can the authors show the corona results as in Figures 2B and 3G? Extended Data Fig. 3E–G. The migration assay is not very convincing. How do the authors define the "migration area"? Is it normalized by the initial size of the spheroid or by the number of living cells? It is also very surprising that cell migration is unaffected if both cell–cell adhesion and cell–ECM adhesion are altered.

We kept spheroid area measurements in Fig. 2B and related extended data figures to allow direct read-out of both core and corona areas and to highlight the differences imposed by loss of tubulin autoregulation. From this point onward, the corona area is used as a proxy for cell viability and to compare the effects of cadherin overexpression (Fig. 3G), ECM supplementation (Extended Data Fig. 7C), and microtubule-targeting drugs (Fig. 4B and Extended Data Fig.8D).
Additional methodological details have been added to the "Spheroid Migration assay" section (lines 595-601). Briefly, this assay quantifies the spreading of cells from spheroids onto a Matrigel-coated surface following transfer from 3D to 2D conditions. The measured area corresponds to the region occupied by cells that have exited the spheroid and therefore reflects cell spreading and substrate engagement. Epithelial cells can acquire migratory and invasive properties upon loss of adhesion, often through downregulation of E-cadherin and upregulation of mesenchymal markers (e.g., vimentin, fibronectin, and N-cadherin). In TTC5 KO cells, however, the localization of the tested adhesion proteins is compromised, which impairs the establishment of a functional cell migration activity. As a result, the rounded cells likely fail to generate the traction forces required for migration on a 2D substrate, and eventually die, providing a plausible explanation for the observed trend.

Extended Data Fig. 6B. Which statistical test has been used?

This information is now included in the figure legend (Extended Data Fig. 8D). Statistical significance for taxol treatments relative to their respective DMSO controls was assessed using one-way ANOVA followed by Holm–Šidák multiple comparisons test.

Reviewer #4 (Remarks to the Author):
Lines 96-98 - "Mass Spectrometry corroborated the enrichment of most a- and b-tubulin isotypes in TTC5 KO spheroids, albeit to a varying extent (Fig. 1E), strengthening the hypothesis that tubulin autoregulation is required to maintain tubulin quantity." - The graph shows most of the tubulins are below the q-value cut-off score, so, please reword the phrase to more accurately describe the data in Fig. 1E.

The isotypes that are significantly upregulated are explicitly mentioned in the text (lines 100-102).

Also, please indicate the number of biological replicates for the experimental data presente
d in Fig 1E.

The information for the number of biological replicates is now indicated in the figure legend (N=3, lines 906-909).

For the Mass Spectrometry Methods section, the data analysis section lacks several critical details required for reproducibility under current proteomics reporting standards (Molecular & Cellular Proteomics, 2023).

Please include:
Database release date
Taking advantage of the manuscript revision process, the database was updated to its current release, and a new Spectronaut search was conducted. The manuscript is modified accordingly, and the new database release is indicated.

Number of protein entries searched
Homo sapiens reviewed proteome - 20416 entries. This information is now indicated in the Methods section (lines 494-495).

Software versioning
Spectronaut 20 software, release 20.3.251119. This information is detailed in the Methods section (lines 492-495).

Please specify whether it's Spectronaut's internal implementation or an external mProphet post-processor.
Spectronaut's internal implementation was used. This information is now corrected in the revised manuscript (lines 492-495).

For the FASTA Search Parameters please include:
Precursor and fragment mass tolerances (ppm values).
Tolerance parameters were set to dynamic, with the ideal mass tolerance determined after a first-pass calibration (± 40 ppm) (Spectronaut's default). This information is now clarified in the manuscript (lines 500-501).

Charge range searched.

Charge state was not filtered.

Minimum/maximum peptide length.
Peptide length of 7 to 52 residues. This information was added in the revised manuscript.

Minimum number of peptides per protein.
Single-hits were defined by stripped sequence and excluded. This information was added in the revised manuscript.

Data Deposition:
Please deposit all raw DIA files, Spectronaut project files, and search outputs in a ProteomeXchange repository (PRIDE, MassIVE, jPOST, Panorama Public, etc.) and provide an accession number (e.g., PXD#######) and password if under review.

The mass spectrometry proteomics data have been deposited to the ProteomeXchange Consortium via the PRIDE partner repository with the dataset identifier PXD076805. The data can be verified by logging in to the PRIDE website using the following account details:
Username: reviewer_pxd076805@ebi.ac.uk
Password: ksGfkhWWh9hF

Quantification:
Please include the quantified signal type and the number/type of fragment ions retained per precursor. For protein, state the protein inference rule, shared-peptide handling, and the aggregation scheme.

Quantification was based on peak area at the MS2 level for identified precursors (Q-value). No imputation of the missing values was performed. The average of the 3 best peptides was used per protein group. The protein inference rule was computed with the parsimony approach using the IDPicker algorithm (Zhang et al., J Proteome Res, 2007). This information is available in the Methods section.

The Supplemental Table

Only summary differential statistics are provided ($\log_2$ fold-change and q-values). Please include the protein intensity data for each protein within each sample.

Supplementary Table 2 reports protein intensity values for each protein across all samples. The complete dataset is available in the PRIDE repository under accession number PXD076805.
